# Nucleotide de novo synthesis increases breast cancer stemness and metastasis via cGMP-PKG-MAPK signaling pathway

**DOI:** 10.1371/journal.pbio.3000872

**Published:** 2020-11-13

**Authors:** Yajing Lv, Xiaoshuang Wang, Xiaoyu Li, Guangwei Xu, Yuting Bai, Jiayi Wu, Yongjun Piao, Yi Shi, Rong Xiang, Longlong Wang

**Affiliations:** 1 School of Medicine, Nankai University, Tianjin, China; 2 Department of Thyroid and Neck Tumor, Tianjin Medical University Cancer Institute and Hospital, Tianjin, China; 3 The International Collaborative Laboratory for Biological Medicine of the Ministry of Education, Nankai University School of Medicine, Tianjin, China; University of California Los Angeles, UNITED STATES

## Abstract

Metabolic reprogramming to fulfill the biosynthetic and bioenergetic demands of cancer cells has aroused great interest in recent years. However, metabolic reprogramming for cancer metastasis has not been well elucidated. Here, we screened a subpopulation of breast cancer cells with highly metastatic capacity to the lung in mice and investigated the metabolic alternations by analyzing the metabolome and the transcriptome, which were confirmed in breast cancer cells, mouse models, and patients’ tissues. The effects and the mechanisms of nucleotide de novo synthesis in cancer metastasis were further evaluated in vitro and in vivo. In our study, we report an increased nucleotide de novo synthesis as a key metabolic hallmark in metastatic breast cancer cells and revealed that enforced nucleotide de novo synthesis was enough to drive the metastasis of breast cancer cells. An increased key metabolite of de novo synthesis, guanosine-5'-triphosphate (GTP), is able to generate more cyclic guanosine monophosphate (cGMP) to activate cGMP-dependent protein kinases PKG and downstream MAPK pathway, resulting in the increased tumor cell stemness and metastasis. Blocking de novo synthesis by silencing phosphoribosylpyrophosphate synthetase 2 (PRPS2) can effectively decrease the stemness of breast cancer cells and reduce the lung metastasis. More interestingly, in breast cancer patients, the level of plasma uric acid (UA), a downstream metabolite of purine, is tightly correlated with patient’s survival. Our study uncovered that increased de novo synthesis is a metabolic hallmark of metastatic breast cancer cells and its metabolites can regulate the signaling pathway to promote the stemness and metastasis of breast cancer.

## Abstract 2

Metabolic reprogramming to fulfil the biosynthetic and bioenergetic demands of cancer cells is well known, but a role in metastasis is less clear. This study shows that nucleotide metabolic reprogramming leads to an enhancement of the cGMP-PKG-MAPK axis, enabling breast cancer cells to effectively colonize and grow in the lung.

## Introduction

Breast cancer is prone to relapse and metastasize to other organs, such as lung, brain, liver, and bone marrow, which results in worse outcomes in breast cancer patients [1–3]. Therefore, discovering the mechanism of metastasis of breast cancer is especially important for anti–breast cancer treatments. The ability to reprogram metabolism to fulfill the biosynthetic and bioenergetic demands of cancer cells has aroused great interest in recent years [4–7]. Numerous studies have demonstrated metabolic alteration of protein synthesis, glycolysis, glutaminolysis, and nucleotide synthesis in different types of cancer [8–12]. Thus, the investigation of the metabolic alternation in breast cancer progression, especially in metastasis, is very promising for the discovery of new drug target. Recently, many studies in cancer metabolism have focused on the metabolic regulation and crosstalk between signaling pathways and metabolic networks [13]. For example, pathways downstream of oncogenes have been shown to regulate cancer cell metabolism. It has been reported that oncogene c-Myc orchestrates nucleotide biosynthesis by up-regulating the expression of nucleotide synthesis enzyme phosphoribosylpyrophosphate synthetase 2 (PRPS2) [8]. In cancer cells, nucleotide production greatly increases because of the demand of ribosomal RNA, which synthesizes DNA and maintains the transcriptome during the rapid cell proliferation. It also functions as an important downstream of major nutrient pathways [14] and chemotherapeutic target of almost all types of cancer. Thus, nucleotide synthesis is of importance in the cancer cell metabolic network. However, the crosstalk of nucleotide synthesis and signaling pathways that regulate cellular programs is still less clear.

In this work, we isolated a highly metastatic cell population from parent breast cancer cells via an in vivo mouse model and analyzed the whole metabolome, isotope tracing of ^13^C, and transcriptome. We successfully identified highly activated nucleotide de novo synthesis to be a new metabolic hallmark of the lung metastatic breast cancer cells and investigated the underlying molecular metabolism. Lastly, it is worth mentioning that by analyzing the clinical information, we found a high level of plasma uric acid (UA), a downstream metabolite of purine metabolism, correlated with the prognosis of breast cancer patients, suggesting a new prognostic marker for breast cancer.

## Results

### Metastatic breast cancer cells in lung display increased nucleotide de novo synthesis

**Fig 1:**
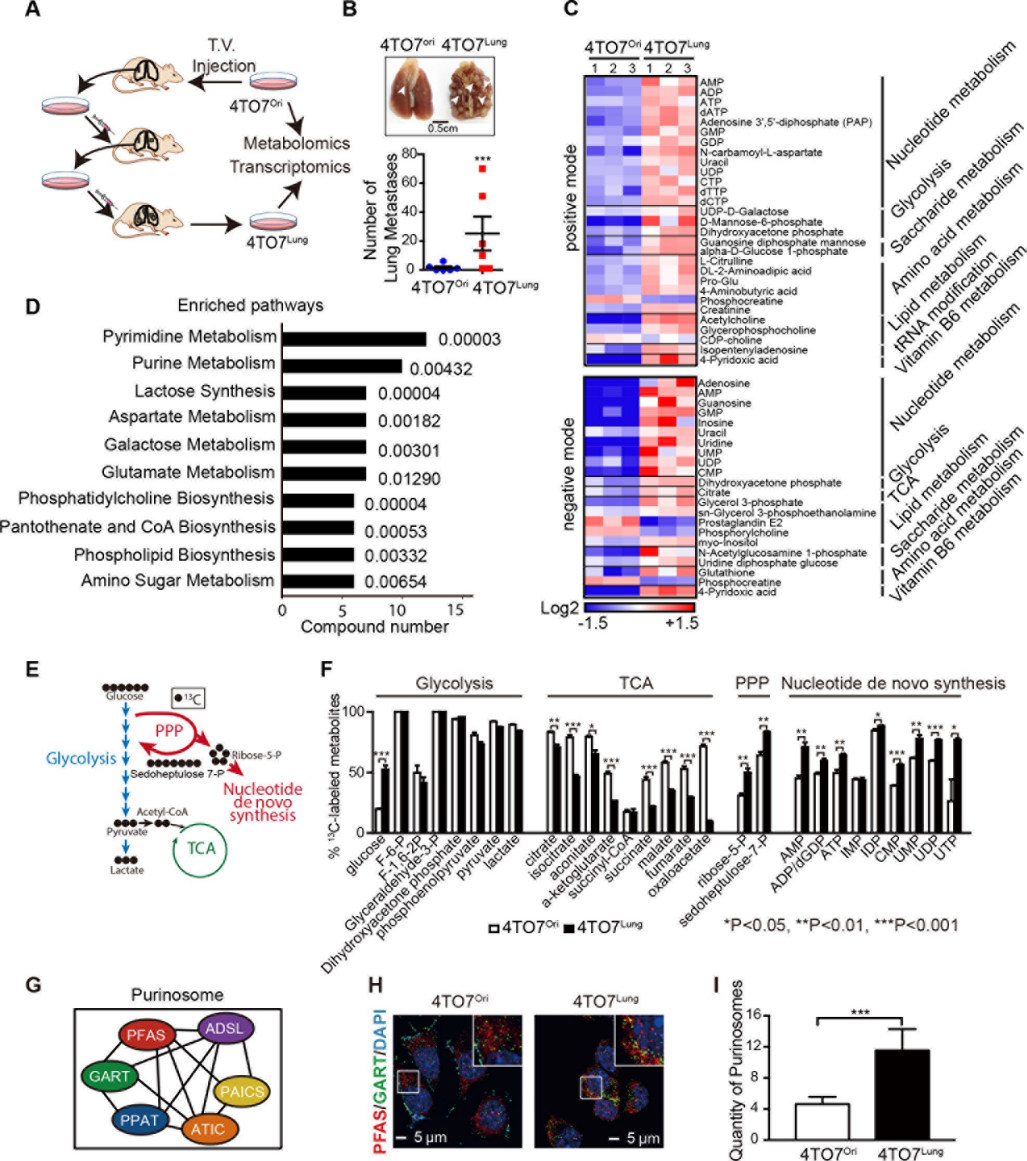
Metastatic breast cancer cells in lung display increased nucleotide de novo synthesis. **A**. Schematic diagram of the construction of breast cancer model and the screening of highly metastatic breast cancer cells. **B**. Representative photograph of lung metastatic foci after tail vein injection with breast cancer cells (top panel) and quantification of the number of lung metastases (bottom panel). (*n =* 6 mice in each group). **C**. Heatmap of the metabolites with a significant difference between 4TO7^Ori^ and 4TO7^Lung^ cells detected by positive and negative ion mode. **D**. MSEA of the differential metabolites in the 4TO7^Lung^ cells. The numbers on the right side represent the *P* value. **E**. Diagram of tracing glucose metabolism with U-[^13^C]-glucose in breast cancer cells. During glucose metabolism, increased ^13^C-labeled-glucose uptake does not affect the glycolysis as it mainly contributes to produce ribose-5-P for enhanced nucleotide de novo synthesis via enhanced PPP. **F**. The comparison of levels of different metabolites of glycolysis, TCA, PPP, and nucleotide de novo synthesis between 4TO7^Ori^ and 4TO7^Lung^ cells. Each value is an average of 3 replications. **G**. The composition of purinosome assembly in cells. Three enzymes in the de novo purine biosynthetic pathway (PPAT, GART, and FGAMS) first assembled to form the core of the purinosomes and interact with other enzymes. **H**. Immunofluorescence staining of enzymes in the de novo purine biosynthetic pathway, GART (green) and PFAS (red), form the core of the purinosomes (yellow) in 4TO7^Ori^ and 4TO7^Lung^. Scale bar represents 5 μm (*n =* 3 replications). **I**. Quantification of purinosomes in 4TO7^Ori^ and 4TO7^Lung^ in **H**. All values are presented as means ± SD. Statistical significance is shown as *P ≤ 0.05, **P ≤ 0.01, and ***P ≤ 0.001. The raw data used for quantification of **B**, **F**, and **I** can be found in S1 Data. ADSL, adenylosuccinate lyase; ATIC, inosine 5’-monophosphate cyclohydrolase; GART, phosphoribosylglycinamide synthase; MSEA, metabolite set enrichment analysis; PAICS, phosphoribosyl aminoimidazole succinocarboxamide synthetase; PFAS (FGAMS), phosphoribosyl formylglycinamidine synthase; PPAT, phosphoribosylpyrophosphate amidotransferase; PPP, pentose phosphate pathway; TCA, tricarboxylic acid cycle; T. V., tail vein injection.

To assess metabolic alterations during the metastasis of breast cancer cells, we isolated a subpopulation of murine breast cancer cells 4TO7 with enhanced lung metastatic behavior (denoted 4TO7^Lung^) from parental 4TO7 cells (denoted 4TO7^Ori^), which have much less metastatic capacity [15, 16] via 3-round selection in an in vivo experimental metastasis model in mice (Fig 1A and 1B). We conducted a metabolomics profiling of these cells followed by an isotope tracing of ^13^C after feeding the cells with ^13^C-labeled glucose. The results from metabolomics profiling showed that compared with 4TO7^Ori^, 4TO7^Lung^ cells displayed increased metabolites enriched in nucleotide metabolism, including all 5 nucleotides and the intermediate metabolites in their de novo synthesis pathways, such as N-carbamoyl-L-aspartate (Fig 1C and 1D). Moreover, isotope tracing of ^13^C confirmed that metastatic 4TO7^Lung^ cells increased the uptake of glucose, which was mainly utilized to synthesize nucleotides via the de novo pathway because enhanced glucose uptake went mainly to pentose phosphate pathway (PPP) for synthesizing ribose-5-phosphate and subsequent 5-phosphoribosyl-1-pyrophosphate (PRPP) for the de novo synthesis of nucleotides, while the glycolysis was not enhanced (Fig 1E and 1F). The purinosome is a multienzyme complex that is formed during the de novo purine synthesis [17, 18], which can be visualized by immunofluorescent staining of its core components (Fig 1G). Compared with 4TO7^Ori^ cells, 4TO7^Lung^ cells showed more purinosomes (Fig 1H and 1I), further confirming the enhanced purine de novo synthesis in high metastatic breast cancer cells.

### Nucleotide de novo synthesis genes are up-regulated in metastatic breast cancer cells and reversely correlate with patient’s survival

**Fig 2:**
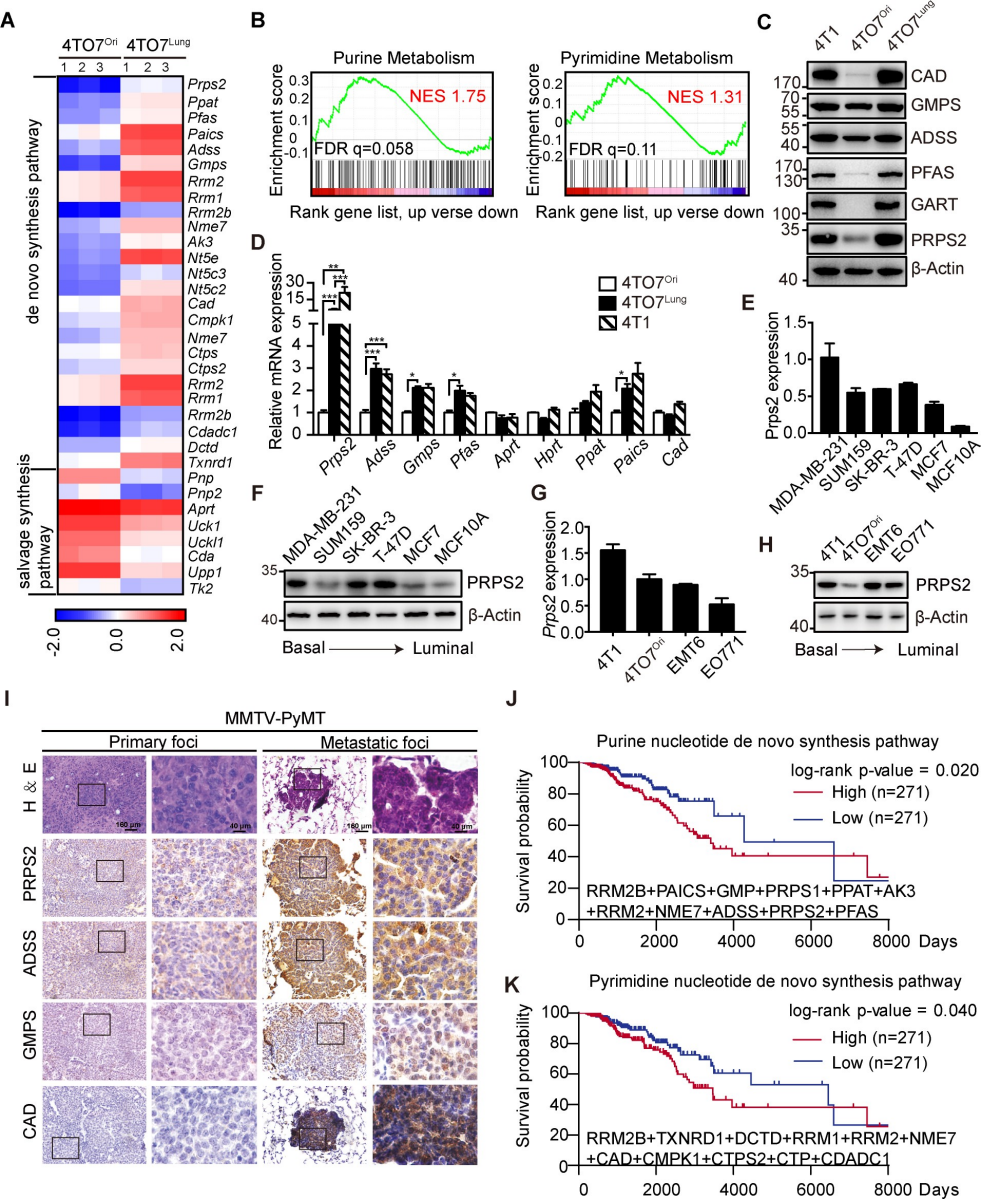
Nucleotide de novo synthesis genes are up-regulated in metastatic breast cancer cells and reversely correlate with patient’s survival. **A**. Heatmap of differentially expressed genes from RNA-seq data showed that 25 genes that regulated nucleotide de novo synthesis were up-regulated in the 4TO7^Lung^ cells and 8 genes that regulated salvage synthesis were down-regulated. **B**. GSEA of KEGG pathway showed that the genes up-regulated in the 4TO7^Lung^ cells are enriched in the purine and pyrimidine metabolism pathways. NESs and q values are shown. The raw data can be found in S1 Raw images. **C**. The protein expression levels of selected genes of de novo synthesis in 4T1, 4TO7^Ori^, and 4TO7^Lung^ cells. **D**. Relative mRNA levels of nucleotide de novo synthesis related genes in 4T1, 4TO7^Ori^, and 4TO7^Lung^ cells (*n =* 3 replications). **E**. Relative mRNA levels of Prps2 in a variety of human breast cancer cell lines (*n =* 3 replications). **F**. PRPS2 expression in a variety of human cell lines. **G**. Relative mRNA levels of *Prps2* in a variety of murine breast cancer cell lines (*n =* 3 replications). **H**. PRPS2 expression in a variety of murine cell lines. **I**. HE staining and IHC analyses of nucleotide de novo synthesis proteins in primary and metastatic foci of MMTV-PyMT mice. Scale bars, 160 μm for low magnification and 40 μm for high magnification. **J**. Kaplan–Meier survival analysis of breast cancer patients (*n =* 542) with different levels of genes in purine de novo synthesis pathway. Information of patients was acquired from TCGA database. **K**. Kaplan–Meier survival analysis of breast cancer patients (*n =* 542) with different levels of genes in pyrimidine de novo synthesis pathway. Information of patients was acquired from TCGA database. All values are presented as means ± SD. Statistical significance is shown as *P ≤ 0.05, **P ≤ 0.01, and ***P ≤ 0.001. The raw data used for quantification of **D**, **E**, **G**, **J**, and **K** can be found in S2 Data. ADSS, adenylosuccinate synthetase; CAD, carbamoyl-phosphate synthetase 2; FDR, false discovery rate; GART, phosphoribosylglycinamide synthase; GSEA, gene set enrichment analysis; GMPS, guanine monophosphate synthase; IHC, immunohistochemistry; KEGG, Kyoto Encyclopedia of Genes and Genomes; MMTV-PyMT, mouse mammary tumor virus-polyoma middle tumor-antigen; NES, normalized enrichment score; PFAS, phosphoribosyl formylglycinamidine synthase; PRPS2, phosphoribosylpyrophosphate synthetase 2; TCGA, The Cancer Genome Atlas.

To gain insights into the metastasis-related metabolic reprogramming from gene transcriptional level, we analyzed the transcriptome of 4TO7^Lung^ cells. Gene set enrichment assay (GSEA) revealed that genes encoding key enzymes in nucleotide de novo synthesis such as *Prps2*, *Ppat*, *Pfas Gmps*, *Gart*, etc were significantly up-regulated, whereas genes involved in catalyzing nucleotide salvage synthesis pathway were down-regulated (Fig 2A and 2B; S1A and S1B Fig). These results were further verified by western bolt and real-time quantitative polymerase chain reaction (qRT-PCR) (Fig 2C and 2D). Consistently, this gene expression signature was also observed in another highly metastatic, 4TO7-originated murine breast cancer cell line 4T1 (Fig 2C and 2D) [16]. Moreover, the expression level of the rate-limiting enzyme PRPS2, which catalyze the PRPP formation, positively correlated with the malignancy of murine and human breast cancer cell lines (Fig 2E–2H).

Next, we performed immunohistochemistry analyses of key enzymes in nucleotide de novo synthesis reactions, i.e., carbamoyl-phosphate synthetase 2 (CAD), phosphoribosylpyrophosphate synthetase 2 (PRPS2), guanine monophosphate synthase (GMPS), and adenylosuccinate synthetase (ADSS), in primary and metastatic tumor foci spontaneously formed in mouse mammary tumor virus-polyoma middle tumor-antigen (MMTV-PyMT) mice or formed in 4T1-inoculated syngeneic mouse model, confirming that these enzymes were up-regulated in metastatic cancer cells (Fig 2I, S1C Fig).

Additionally, we analyzed a cohort containing 542 breast cancer patients from The Cancer Genome Atlas (TCGA) database and found the expression levels of major genes in purine de novo synthesis pathways, when considered together, reversely correlates with the survival of patients (Fig 2J), as did the pyrimidine synthesis genes (Fig 2K). Analyses of breast cancer patients from Kaplan-Meier Plotter (https://kmplot.com/analysis/index.php?p=service&cancer=breast) also suggested that high expression of individual key enzymes, such as PRPS2, CAD, GMPS, and phosphoribosylaminoimidazole carboxylase and phosphoribosylaminoimidazolesuccinocarboxamide synthase (PAICS), correlated with poor patients’ survival (S1D–S1G Fig).

### Blocking nucleotide de novo synthesis by silencing PRPS2 inhibits lung metastasis of breast cancer cells

**Fig 3:**
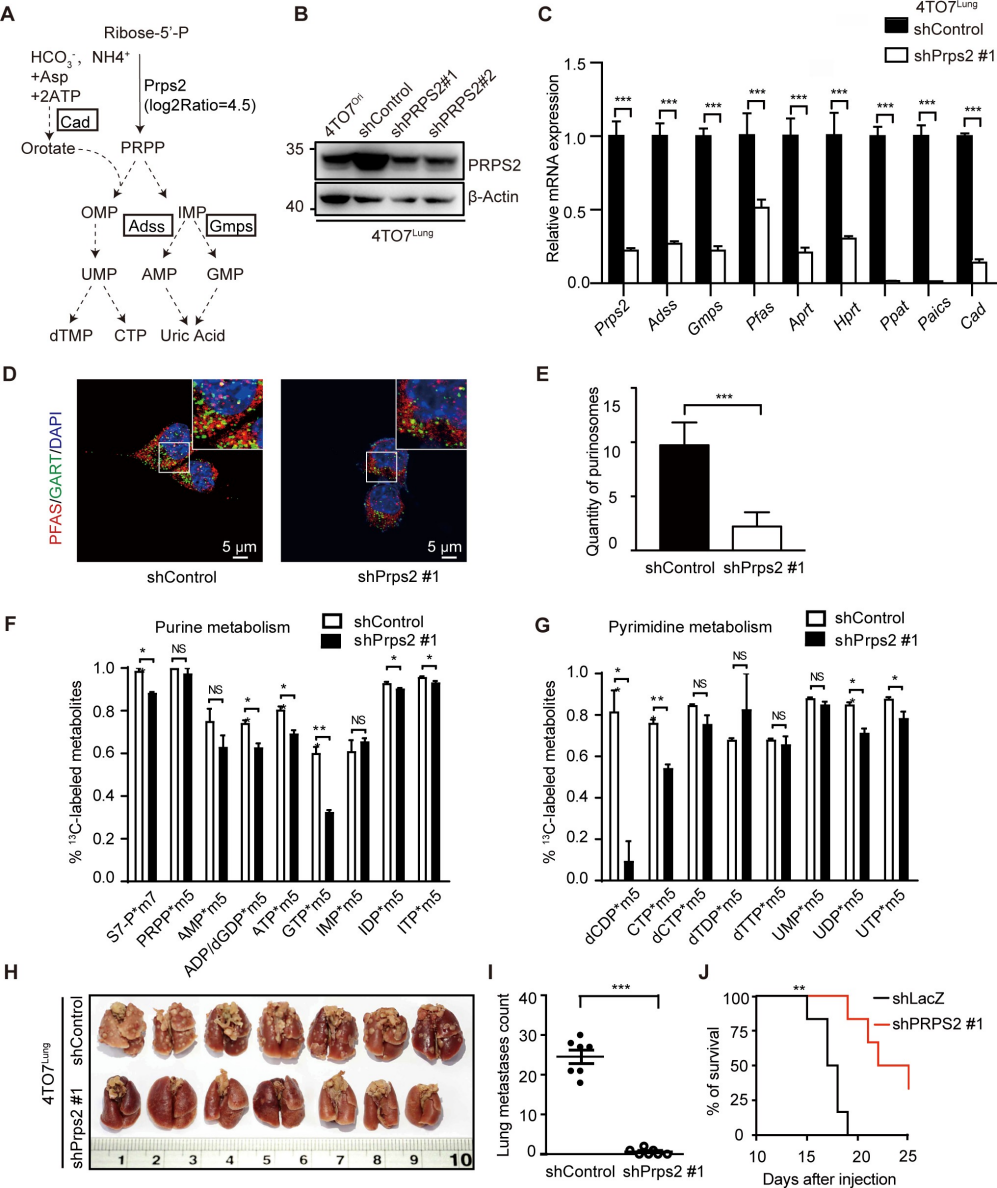
Blocking nucleotide de novo synthesis by silencing PRPS2 inhibits lung metastasis of breast cancer cells. **A**. Schematic diagram of nucleotide de novo synthesis process. **B**. PRPS2 expression levels in 4TO7^Ori^, shControl-4TO7^Lung^, and shPrps2-4TO7^Lung^ cells. The 4TO7^Lung^ cells infected with lentiviruses expressing shRNAs targeting *Prps2* (shPrps2#1 and shPrps2#2) or targeting *LacZ* (shControl) as control. **C**. Relative mRNA levels of nucleotide de novo synthesis related genes determined by qRT-PCR. Results were normalized to *β-Actin* and to shControl (*n =* 3 replications). **D**. Immunofluorescence staining of enzymes in the de novo purine biosynthetic pathway, GART (green) and PFAS (red), form the core of the purinosomes (yellow) in shControl-4TO7^Lung^ and shPrps2#1-4TO7^Lung^ cells (*n =* 3 replications). **E**. Quantification of purinosomes in shControl-4TO7^Lung^ and shPrps2#1-4TO7^Lung^ cells in **D**. The colocalization (yellow) of GART and PFAS represents purinosomes assembly. **F-G**. The levels of the metabolites with seven ^13^C isotopologues (m^7^) (S7-P*m^7^) or five ^13^C isotopologues (m^5^) of purine (**F**) and pyrimidine (**G**) synthesis in shControl-4TO7^Lung^ and shPrps2#1-4TO7^Lung^ cells. Each value is an average of 3 replications. **H**. Image of lung metastases of breast cancer at 20 days after tail vein injection with shControl-4TO7^Lung^ and shPrps2#1-4TO7^Lung^ cells in mice. (*n =* 7 in each group). **I**. Quantification of lung metastases in **H**. **J**. Kaplan–Meier survival analysis of mice (*n =* 6) with tail vein injection of shControl-4TO7^Lung^ or shPrps2#1-4TO7^Lung^ cells. All values are presented as means ± SD. Statistical significance is shown as *P ≤ 0.05, **P ≤ 0.01, and ***P ≤ 0.001. The raw data used for quantification of **C**, **E**, **F**, **G**, **I**, and **J** can be found in S3 Data. Adss, adenylosuccinate synthetase; AMP, adenosine monophosphate; Cad, carbamoyl-phosphate synthetase 2; CTP, cytidine triphosphate; dTMP, deoxythymidine monophosphate; GART, phosphoribosylglycinamide synthase; GMP, guanosine monophosphate; Gmps, guanine monophosphate synthase; IHC, immunohistochemistry; IMP, inosine monophosphate; OMP, orotidine 5'-monophosphate; PFAS, phosphoribosyl formylglycinamidine synthase; PRPP, 5-phosphoribosyl-1-pyrophosphate; PRPS2, phosphoribosylpyrophosphate synthetase 2; qRT-PCR, real-time quantitative polymerase chain reaction; UMP, uridine monophosphate.

To further assess the significance of nucleotide de novo synthesis for breast cancer metastasis, we specifically silenced *Prps2*, a key enzyme catalyzing the first reaction in nucleotide synthesis, which was highly up-regulated in metastatic cells [19] (Fig 3A and 3B). Knocking down *Prps2* caused dramatically decreased expression of other key enzymes in de novo purine and pyrimidine synthesis pathways (Fig 3C; S2A and S2B Fig), suggesting that *Prps2* might cooperate with these enzymes to regulate the whole de novo synthesis process. In *Prps2*-silenced 4TO7^Lung^ cells, we observed less formation of purinosomes (Fig 3D and 3E). Isotope tracing of ^13^C further confirmed that silencing *Prps2* successfully decreased the de novo synthesis of nucleotides in 4TO7^Lung^ cells (Fig 3F and 3G).

Next, we assessed the impact of reduced de novo synthesis on breast cancer metastasis in vivo by injecting *Prps2*-silenced 4TO7^Lung^ cells or 4T1 cells into the tail vein. We observed that reduction of de novo nucleotide synthesis in these highly metastatic cells dramatically reduced their lung metastasis behavior, resulting in much better survival of cancer-bearing mice (Fig 3H–3J, S2C Fig). Furthermore, in a syngeneic mouse breast cancer model using subcutaneously inoculated 4T1 cells, silencing *Prps2* dramatically reduced lung metastasis (S2D and S2E Fig), whereas the growth of the primary tumor was not significantly affected (S2F Fig). These results suggested that *Prps2*-mediated de novo nucleotide synthesis was essential for the lung metastatic capacity of breast cancer cells.

### De novo nucleotide synthesis enhances the stemness of breast cancer cells

**Fig 4:**
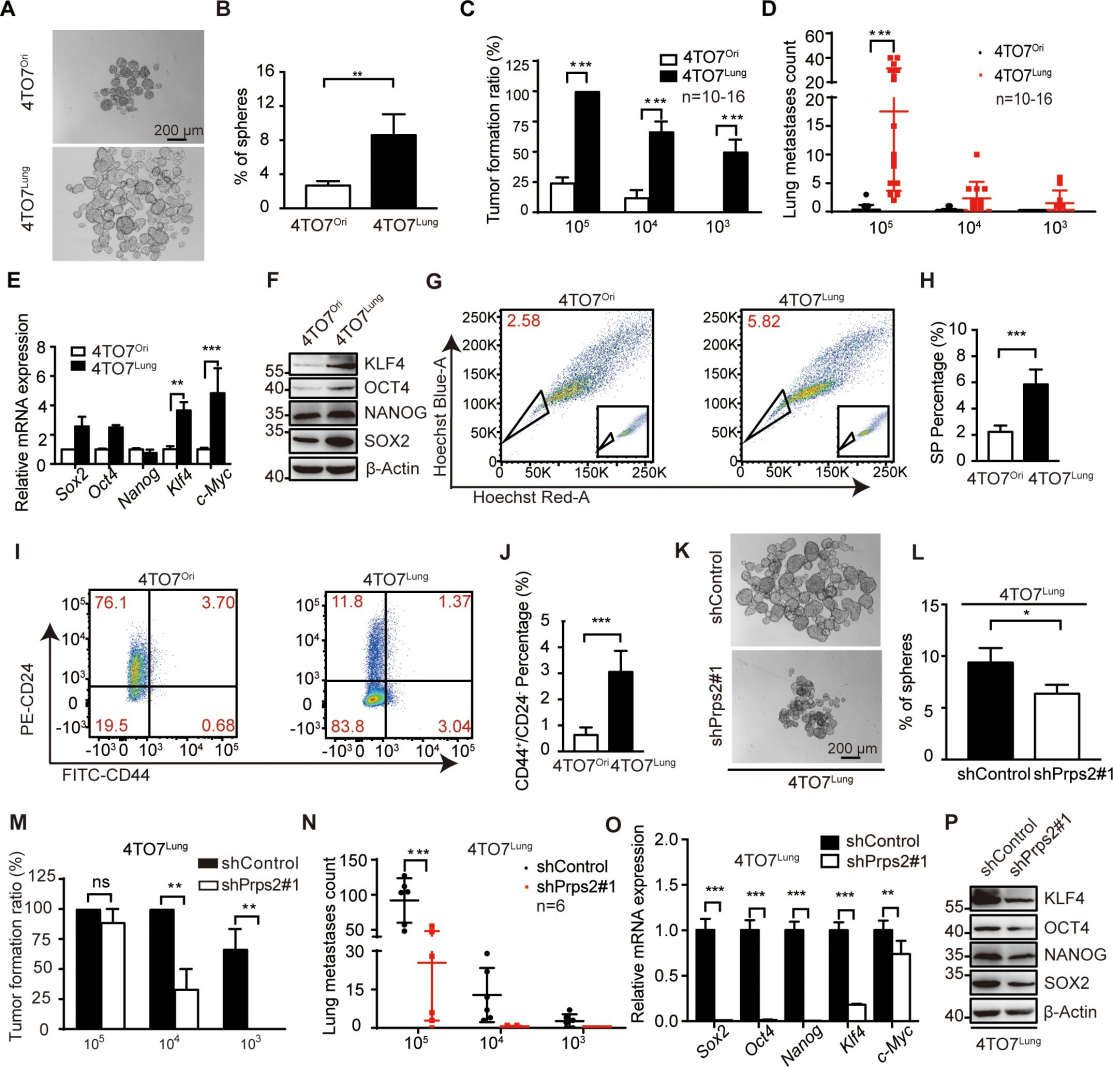
De novo nucleotide synthesis enhances the stemness of breast cancer cells. **A**. Sphere formation assay of 4TO7^Ori^ and 4TO7^Lung^ cells in normal sphere medium (*n =* 3 replications). **B**. Quantification of spheres per well in 4TO7^Ori^ and 4TO7^Lung^ in **A**. **C**. Tumor formation ratio of mice after tail vein injection with 4TO7^Ori^ and 4TO7^Lung^ cells in different gradients. Mice of 10^5^ (*n =* 16), 10^4^ (*n =* 12–16), and 10^3^ (*n =* 10) groups were observed for 14 days, 40 days, and 100 days, respectively. **D**. Quantification of lung metastatic foci of mice after tail vein injection with 4TO7^Ori^ and 4TO7^Lung^ cells in different gradients. **E**. Relative mRNA levels of stemness-related genes in 4TO7^Ori^ and 4TO7^Lung^ (*n =* 3 replications). **F**. The protein expression levels of stemness-related genes in 4TO7^Ori^ and 4TO7^Lung^ cells. **G**. Side population analyzed by FACS in 4TO7^Ori^ and 4TO7^Lung^ cells (*n =* 3 replications). The raw data can be found in S2 raw images. **H**. Quantification of FACS analyses in **G**. **I**. Identification of breast CSCs (CD44^high^/CD24^low^) in 4TO7^Ori^ and 4TO7^Lung^ cells by flow cytometry (*n =* 3 replications). **J**. Quantification of FACS analysis in **I**. **K**. Sphere formation assay of shControl-4TO7^Lung^ and shPrps2#1-4TO7^Lung^ cells in normal sphere medium (*n =* 3 replications). **L**. Quantification of spheres per well in shControl-4TO7^Lung^ and shPrps2#1-4TO7^Lung^ cells in **K**. **M**. Tumor formation ratio of mice after tail vein injection with shControl-4TO7^Lung^ or shPrps2#1-4TO7^Lung^ cells in different gradients. Mice of 10^5^ (*n =* 6), 10^4^ (*n =* 6), and 10^3^ (*n =* 6) groups were observed for 14 days, 40 days, and 100 days, respectively. **N**. Quantification of lung metastatic foci of mice after tail vein injection with shControl-4TO7^Lung^ or shPrps2#1-4TO7^Lung^ cells in different gradients in **M**. **O**. Relative mRNA levels of stemness-related genes in shControl-4TO7^Lung^ and shPrps2#1-4TO7^Lung^ cells (*n =* 3 replications). **P**. The protein expression levels of stemness-related genes in shControl-4TO7^Lung^ and shPrps2#1-4TO7^Lung^ cells. All values are presented as means ± SD. Statistical significance is shown as *P ≤ 0.05, **P ≤ 0.01, and ***P ≤ 0.001. The raw data used for quantification of **B**, **C**, **D**, **E**, **H**, **J**, **L**, **M**, **N**, and **O** can be found in S4 Data. CSC, cancer stem cell; FACS, fluorescence-activated cell sorting.

To further study the cellular mechanism underlying de novo nucleotide synthesis-enhanced metastatic capacity of breast cancer, we firstly investigated the apoptosis resistance, epithelial to mesenchymal transition (EMT), motility, and proliferation of 4TO7^Ori^ and 4TO7^Lung^ cells. We found no significant differences of those capacity except cell motility (S3A–S3F Fig). Interestingly, the motility of 4TO7^Lung^ cells was even attenuated, shown by the transwell migration assay, indicating enhanced metastasis of 4TO7^Lung^ cells was not due to the change of motility. We next tested the stemness of 4TO7^Ori^ and 4TO7^Lung^ cells by sphere formation assay. The 4TO7^Lung^ cells showed higher sphere formation efficiency than 4TO7^Ori^ cells (Fig 4A and 4B), suggesting 4TO7^Lung^ cells gained more stem-cell-like properties. To further confirm, we performed a limited dilution allograft assay by injecting 4TO7^Lung^ or 4TO7^Ori^ cells into mice through tail vein. The 4TO7^Lung^ cells showed a much stronger tumor formation capacity in the lung even when the cell number was as low as 10^3^ cells/injection (Fig 4C and 4D). Furthermore, 4TO7^Lung^ cells showed higher expression of the signature genes of cancer stem cells (CSCs), suggesting the enhanced stemness of 4TO7^Lung^ cells (Fig 4E and 4F). The flow cytometry assay also revealed higher percentage of side population cells in 4TO7^Lung^ cells in comparison with 4TO7^Ori^ cells (Fig 4G and 4H). By using another well-established breast CSC signature, i.e., CD44 and CD24, we found that 4TO7^Lung^ cells had significantly increased percentage of CSC-like CD44^high^/CD24^low^ populations in comparison with 4TO7^Ori^ (Fig 4I and 4J). All this evidence suggested the enhanced stemness of 4TO7^Lung^ cells contributed to the high lung metastasis behavior.

To confirm the enhanced stemness of 4TO7^Lung^ cells was due to Prps2-mediated de novo nucleotide synthesis, we silenced *Prps2* in 4TO7^Lung^ or 4T1 cells. We found the sphere formation capacities of *Prps2*-silenced 4TO7^Lung^ or 4T1 cells were greatly attenuated (Fig 4K and 4L; S4A-S4D Fig). Moreover, a limited dilution allograft assay using different dosages of 4TO7^Lung^ cells showed that the tumor initiation capacities in the lung was significantly reduced once *Prps2* was knocked down (Fig 4M and 4N). In addition, silencing *Prps2* dramatically reduced the expression of stemness signature genes in 4TO7^Lung^ cells (Fig 4O and 4P, S4E and S4F Fig), further confirming the pivotal role of *Prps2*-mediated de novo nucleotide synthesis in the enhancement of CSC-like properties in breast cancer cells.

### De novo nucleotide synthesis promotes breast cancer cell stemness via cyclic guanosine monophosphate/PKG pathway

**Fig 5:**
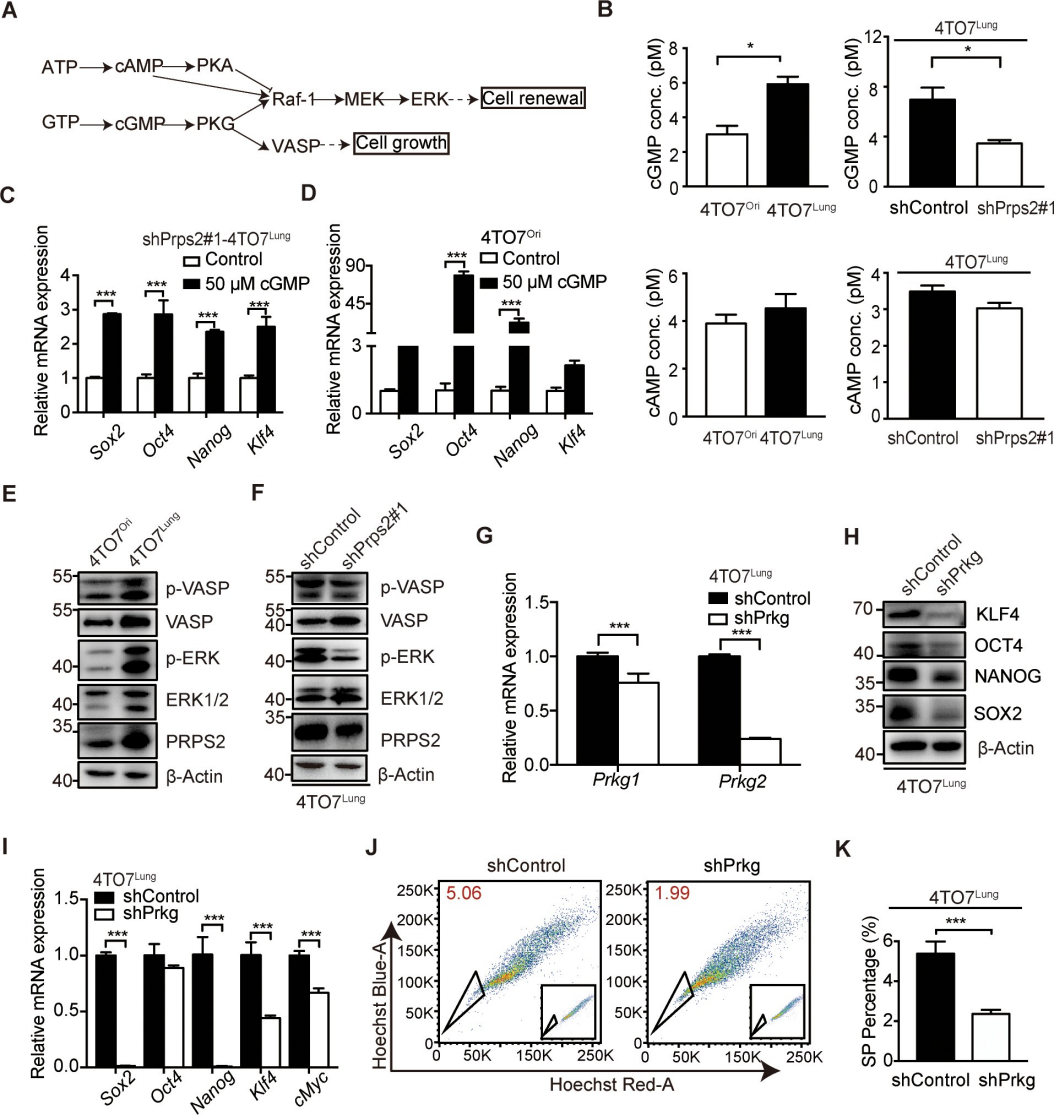
De novo nucleotide synthesis promotes breast cancer cell stemness via cGMP/PKG pathway. **A**. Schematic diagram of cAMP/PKA and cGMP/PKG signaling pathway. **B**. cGMP and cAMP levels in 4TO7^Ori^, 4TO7^Lung^ cells, shControl-4TO7^Lung^ and shPrps2#1-4TO7^Lung^ cells by ELISA analysis (*n =* 3 replications). **C**. Relative mRNA levels of stemness-related genes in shPrps2#1-4TO7^Lung^ after cGMP analog stimulation for 48 hours (*n =* 3 replications). **D**. Relative mRNA levels of stemness-related genes in 4TO7^Ori^ after cGMP analog stimulation (*n =* 3 replications). **E**. Phosphorylation level of VASP (Ser239) and ERK1/2 (Thr202 and Tyr204) in PKG-MAPK signaling pathway in 4TO7^Ori^ and 4TO7^Lung^ cells under the normal culture condition. **F**. Phosphorylation level of VASP (Ser239) and ERK1/2 (Thr202 and Tyr204) in PKG-MAPK signaling pathway in shControl-4TO7^Lung^ and shPrps2#1-4TO7^Lung^ cells under the normal culture condition. **G**. qRT-PCR analysis of *Prkg1* and *Prkg2* to confirm the knockdown efficiency in shPrkg-4TO7^Lung^ cells. The 4TO7^Lung^ cells infected with lentiviruses expressing shRNAs targeting *Prkg1* and *Prkg2* (shPrkg) and targeting *LacZ* (shControl) as control (*n =* 3 replications). **H**. The protein expression levels of stemness-related genes in shControl-4TO7^Lung^ and shPrkg-4TO7^Lung^ cells. **I**. Relative mRNA levels of stemness-related genes determined by qRT-PCR in shControl-4TO7^Lung^ and shPrkg-4TO7^Lung^ cells (*n =* 3 replications). **J**. Side population analyzed by FACS in shControl-4TO7^Lung^ and shPrkg-4TO7^Lung^ cells (*n =* 2 replications). The raw data can be found in S2 raw images. **K**. Quantification of FACS analysis in **H**. All values are presented as means ± SD. Statistical significance is shown as *P ≤ 0.05, **P ≤ 0.01, and ***P ≤ 0.001. The raw data used for quantification of **B**, **C**, **D**, **G**, **I**, and **K** can be found in S5 Data. cAMP, cyclic guanosine monophosphate; cGMP, cyclic adenosine monophosphate; FACS, fluorescence-activated cell sorting; qRT-PCR, real-time quantitative polymerase chain reaction; SP, side population.

To gain insights into the molecular mechanism by which de novo nucleotide synthesis enhanced the stemness of breast cancer cells, we were focusing on 2 nucleotide-related signaling metabolites, i.e., cyclic guanosine monophosphate (cGMP) and cyclic adenosine monophosphate (cAMP), which are able to activate downstream signaling pathway to regulate cell growth and renewal (Fig 5A) [20]. Thus, we compared the cGMP and cAMP levels and found that high metastatic 4TO7^Lung^ cells showed little change on cAMP level, while significantly increasing on cGMP level, which could be decreased by silencing *Prps2* (Fig 5B). Silencing *Prps2* attenuated stemness of 4TO7^Lung^ cells could be rescued by adding cGMP analog in the medium, strongly suggesting that cGMP was a key downstream factor mediating *Prps2*-boosted stemness (Fig 5C). cGMP could also increase the CSC-like properties of low metastatic 4TO7^Ori^ cells, further demonstrating a key role of cGMP-dependent pathway in supporting CSC-like properties (Fig 5D). Next, we examined the activation of cGMP-dependent kinase PKG and downstream pathways. We observed highly activated PKG in 4TO7^Lung^ cells shown by the phosphorylation of its downstream factors extracellular signal-regulated kinase (ERK) and vasodilator-stimulated phosphoprotein (VASP) (Fig 5E), which could be inactivated by knocking down *Prps2* (Fig 5F), suggesting *Prps2*-enhanced nucleotide synthesis was able to activate cGMP/PKG pathway. We also found that silencing both *Prkg1* and *Prkg2*, 2 members of PKG, dramatically reduced the CSC-like properties of 4TO7^Lung^ cells, shown by reduced expression of stemness signature genes (Fig 5G–5I) and the side populations in 4TO7^Lung^ cells (Fig 5J and 5K), confirming the importance of cGMP/PKG pathway in regulating the stemness of breast cancer cells.

### cGMP/PKG-MEK-ERK signaling pathway enhances breast cancer cell stemness

**Fig 6:**
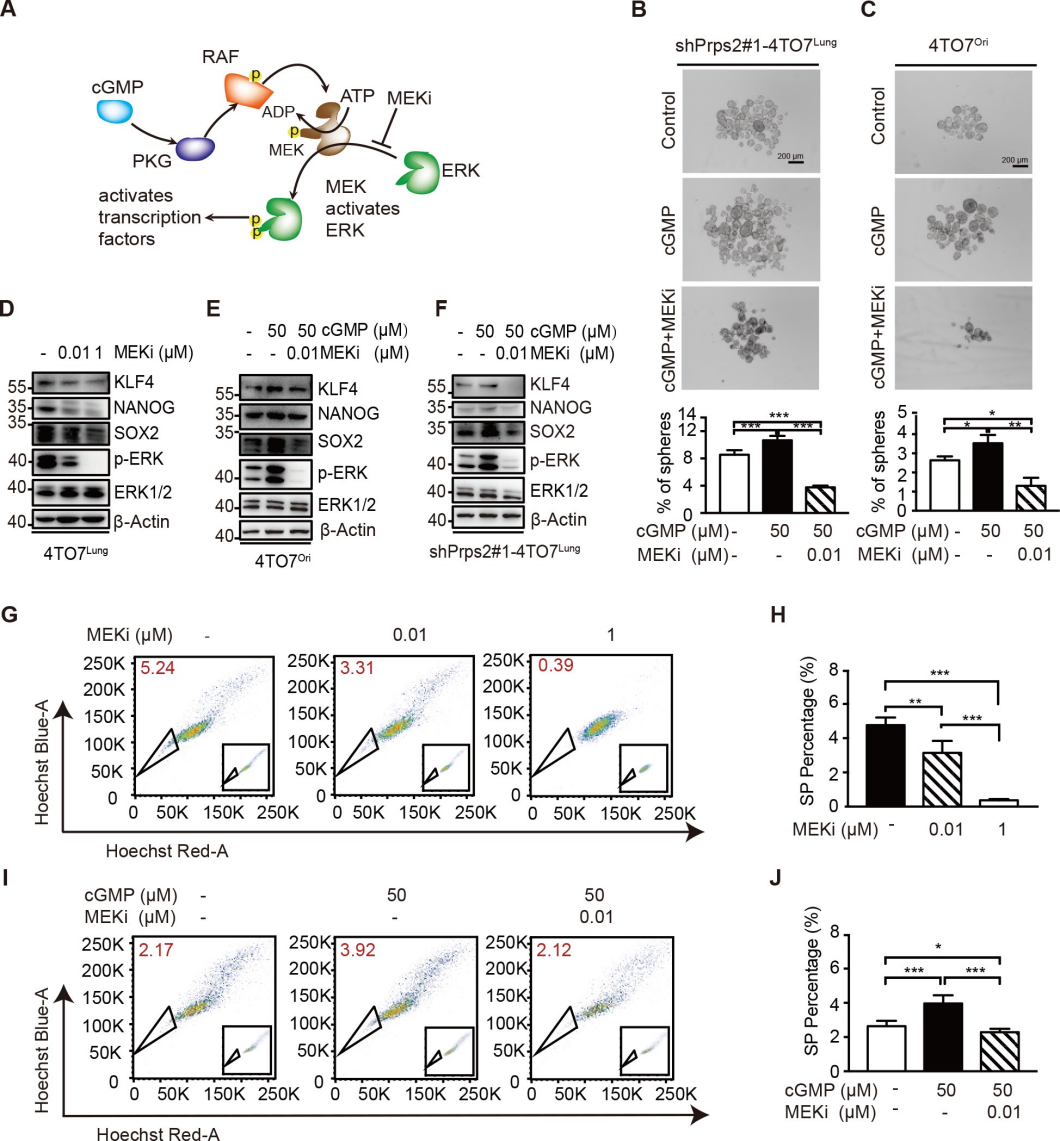
cGMP/PKG-MEK-ERK signaling pathway enhances breast cancer cell stemness. **A**. Schematic diagram of cGMP-PKG-MAPK signaling pathway. **B**. Photographs and quantification of spheres per well of shPrps2#1-4TO7^Lung^ in normal sphere medium with/without cGMP analog or MEKi treatment (*n =* 3 replications). **C**. Photographs and quantification of spheres per well of 4TO7^Ori^ in normal sphere medium with/without cGMP analog or MEKi treatment (*n =* 3 replications). **D**. The protein expression levels of stemness-related genes in 4TO7^Lung^ cells with/without MEKi treatment for 24 hours. **E**. The protein expression levels of stemness-related genes in 4TO7^Ori^ cells with/without cGMP analog or MEKi treatment for 72 hours. **F**. The protein expression levels of stemness-related genes in shPrps2#1-4TO7^Lung^ cells with/without cGMP analog or MEKi treatment for 72 hours. **G**. Side population analyzed by FACS in 4TO7^Lung^ cells with/without MEKi treatment (*n =* 2 replications). The raw data can be found in S2 raw images. **H**. Quantification of FACS analyses in **G**. **I**. Side population analyzed by FACS in 4TO7^Ori^ cells with/without cGMP analog or MEKi treatment (*n =* 2 replications). The raw data can be found in S2 raw images. **J**. Quantification of FACS analyzed in **I**. All values are presented as means ± SD. Statistical significance is shown as *P ≤ 0.05, **P ≤ 0.01, and ***P ≤ 0.001. The raw data used for quantification of **B**, **C**, **H**, and **J** can be found in S6 Data. cGMP, cyclic guanosine monophosphate; ERK, extracellular signal-regulated kinase; FACS, fluorescence-activated cell sorting; MEK, mitogen-activated protein kinase kinase; MEKi, MEK inhibitor; PKG, cGMP-dependent protein kinase; RAF, rapidly accelerated fibrosarcoma.

We next study the cGMP/PKG downstream pathway regulating the stemness of breast cancer cells. We were focusing on the downstream MEK-ERK signaling pathway because it has been reported can regulate cell renewal (Fig 6A) [21]. The cGMP analog was able to rescue the sphere formation capacity of *Prps2*-silenced 4TO7^Lung^ cells, which could be further inhibited by the MEK inhibitor (MEKi) (Fig 6B). In 4TO7^Ori^ cells, cGMP analog-enhanced sphere formation capacity could be also blocked by MEKi (Fig 6C).

Besides, MEKi could inhibit not only the expression of stemness signature genes in highly metastatic 4TO7^Lung^ cells (Fig 6D) but also cGMP-boosted stemness genes in low metastatic 4TO7^Ori^ and *Prps2*-silenced 4TO7^Lung^ cells (Fig 6E and 6F, S4G Fig). In addition, MEKi greatly reduced the side population of 4TO7^Lung^ cells in a dose-dependent manner (Fig 6G and 6H) and blocked the cGMP-induced side population of 4TO7^Ori^ cells (Fig 6I and 6J). Together, these data supported that cGMP/PKG activated downstream MEK-ERK signaling pathway to increase the CSC-like properties of breast cancer cells to sustain their metastasis.

### Increased UA level correlates with cancer progression in breast cancer patients

**Fig 7:**
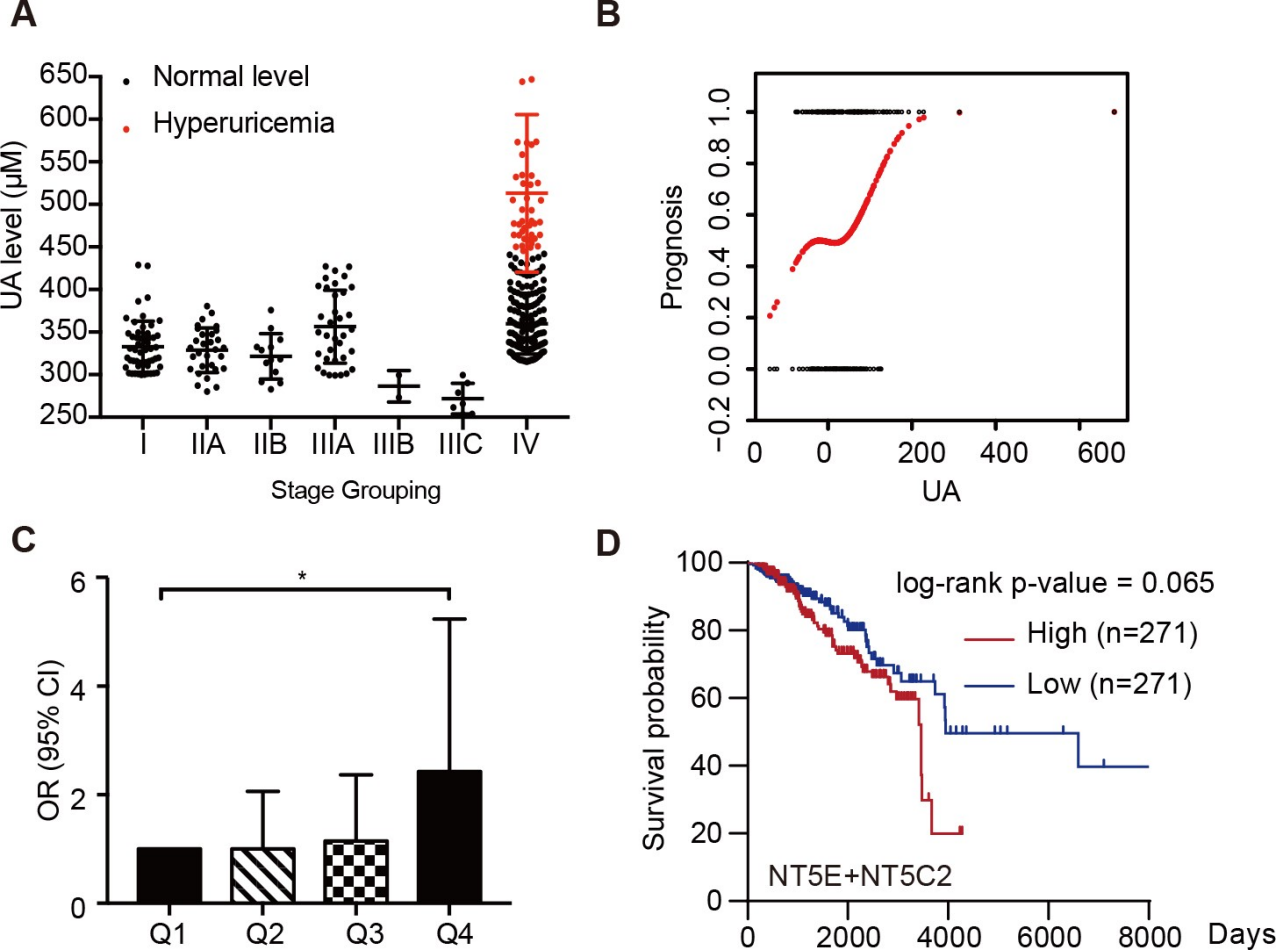
Increased UA level correlates with cancer progression in breast cancer patients. **A**. UA level of breast cancer patients (*n =* 337) at the first hospitalized point from the Department of Oncology, Chinese PLA General Hospital, Beijing, China. **B**. Linear correlation analysis of the relationship between the prognosis and the change of UA level before and after treatment (*n =* 236). The abscissa shows the change of UA level before and after treatment, and the ordinate shows the patients’ conditions defined 0 as improvement and 1 as deterioration. **C**. OR of each quartile of numerical change in UA compared with participants in the lowest part (*n =* 236). **D**. Kaplan–Meier survival analysis of breast cancer patients with different NT5E and NT5C2 expression levels. Information of patients was acquired from TCGA database (*n =* 542). The raw data used for quantification of **A**, **B**, **C** and **D** can be found in S7 Data. OR, odds ratio; TCGA, The Cancer Genome Atlas; UA, uric acid.

Last but not least, we evaluated the UA levels in patients with breast cancer, because as a downstream metabolite of purine metabolism, its level always fluctuates with nucleotide de novo synthesis activity. As expected, we found that in 337 breast cancer patients, UA level is highly elevated in stage IV patients who have distant metastases (Fig 7A). The linear correlation analysis also revealed that the plasma UA level is correlated with patients’ poor prognosis (Fig 7B). We found that every 1-μM increase in plasma UA after clinical intervention was associated with a 0.8% increase in the risk of rapid poor prognosis (Table 1), and when compared patients with different level of UA changes, the risk of poor prognosis in the highest quartile was 2.427 (95% CI 1.126, 5.235, *P =* 0.02371), which means that the more UA increased after clinical intervention, the greater risk of poor prognosis in patients (Fig 7C, Table 2). In addition, based on the analysis of TCGA database, we found that the high expression of genes involved in UA production roughly correlates with poor survival (Fig 7D). These data suggested that as a downstream metabolite of purine metabolism, UA level has the potential to be utilized to evaluate the prognosis of breast cancer patients.

**Table 1:** The association between the change of UA and prognosis.

	Prognosis	
	OR (95% CI)	*P* value
Numerical change of UA	1.008 (1.003, 1.012)	0.00149

OR, odds ratio; UA, uric acid.

**Table 2:** The association between the change of UA and the risk of deterioration in breast cancer patients.

Numerical change of UA	Prognosis	
	OR (95% CI)	*P* value
Q1 (−140, −6.9)	1.0	
Q2 (−6.9, 28.75)	1.000 (0.485, 2.060)	1.00000
Q3 (28.75, 71.95)	1.146 (0.555, 2.366)	0.71177
Q4 (71.95, 681.7)	2.427 (1.126, 5.235)	0.02371

*ORs of all groups were compared with group Q1.

OR, odds ratio; Q, quartile; UA, uric acid.

**Fig 8:**
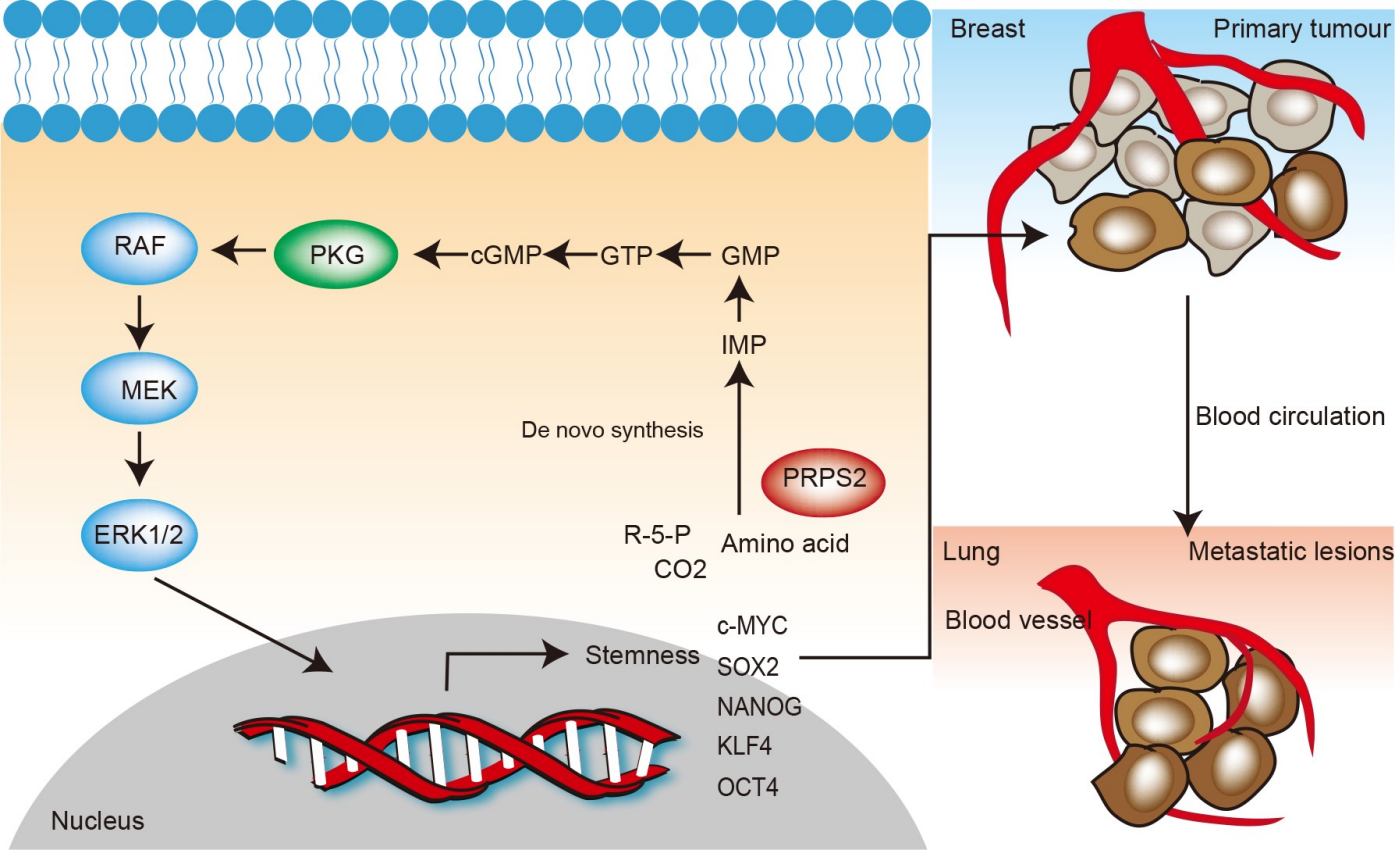
Schematic representation of nucleotide de novo synthesis regulated by PRPS2 increases breast cancer cell stemness and metastasis via cGMP-PKG-MAPK signaling pathway. In breast cancer cells, where metabolism reprogramming happens, oncogenes can up-regulate nucleotide de novo synthesis which produce a series of metabolites. A key metabolite of de novo synthesis, GTP, is able to generate cGMP to promote cGMP-dependent protein kinases, PKG to activate MAPK pathway, resulting in the increased stem cell signature gene expression and breast cancer lung metastasis. cGMP, cyclic guanosine monophosphate; ERK, extracellular signal-regulated kinase; GTP, guanosine-5'-triphosphate; MEK, mitogen-activated protein kinase kinase; PKG, cGMP-dependent protein kinase; PRPS2, phosphoribosylpyrophosphate synthetase 2; RAF, rapidly accelerated fibrosarcoma.

All together, our study illustrated a new metabolic feature during the lung metastasis of breast cancer, i.e., enhanced nucleotide de novo synthesis regulated by PRPS2 leads to more cGMP formation, consequently activating cGMP-dependent PKG and downstream MEK-ERK signaling pathway to increase the stemness of breast cancer cells and the metastasis (Fig 8).

## Discussion

### The role of de novo synthesis in lung metastasis of breast cancer

We established a breast cancer metastasis model by tail vein injecting with less-metastatic cancer cells 4TO7 to screen high-metastatic cells from lung metastatic foci in mice. This model imitated circulating breast cancer cells relocalized in distant organs and colonized to form new metastatic foci. With metabolomics analysis, we found that high metastatic breast cancer cells showed increases in many metabolisms, including nucleotide metabolism, glycolysis, amino acid metabolism, and lipid metabolism. During the development of breast cancer, tumor cells need certain amounts of nucleotides. Increased de novo synthesis can provide more nucleotides to support tumor development. However, the mechanism of the activation of de novo synthesis is still not clear, even though it has been reported that PRPS2 translation can be regulated by the mammalian target of rapamycin (mTOR) signaling pathway [17, 22, 23].

### The role of cGMP in lung metastasis of breast cancer

It has been noticed that deregulation of cGMP signaling is an important step in the tumorigenesis process in at least a subset of breast cancers [24–27]. Our findings pointed that cGMP, likely as a consequence of the increased de novo synthesis, can promote cGMP-dependent protein kinase PKG to active downstream pathways [20, 28]. More importantly, as cGMP signaling is a common signaling pathway in all types of breast cancer and largely independent of the estrogen receptor, progesterone receptor or HER-2 receptor, targeting this pathway is a promising and novel approach to improve triple-negative breast cancer survival rates.

### The role of PRPS2 in lung metastasis of breast cancer

There are 2 PRPS isoforms in mice, PRPS1 and PRPS2. PRPS2 shares about 95% amino acid identity with the PRPS1 isoform [19]. In our transcriptomics profiling, PRPS2 showed large variation, which suggested its exclusive role in regulating de novo synthesis of breast cancer cells. This may due to its high sensitivity to fluctuation of its substrate, ATP, whose levels are elevated in many tumor cells [29]. Nontargeted inhibitors of nucleotide biosynthesis have been utilized as chemotherapeutic drugs for many years [30, 31]. However, these inhibitors produce severe toxicities in normal tissues. Our findings found that PRPS2 can regulate nucleotide de novo synthesis to change breast cancer cell stem–like properties in the metastasis, strongly suggesting PRPS2 can be a new specific therapeutic target for breast cancer treatment.

### The role of increased UA in breast cancer metastasis

The UA is not only a metabolite of enhanced purine metabolism but also may function as a regulator of cancer cells. It has been reported that UA provides an antioxidant defense in humans [32]. As the lungs are exposed daily to oxidants generated either endogenously or exogenously [33], increased UA levels in breast cancer cells may protect metastatic cancer cells against oxidative damage and in favor of their recolonization. Thus, our finding may reveal a novel mechanism of cancer cells overcoming environmental challenges in their metastasis to the lung.

In summary, metabolism in cells plays many roles beyond producing metabolites for energy generation and anabolism [34]. Metabolism alternation may influence many cellular programs, as metabolites regulate many intracellular balances and signaling pathway. Our findings further suggested the great importance of nucleotide de novo synthesis in the regulation of cancer cell stemness, adding evidence to the crosstalk of cancer cell cellular programs and metabolic networks.

## Materials and methods

### Ethics statement

All experimental procedures performed on mice were approved by the Nankai University Animal Care and Use Committee (approval number NKU20160816). All experiments complied with the guidelines for the management of laboratory of Nankai University, P R China.

### Cells

Mouse breast cancer cell line 4T1 and 4TO7 were obtained from Ralph A. Reisfeld’s laboratory. Cells from lung metastatic foci were harvested and digested using PBS containing 1% BSA and 0.2% collagenase IV (17104019, Gibc) at 37°C for 2 hours after tail vein injection with 4TO7^Ori^ cells in 6–8-week-old females. After 3 repeats, 4TO7^Lung^ cells were obtained from the lung metastatic foci.

Vector construction and virus production shRNA templates targeting *Prps2*, *Prkg*, and scrambled control (*LacZ*) were cloned into the lentivirus-based plasmid pLV-H1-EF1α-puro (SORT-B19, Biosettia Inc.) (Table 3). 4T1 and 4TO7^Lung^ cells were infected with lentivirus carrying RNAis for 24 hours and cultured for 48 hours, followed by selection using 10 μg/ml puromycin.

**Table 3:** The sequences of the RNAis used for vector construction.

shPrps2#1	AAAAGCTGGTGCCACCAAAGTTTATTTGGATCCAAATAAACTTTGGTGGCACCAGC
shPrps2#2	AAAAGCTACTCATCATGATCAATGCTTGGATCCAAGCATTGATCATGATGAGTAGC
shPrkg1	AAAAGGGCTCATTCTGACTTCATTGTTGGATCCAACAATGAAGTCAGAATGAGCCC
shPrkg2	AAAAGGATGCCGATGGCTACCTTAATTGGATCCAATTAAGGTAGCCATCGGCATCC

### Animal

All mice were maintained in an IVC library system at Nankai University. Tumor cells were resuspended in serum-free DMEM media and injected subcutaneously into the right armpit areas or tail vein of 6-week-old BALB/c females. Tumor initiation and progression were observed every 3 days.

### Clinical information

Patients’ information (*n =* 337) were collected from the Department of Oncology, Chinese PLA General Hospital, Beijing, China. Based on the condition of patients after treatment, patients were divided into improvement group and deterioration group. Conditions for improvement included a reduction in lesions by radiographic evidence, reduction of at least 2 tumor-specific markers, and a clinical response evaluation by RECIST criteria resulting in CR, PR, or SD. Deteriorating conditions include recurrence, new metastases, radiographic evidence of enlarged lesions, elevation of at least 2 tumor-specific markers, and clinical response evaluation by RECIST criteria resulting in PD.

### Statistical analyses

Survival curves were plotted using the Kaplan–Meier method. The association between prognosis and the changes in UA levels before and after treatment was tested by linear regression analyses. The odds ratios (ORs) with 95% CIs were estimated via univariate analysis to test the relationships between the changes of UA levels before and after treatment and the risk of prognosis.

All statistical analyses were performed using Empower (www.empowerstats.com, X&Y solutions, Inc., Boston, MA).

All data were shown as means ± SD. Statistical analyses were performed by a Student *t* test using GraphPad Prism software (https://www.graphpad.com/scientific-software/prism/). Statistical significance was indicated by *P ≤ 0.05, **P ≤ 0.01, and ***P ≤ 0.001.

### Limiting dilution analysis

shControl-4TO7^Lung^, shPrps2-4TO7^Lung^, 4TO7^Ori^ and 4TO7^Lung^ cells were resuspended in serum-free DMEM media at limiting dilution condition (10^3^, 10^4^, and 10^5^ per 100 ul) and injected into tail vein of 6–8-week-female mice, respectively (*n =* 6–10 per group).

### Gene expression assessment by RT-qPCR

Total RNA was extracted using TRIeasy total RNA extraction reagent (10606ES60, Yeasen) and reverse transcribed (1 μg for each group) using M-MLV reverse transcriptase (M1708, Promega). The levels of mRNA expression were quantified by RT-qPCR using Hieff UNICON qPCR SYBR Green Master Mix (11198ES08, Yeasen) after 45 cycles of PCR includes denaturation at 95°C for 30 seconds, annealing at 60°C for 45 seconds, and elongation at 72°C for 30 seconds in each cycle. The relative expression levels of genes were determined after normalization to β-Actin. The sequences of the primers used for qPCR were listed in Table 4.

**Table 4:** The sequences of the primers used for qPCR.

m-β-Actin -F	GGCTGTATTCCCCTCCATCG
m-β-Actin-R	GCACAGGGTGCTCCTCAG
m-Prps2-F	ACAAGGTAGGCGAGAGTCGTG
m-Prps2-R	AACCCTTTTGGCTCCTCCAGC
m-Adss-F	TTGCCAGCAACGCATGAGAC
m-Adss-R	CTGTGCGGCACCATGAGAAG
m-Gmps-F	CGTCAGGACTTGGTCCGCTC
m-Gmps-R	GCGACCACAGGATCAGAGGG
m-Pfas-F	TTCCCGAGAACCTTGTGCGT
m-Pfas-R	CGTACCTCTGCCGTACTCCG
m-Aprt-F	TCACCTGAAGTCCACGCACA
m-Aprt-R	GTCACAGGCCGCAAACATGG
m-Hprt-F	GGAGAGCGTTGGGCTTACCT
m-Hprt-R	GCCACAATGTGATGGCCTCC
m-Ppat-F	AGAACTGGTCACGCCCCTTC
m-Ppat-R	TCCAGAAGCGATGCACCCAA
m-Paics-F	CGTCAGGACTTGGTCCGCTC
m-Paics-R	GCGACCACAGGATCAGAGGG
m-Cad-F	GGTGGTGATGCACCCAATGC
m-Cad-R	GGAGCCTAAAGCATGGTCCC
m-Sox2-F	GAGCTAGACTCCGGGCGATG
m-Sox2-R	ACCACGAAAACGGTCTTGCC
m-Oct4-F	CTCAGTGGGGCGGTTTTGAG
m-Oct4-R	AAGGCCTCGAAGCGACAGAT
m-Nanog-F	CCTTGAGCCGTTGGCCTTCAG
m-Nanog-R	CATGTCAGTGTGATGGCGAGG
m-Klf4-F	GAAATTCGCCCGCTCCGATG
m-Klf4-R	CTCTCCTGGCAGTGTGGGTC
m-c-Myc-F	CGTTGGAAACCCCGCAGAC
m-c-Myc-R	GCGACCGCAACATAGGATGG
m-Prkg1-F	TGTGCTTAAAGATGGTCCTGGAAG
m-Prkg1-R	ACTCCACCCTACCCCAAGCA
m-Prkg2-F	CCTTTCCTCCAAAGACAAGGCAT
m-Prkg2-R	TGGCTCGATTGCCTCCTTCG
h-β-Actin-F	CGTCACCAACTGGGACGA
h-β-Actin-R	ATGGGGGAGGGCATACC
h-Prps2-F	GCGTGGAGATTGGTGAAAGC
h-Prps2-R	TTGGGGCACGACTCTCTCCT

### Protein extraction and western blot

Proteins were extracted from cells using cell lysis buffer (9803, Cell Signaling Technology) containing protease inhibitor cocktail (5892970001, Merck). The following primary antibodies were used at a dilution of 1:1,000: ACTB (YM3028, Immunoway), PRPS2 (27024-1-AP, Proteintech), ADSS (16373-1-AP, Proteintech), GMPS (16376-1-AP, Proteintech), PFAS (HPA022886, Sigma‐Aldrich), GART (sc-166379, Santa Cruz Biotechnology), CAD (16617-1-AP, Proteintech), KLF4 (11880-1-AP, Proteintech), OCT4 (11263-1-AP, Proteintech), SOX2 (11064-1-AP, Proteintech), NANOG (14259-1-AP, Proteintech), VASP (13472-1-AP, Proteintech), Phospho-VASP (Ser239) (3114, Cell Signaling Technology), ERK (sc-514302, Santa Cruz Biotechnology), and P-ERK (Thr202 + Tyr204) (BS-3016R, Bioss).

The secondary antibodies anti-mouse IgG-HRP (ZLI-2305, Zsgb-Bio, dilution 1:5,000) and anti-rabbit IgG-HRP antibodies (ZLI-2301, Zsgb-Bio, dilution 1:5,000) were incubated at room temperature for 60 minutes. ECL detection reagent (WBKLS0500, Millipore) was used according to the manufacturer’s instructions

### HE, immunohistochemistry, and IF

Tissues were fixed with 4% paraformaldehyde for at least 24 hours. For HE staining, tissues were embedded in paraffin and sectioned into 4-μm intervals (Leica). For immunohistochemistry (IHC), tissue sections were deparaffinized, rehydrated, and permeated using Triton X 100 (T8200, Solarbio) and followed by antigen retrieval using EDTA Antigen Retrieval solution (c1034, Solarbio). The primary antibodies used for IHC and IF were the same as WB (dilution 1:200). Biotinylated goat anti-rabbit IgG antibody (BA-1000, Vector Laboratories), Streptavidin, Horseradish Peroxidase, R.T.U. (SA-5704, Vector Laboratories), and DAB kit (ZLI-9017, Zsgb-Bio) were used for IHC. Alexa Fluor 488—Conjugated Goat anti-Mouse IgG (ZF0512, Zsgb-Bio), Alexa Fluor 594—Conjugated Goat anti-Rabbit IgG (ZF0516, Zsgb-Bio) and DAPI (D9542, Sigma‐Aldrich) were used for IF. Fluoro-Gel Mounting Medium with TES Buffer (17985–30, Electron Microscopy Sciences) was used for protecting the fluorescence.

### Flow cytometry

Cells for flow cytometry analysis were harvested and resuspended in PBS with 1% FBS at a density of 1.0×10^6^ cells/ml. Hoechst 33342 (B2261, Sigma‐Aldrich), verapamil (V4629, Sigma‐Aldrich), and reserpine (R0875, Sigma‐Aldrich) were used at a final concentration of 32 μM (Hoechst 33342), 8 μM (verapamil), and 8 μM (reserpine) for side population analysis. PE rat anti-mouse CD24 (553262, BD Biosciences) and FITC rat anti-mouse CD44 (553133, BD Biosciences) were diluted to 1:100 for the staining.

### ELISA analysis

cGMP (E-EL-0083) and cAMP (E-EL-0056) were measured using ELISA kits (Elabscience), according to the manufacturer’s instructions.

### Sphere formation assay

Cells were collected and resuspended in stem cell medium, serum-free DMEM F12 Media (01-170-1A, Biological Industries) supplemented with 20 ng/ml mEGF (PMG8045, Thermo Scientific), 20 ng/ml bFGF recombinant human protein (13256029, Thermo Scientific), and 1X B27 supplement (17504044, Thermo Scientific). Cells were filtered to single-cell suspension and cultured in the stem cell medium on 6-well plates (3471, Corning) at a density of 1,000 cells per well for 5–7 days.

cGMP analog (50 μM) and MEK inhibitor (0.01 μM) were replenished every 24–36 hours as needed.

### Metabolomics profiling

The frozen cellular samples (2.25×10^7^ cells/sample, 6 replications for every group) were thawed at 4°C and then mixed with 1 mL of precooled methanol/acetonitrile/ddH_2_O (2:2:1, v/v). Treated the mixture with sonication at 4°C for 30 minutes and left at −20°C for 10 minutes. Collected the supernatant after 20 minutes of centrifuge (Eppendorf 5430R) (14,000 g, 4°C). The supernatant was lyophilized under a vacuum and stored at –80°C until redissolution in 100 μL of an acetonitrile/water (1:1, v/v) solvent for ultrahigh-performance liquid chromatography equipped with quadrupole time-of-flight mass spectrometry (UHPLC–Q-TOF/MS) analysis.

UHPLC−Q-TOF-MS analysis was performed with Agilent 1290 Infinity LC UHPLC (1290 Infinity LC, Agilent Technologies, Santa Clara, California, USA) combined with a Q-TOF mass spectrometer (Triple TOF 5600+; AB SCIEX, Concord, Canada). An ACQUITY UPLC BEH Amide column (1.7 μm, 2.1 mm × 100 mm column) was used. The column, which has a 2-μL injection volume, was maintained at 25°C and eluted at a flow rate of 0.3 mL/min. The mobile phase consisted of A: ddH2O + 25 mM ammonium hydroxide + 25 mM ammonium acetate (70221, Sigma), and B: acetonitrile (1499230–935, Merck). The quality-control (QC) samples that were prepared by pooling 10 μL of each sample were placed into the column at regular intervals in the analysis sequence.

For MS detection, the following ESI source conditions were used: ion source gas 1 (Gas1) of 60 psi, ion source gas 2 (Gas2) of 60 psi, curtain gas (CUR) of 30 psi, source temperature of 600°C, and ion spray voltage floating (ISVF) of ±5,500 V (positive [ESI+] and negative [ESI−] ion modes). The MS properties were set as follows: TOF MS scan m/z range, 60–1,000 Da; product ion scan m/z range, 25–1,000 Da, TOF MS scan accumulation time; 0.20 s/spectra, product ion scan accumulation time, 0.05 s/spectra. The MS/MS data were acquired in the information-dependent acquisition (IDA) with the high-sensitivity mode. The declustering potential (DP) was ±60 V, and the collision energy (CE) was 35 V ± 15 eV. IDA was set as follows: exclude isotopes within 4 Da; candidate ions to monitor per cycle, 6. Meanwhile, the QC sample was analyzed with every batch of 5 samples to monitor precision and stability of the system and the data quality.

The raw data generated by UHPLC-Q-TOF/MS were converted into mzML format files using the ProteoWizard (http://proteowizard.sourceforge.net/). The files were subsequently processed using the XCMS online software (https://xcmsonline.scripps.edu/landing_page.php?pgcontent=mainPage) for nonlinear alignment in the time domain, automatic integration, and extraction of the peak intensities with the default measurement settings [35]. MetaboAnalyst 4.0 (http://www.metaboanalyst.ca) was employed for the statistical analysis [36]. The significance of the metabolites was ranked using the variable importance in projection (VIP) score (>1) from the partial least-squares discriminant analysis (PLS-DA) model. Specific metabolites were compared between lung metastasis group and original group.

### Metabolic flux analysis

Mouse breast cancer cells (3×10^6^) were seeded in 10-cm dishes and cultured with the glucose-deficient DMEM supplemented with 25 mM U-[^13^C]-glucose (Cambridge Isotope Laboratories) for 24 hours. For metabolite extraction, after washing cells with PBS buffer for 3 times, put the plates on dry ice and add 2 ml of 80% (v/v) prechilled methanol. Incubate the plates at −80°C for 2 hours. Scrape the plates on dry ice and transfer the cell lysate/methanol mixture to a 15-ml tube. After centrifuging at 14,000*g* for 20 minutes at 4°C, the metabolite-containing supernatant was transferred to a new tube and lyophilized at room temperature. The pallet was dissolved in 6 M guanidine hydrochloride solution and incubated at 65°C for 4 hours. The concentrations of extracted metabolites were normalized to the protein concentration measured from the guanidine hydrochloride solution using the BCA assay. Samples were analyzed using TSQ Quantiva mass spectrometry (ThermoFisher, CA) at Technology Center for Protein Sciences at Tsinghua University according to the manual instruction.

### Transcriptomics profiling

Total RNA was extracted using the method described previously and was quantified using a Nano Drop (Thermo Fisher Scientific, MA, USA). Library construction and sequencing were performed on BGIseq500 platform (BGI-Shenzhen, China). After filtering, clean reads are mapped to reference using HISAT [37] and Bowtie2 [38] tools. Gene’s expression level is quantified by a software package called RSEM [38]. NOISeq method was used to do the analysis of DEGs screening.

## Supporting information

S1 Fig Metastatic breast cancer cells in lung display increased nucleotide de novo synthesis. **A**. Changes of purine metabolism related genes between 4TO7^Ori^ and 4TO7^Lung^ cells from RNA-seq data. Purine de novo synthesis related genes (red) were up-regulated significantly whereas the salvage synthesis related genes (green) were down-regulated in 4TO7^Lung^ cells. The number under changed gene shows log2 fold-change of each gene’s expression. **B**. Changes of pyrimidine metabolism related genes between 4TO7^Ori^ and 4TO7^Lung^ cells from RNA-seq data. Pyrimidine de novo synthesis related genes (red) were up-regulated significantly whereas the salvage synthesis related genes (green) were down-regulated in 4TO7^Lung^ cells. The number under changed gene shows log2 fold-change of each gene’s expression. **C**. HE and IHC analyses of nucleotide de novo synthesis related proteins between primary foci and metastatic foci in mice followed subcutaneous injection of 4T1 cells for 40–50 days. Scale bars, 160 μm for low magnification and 40 μm for high magnification. **D**. Kaplan–Meier survival analysis of breast cancer patients with different GMPS expression levels. Information was acquired from Kaplan-Meier Plotter. **E**. Kaplan–Meier survival analysis of breast cancer patients with different PRPS2 expression levels. Information was acquired from Kaplan-Meier Plotter. **F**. Kaplan–Meier survival analysis of breast cancer patients with different CAD expression levels. Information was acquired from Kaplan-Meier Plotter. **G**. Kaplan–Meier survival analysis of breast cancer patients with different PAICS expression levels. Information was acquired from Kaplan-Meier Plotter. The raw data of **D**, **E**, **F**, and **G** can be found in S1 Fig Data. IHC, immunohistochemistry; PAICS, phosphoribosyl aminoimidazole succinocarboxamide synthetase; PRPS2, phosphoribosylpyrophosphate synthetase 2.
(TIF)

S2 Fig Knock down of PRPS2 inhibits the nucleotide de novo synthesis, lung metastasis and stemness in 4T1. **A**. Relative mRNA levels of nucleotide metabolism-related genes determined by qRT-PCR. The 4T1 cells infected with lentiviruses expressing shRNAs targeting Prps2 (shPrps2) and targeting LacZ (shControl) as control (*n =* 3 replications). **B**. The protein expression levels of nucleotide de novo synthesis genes in shControl-4T1 and shPrps2-4T1 cells. **C**. Quantification of lung metastatic foci in mice with tail vein injection of shControl-4T1 or shPrps2-4T1 cells (*n =* 3 mice in shControl group and *n =* 5 mice in shPrps2 group). **D**. Quantification of lung metastatic foci in subcutaneous injection model (*n =* 7 mice in shControl group and *n =* 8 mice in shPrps2 group). **E**. Images of lung metastases of mice at 49 days after subcutaneous injection of shControl-4T1 or shPrps2-4T1 cells. **F**. The tumor volume changes of mice after subcutaneous injection of shControl-4T1 or shPrps2-4T1 cells (*n =* 7 mice in shControl group and *n =* 8 mice in shPrps2 group). All values are presented as means ± SD. Statistical significance is shown as *P ≤ 0.05, **P ≤ 0.01, and ***P ≤ 0.001. The raw data used for quantification of **A**, **C**, **D**, and **F** can be found in S2 Fig Data. qRT-PCR, real-time quantitative polymerase chain reaction; PRPS2, phosphoribosylpyrophosphate synthetase 2.
(TIF)

S3 Fig Apoptosis resistance, EMT, motility and proliferation of 4TO7^Ori^ and 4TO7^Lung^ cells. **A**. Apoptosis analyses of 4TO7^Ori^ and 4TO7^Lung^ cells by flow cytometry (*n =* 3 replications). **B**. Quantification of early and late apoptosis in A (*n =* 3 replications). **C**. The protein expression levels of EMT-related genes in 4TO7^Ori^ and 4TO7^Lung^ cells. **D**. Migration analyses of 4TO7^Ori^ and 4TO7^Lung^ cells by transwell migration assay (*n =* 3 replications). **E**. Quantification of transwell analyses in **D**. **F**. Growth curve of 4TO7^Ori^ and 4TO7^Lung^ cells in normal medium (*n =* 3 replications). All values are presented as means ± SD. Statistical significance is shown as *P ≤ 0.05, **P ≤ 0.01, and ***P ≤ 0.001. The raw data used for quantification of **B**, **E** and **F** can be found in S3 Fig Data. EMT, epithelial to mesenchymal transition.
(TIF)

S4 Fig Sphere formation assay and quantification of shPrps2 and shControl cells. **A**. Sphere formation assay of shControl-4TO7^Lung^ and shPrps2#2-4TO7^Lung^ cells in normal sphere medium (*n =* 3 replications). **B**. Quantification of spheres per well in shControl-4TO7^Lung^ and shPrps2#2-4TO7^Lung^ cells in **A**. **C**. Sphere formation assay of shControl-4T1 and shPrps2-4T1 cells in normal sphere medium (*n =* 3 replications). **D**. Quantification of spheres per well in shControl-4T1 and shPrps2-4T1 cells in **C**. **E**. Relative mRNA levels of stemness-related genes in shControl-4TO7^Lung^ and shPrps2#2-4TO7^Lung^ cells (*n =* 3 replications). **F**. The protein expression levels of stemness-related genes in shControl-4TO7^Lung^ and shPrps2#2-4TO7^Lung^ cells. **G**. The protein expression levels of stemness-related genes in shPrps2#2-4TO7^Lung^ cells with/without cGMP analog or MEKi treatment for 72 hours. All values are presented as means ± SD. Statistical significance is shown as *P ≤ 0.05, **P ≤ 0.01, and ***P ≤ 0.001. The raw data used for quantification of **B**, **D**, and **E** can be found in S4 Fig Data. cGMP, cyclic guanosine monophosphate; MEKi, MEK inhibitor.
(TIF)

S1 Data The raw data used for quantification of Fig 1B, Fig 1F, and Fig 1I. (XLSX)

S2 Data The raw data used for quantification of Fig 2D, Fig 2E, Fig 2G, Fig 2J and Fig 2K. (XLSX)

S3 Data The raw data used for quantification of Fig 3C, Fig 3E, Fig 3F, Fig 3G, Fig 3I and Fig 3J. (XLSX)

S4 Data The raw data used for quantification of Fig 4B, Fig 4C, Fig 4D, Fig 4E, Fig 4H, Fig 4J, Fig 4L, Fig 4M, Fig 4N and Fig 4O. (XLSX)

S5 Data The raw data used for quantification of Fig 5B, Fig 5C, Fig 5D, Fig 5G, Fig 5I and Fig 5K. (XLSX)

S6 Data The raw data used for quantification of Fig 6B, Fig 6C, Fig 6H and Fig 6J. (XLSX)

S7 Data The raw data used for quantification of Fig 7A, Fig 7B, Fig 7C and Fig 7D. (XLSX)

S1 Raw images The raw data of Fig 2B. (PDF)

S2 raw images The raw data of Fig 4G, Fig 5J, Fig 6G and Fig 6I. (PDF)

S3 raw images Original western blot gel images. (PDF)

S1 Fig Data The raw data of S1D Fig, S1E Fig, S1F Fig, and S1G Fig. (PDF)

S2 Fig Data The raw data used for quantification of S2A Fig, S2C Fig, S2D Fig, and S2F Fig. (XLSX)

S3 Fig Data The raw data used for quantification of S3B Fig, S3E Fig, and S3F Fig. (XLSX)

S4 Fig Data The raw data used for quantification of S4B Fig, S4D Fig, and S4E Fig. (XLSX)

## Abbreviations

ADSS: adenylosuccinate synthetase
CAD: carbamoyl-phosphate synthetase 2
cAMP: cyclic adenosine monophosphate
cGMP: cyclic guanosine monophosphate
CSC: cancer stem cell
EMT: epithelial to mesenchymal transition
ERK: extracellular signal-regulated kinase
GMPS: guanine monophosphate synthase
GSEA: gene set enrichment assay
MEKi: MEK inhibitor
MMTV-PyMT: mouse mammary tumor virus-polyoma middle tumor-antigen
mTOR: mammalian target of rapamycin
PAICS: phosphoribosylaminoimidazole carboxylase and phosphoribosylaminoimidazolesuccinocarboxamide synthase
PPP: pentose phosphate pathway
PRPP: 5-phosphoribosyl-1-pyrophosphate
PRPS2: phosphoribosylpyrophosphate synthetase 2
qRT-PCR: real-time quantitative polymerase chain reaction
TCGA: The Cancer Genome Atlas
UA: uric acid
VASP: vasodilator-stimulated phosphoprotein
