# Supplementary material for: Nucleotide de novo synthesis increases breast cancer stemness and metastasis via cGMP-PKG-MAPK signaling pathway
Source: PLoS Biol. 2020 Nov 13;18(11):e3000872. doi: 10.1371/journal.pbio.3000872 (PMC7688141; doi:10.1371/journal.pbio.3000872)
Supplement: S1 Raw images — (PDF) [file pbio.3000872.s012.pdf]

Table: GSEA Results Summary

Dataset	degs.phenotype.cls#Lung_versus_Before.phenotype.cls#Lung_versus_Before_repos
Phenotype	phenotype.cls#Lung_versus_Before_repos
Upregulated in class	Lung
GeneSet	KEGG_PURINE_METABOLISM
Enrichment Score (ES)	0.2989301
Normalized Enrichment Score (NES)	1.7447646
Nominal p-value	0.0
FDR q-value	0.057999983
FWER p-Value	0.058

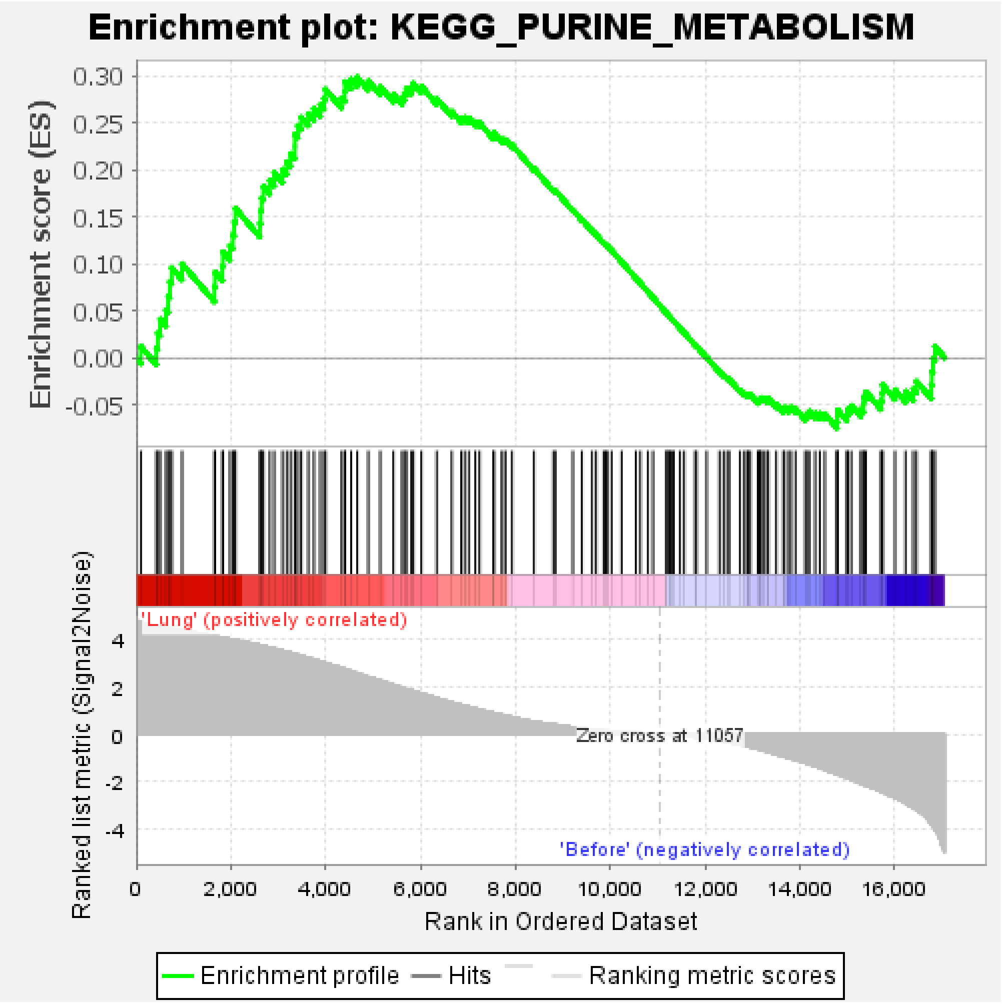

Fig 1: Enrichment plot: KEGG_PURINE_METABOLISM
Profile of the Running ES Score & Positions of GeneSet Members on the Rank Ordered List

Table: GSEA details [plain text format]

	PROBE	DESCRIPTION (from dataset)	GENE SYMBOL	GENE_TITLE	RANK IN GENE LIST	RANK METRIC SCORE	RUNNING ES	CORE ENRICHMENT
1	NT5E	NA			102	4.743	0.0122	Yes
2	ENPP1	NA			429	4.590	0.0106	Yes
3	POLR2B	NA			460	4.581	0.0264	Yes
4	POLR3F	NA			509	4.564	0.0411	Yes
5	POLR3B	NA			632	4.524	0.0512	Yes
6	ADSS	NA			680	4.511	0.0658	Yes
7	PDE4D	NA			714	4.500	0.0811	Yes
8	GMPS	NA			754	4.484	0.0960	Yes
9	PRPS2	NA			971	4.406	0.1002	Yes
10	PDE7A	NA			1655	4.164	0.0758	Yes
11	DCK	NA			1668	4.155	0.0911	Yes
12	POLE	NA			1822	4.098	0.0978	Yes
13	RRM2B	NA			1835	4.095	0.1128	Yes
14	ADCY9	NA			1982	4.034	0.1197	Yes
15	PRIM1	NA			2044	4.011	0.1315	Yes
16	NT5C2	NA			2073	4.002	0.1452	Yes
17	PAICS	NA			2098	3.996	0.1591	Yes
18	POLR2A	NA			2614	3.784	0.1432	Yes
19	RRM2	NA			2630	3.771	0.1568	Yes
20	ADCY7	NA			2650	3.762	0.1701	Yes
21	POLA1	NA			2688	3.746	0.1823	Yes
22	PDE5A	NA			2823	3.678	0.1886	Yes
23	POLR3G	NA			2916	3.632	0.1971	Yes
24	PPAT	NA			3089	3.547	0.2005	Yes
25	POLR1A	NA			3179	3.504	0.2087	Yes
26	ENPP3	NA			3259	3.463	0.2174	Yes
27	ADCY10	NA			3352	3.404	0.2250	Yes
28	NME7	NA			3353	3.402	0.2381	Yes
29	RRM1	NA			3417	3.371	0.2473	Yes
30	AMPD3	NA			3488	3.331	0.2559	Yes
31	POLR3A	NA			3635	3.246	0.2598	Yes
32	POLR1E	NA			3754	3.175	0.2650	Yes
33	POLE2	NA			3886	3.092	0.2691	Yes
34	PNPT1	NA			3967	3.044	0.2761	Yes
35	PFAS	NA			4000	3.027	0.2858	Yes
36	POLR3K	NA			4339	2.815	0.2767	Yes
37	ENTPD5	NA			4404	2.773	0.2835	Yes

38	POLD3	NA			4409	2.770	0.2940	Yes
39	PRIM2	NA			4539	2.676	0.2966	Yes
40	ADK	NA			4669	2.590	0.2989	Yes
41	NT5C3	NA			4901	2.430	0.2946	No
42	PRPS1	NA			5153	2.287	0.2886	No
43	PRUNE	NA			5431	2.102	0.2803	No
44	PDE6A	NA			5613	1.984	0.2772	No
45	ZNRD1	NA			5665	1.948	0.2816	No
46	POLE3	NA			5705	1.922	0.2867	No
47	ENTPD4	NA			5809	1.865	0.2878	No
48	NUDT2	NA			5853	1.835	0.2923	No
49	PAPSS1	NA			6022	1.733	0.2890	No
50	PDE8B	NA			6345	1.541	0.2759	No
51	POLR1B	NA			6663	1.359	0.2624	No
52	PDE4C	NA			6862	1.247	0.2555	No
53	ATIC	NA			6934	1.213	0.2559	No
54	AK4	NA			7022	1.171	0.2553	No
55	ADCY6	NA			7161	1.094	0.2513	No
56	POLR3C	NA			7249	1.047	0.2502	No
57	GMPR2	NA			7545	0.907	0.2363	No
58	XDH	NA			7548	0.906	0.2396	No
59	POLE4	NA			7718	0.827	0.2328	No
60	NUDT5	NA			7783	0.799	0.2321	No
61	GDA	NA			7924	0.732	0.2266	No
62	ADCY3	NA			8387	0.533	0.2014	No
63	PDE1A	NA			8796	0.423	0.1789	No
64	GUCY1A2	NA			8838	0.412	0.1780	No
65	PDE6D	NA			9206	0.296	0.1575	No
66	PDE6B	NA			9398	0.254	0.1472	No
67	PAPSS2	NA			9613	0.208	0.1353	No
68	PDE10A	NA			9697	0.192	0.1311	No
69	POLA2	NA			9856	0.164	0.1224	No
70	ADCY5	NA			9902	0.155	0.1204	No
71	CANT1	NA			9934	0.148	0.1191	No
72	POLR1D	NA			10022	0.132	0.1145	No
73	PDE7B	NA			10235	0.104	0.1023	No
74	ENTPD1	NA			10538	0.063	0.0847	No
75	PDE8A	NA			10631	0.060	0.0795	No
76	GUCY1B3	NA			10793	0.046	0.0702	No

77	PDE3B	NA			10898	0.032	0.0641	No
78	POLR2K	NA			11186	-0.007	0.0472	No
79	NME6	NA			11187	-0.007	0.0472	No
80	AMPD2	NA			11233	-0.018	0.0446	No
81	NME5	NA			11250	-0.023	0.0438	No
82	ADCY8	NA			11301	-0.032	0.0409	No
83	GMPR	NA			11335	-0.046	0.0392	No
84	AK1	NA			11469	-0.062	0.0315	No
85	PDE1C	NA			11556	-0.077	0.0268	No
86	ENTPD2	NA			11787	-0.119	0.0136	No
87	PDE4A	NA			11815	-0.120	0.0125	No
88	POLR2L	NA			12030	-0.175	0.0005	No
89	AMPD1	NA			12300	-0.262	-0.0144	No
90	PDE3A	NA			12386	-0.289	-0.0183	No
91	AK5	NA			12463	-0.321	-0.0216	No
92	ADCY1	NA			12506	-0.341	-0.0227	No
93	AK7	NA			12735	-0.445	-0.0345	No
94	GART	NA			12825	-0.483	-0.0379	No
95	POLR3D	NA			12885	-0.515	-0.0394	No
96	ADSL	NA			12906	-0.523	-0.0386	No
97	POLD1	NA			12952	-0.540	-0.0392	No
98	POLR2H	NA			13109	-0.618	-0.0460	No
99	NPR1	NA			13135	-0.632	-0.0451	No
100	NPR2	NA			13152	-0.643	-0.0436	No
101	PDE6G	NA			13176	-0.659	-0.0424	No
102	AK2	NA			13228	-0.691	-0.0427	No
103	PDE9A	NA			13303	-0.737	-0.0443	No
104	POLR2D	NA			13331	-0.752	-0.0430	No
105	PDE4B	NA			13499	-0.852	-0.0496	No
106	IMPDH2	NA			13649	-0.944	-0.0548	No
107	ITPA	NA			13674	-0.956	-0.0525	No
108	IMPDH1	NA			13762	-1.005	-0.0538	No
109	ADA	NA			13806	-1.026	-0.0524	No
110	ENTPD6	NA			13904	-1.086	-0.0540	No
111	NME1	NA			14110	-1.215	-0.0614	No
112	POLR2I	NA			14150	-1.239	-0.0590	No
113	POLR2J	NA			14196	-1.273	-0.0567	No
114	POLR3H	NA			14319	-1.353	-0.0588	No
115	PDE6H	NA			14417	-1.421	-0.0590	No

116	DGUOK	NA			14523	-1.497	-0.0595	No
117	POLR3GL	NA			14779	-1.696	-0.0680	No
118	POLR2G	NA			14788	-1.703	-0.0620	No
119	NUDT9	NA			14790	-1.705	-0.0555	No
120	NME3	NA			14985	-1.843	-0.0599	No
121	NT5C	NA			15010	-1.861	-0.0541	No
122	ENTPD3	NA			15081	-1.926	-0.0509	No
123	NME4	NA			15280	-2.081	-0.0546	No
124	POLR1C	NA			15335	-2.130	-0.0496	No
125	POLR2F	NA			15338	-2.131	-0.0415	No
126	ADSSL1	NA			15390	-2.170	-0.0362	No
127	GUK1	NA			15706	-2.419	-0.0455	No
128	POLD2	NA			15733	-2.443	-0.0377	No
129	NME2	NA			15743	-2.448	-0.0288	No
130	APRT	NA			16004	-2.683	-0.0339	No
131	NT5M	NA			16226	-2.920	-0.0357	No
132	POLR2C	NA			16386	-3.117	-0.0332	No
133	POLR2E	NA			16457	-3.205	-0.0250	No
134	PNP	NA			16768	-3.804	-0.0287	No
135	POLD4	NA			16780	-3.833	-0.0146	No
136	PDE2A	NA			16803	-3.908	-0.0009	No
137	PDE1B	NA			16847	-4.035	0.0121	No

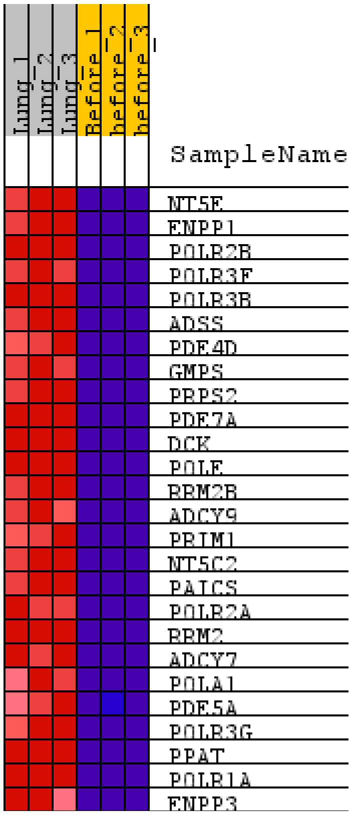

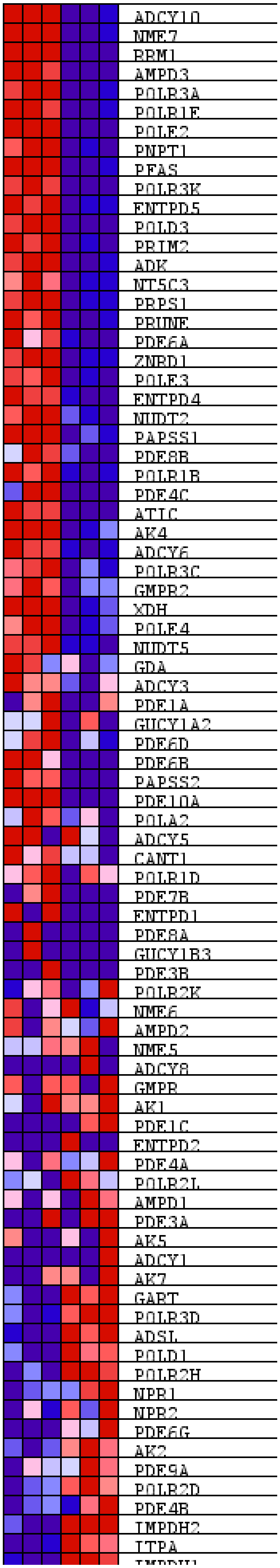
ADCY10
NME7
RRM1
AMPD3
POLR3A
POLR1E
POLE2
PNPT1
PFAS
POLR3K
ENTPD5
POLD3
PRIM2
ADK
NT5C3
PRPS1
PRUNE
PDE6A
ZNRD1
POLE3
ENTPD4
NUDT2
PAPSS1
PDE8B
POLR1B
PDE4C
ATIC
AK4
ADCY6
POLR3C
GMPR2
XDH
POLE4
NUDT5
GDA
ADCY3
PDE1A
GUCY1A2
PDE6D
PDE6B
PAPSS2
PDE10A
POLA2
ADCY5
CANT1
POLR1D
PDE7B
ENTPD1
PDE8A
GUCY1B3
PDE3B
POLR2K
NME6
AMPD2
NME5
ADCY8
GMPR
AK1
PDE1C
ENTPD2
PDE4A
POLR2L
AMPD1
PDE3A
AK5
ADCY1
AK7
GART
POLR3D
ADSL
POLD1
POLR2H
NPR1
NPR2
PDE6G
AK2
PDE9A
POLR2D
PDE4B
IMPDH2
ITPA

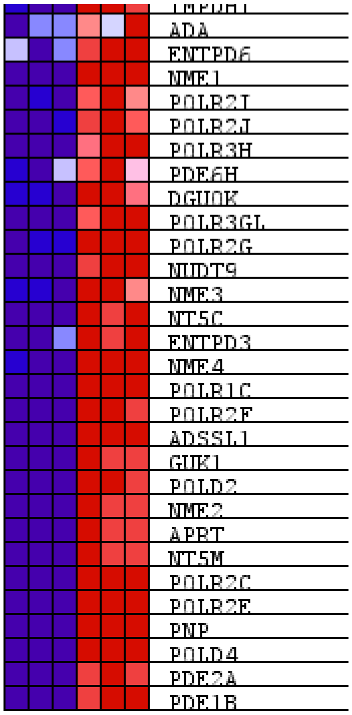

Fig 2: KEGG_PURINE_METABOLISM
Blue-Pink O' Gram in the Space of the Analyzed GeneSet

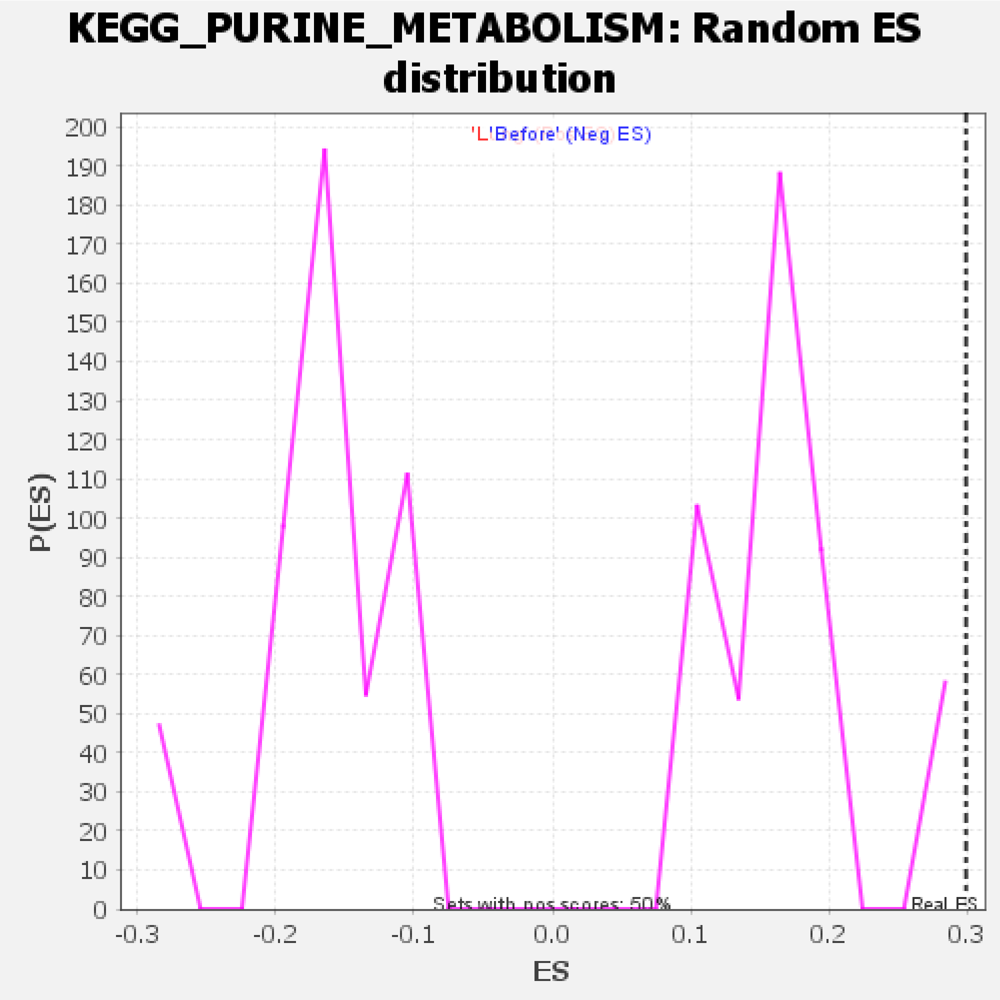

Fig 3: KEGG_PURINE_METABOLISM: Random ES distribution
Gene set null distribution of ES for KEGG_PURINE_METABOLISM

Table: GSEA Results Summary

Dataset	4TO7 2019.4TO7 2019.CLS #Lung_versus_Before.4TO7 2019.CLS #Lung_versus_Before_repos
Phenotype	4TO7 2019.CLS#Lung_versus_Before_repos
Upregulated in class	Lung
GeneSet	KEGG_PYRIMIDINE_METABOLISM
Enrichment Score (ES)	0.25462112
Normalized Enrichment Score (NES)	1.3129379
Nominal p-value	0.0945946
FDR q-value	0.11280626
FWER p-Value	0.947

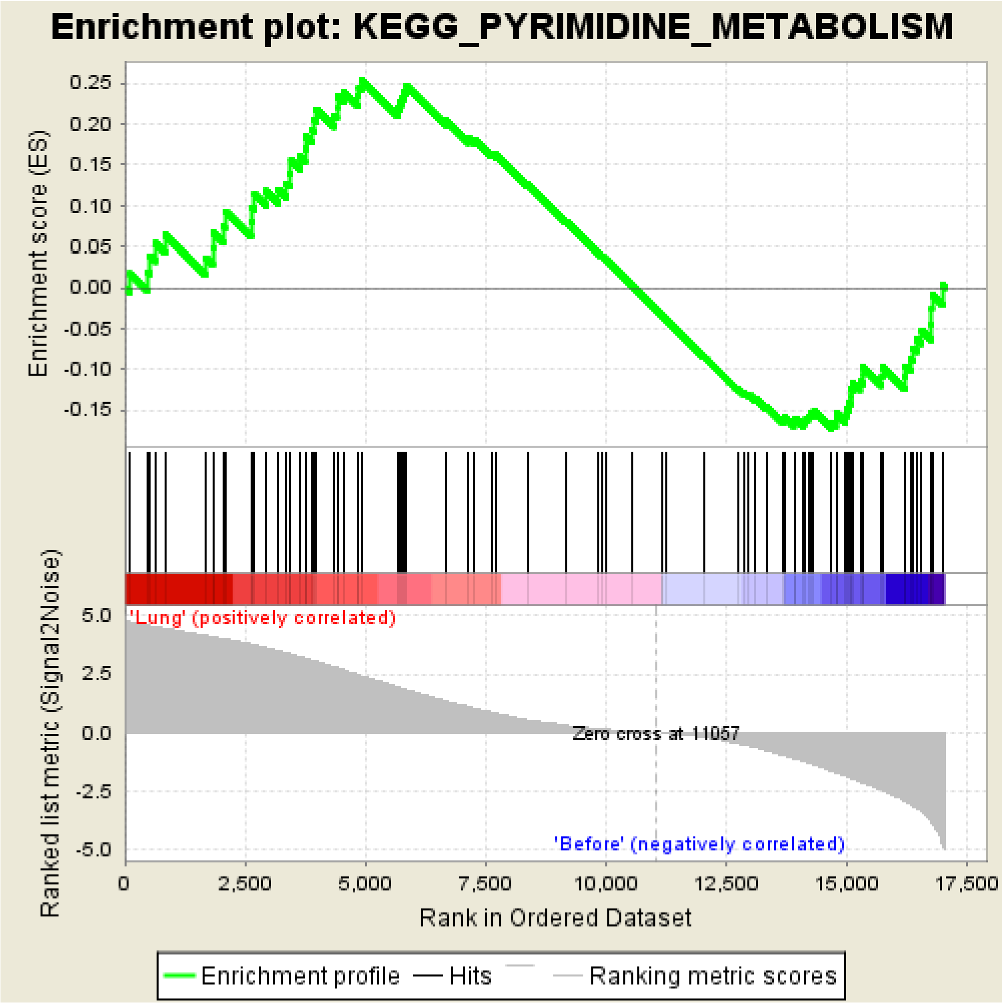

Fig 1: Enrichment plot: KEGG_PYRIMIDINE_METABOLISM
Profile of the Running ES Score & Positions of GeneSet Members on the Rank Ordered List

Table: GSEA details [plain text format]

	PROBE	DESCRIPTION (from dataset)	GENE SYMBOL	GENE_TITLE	RANK IN GENE	RANK METRIC	RUNNING ES	CORE ENRICHMENT

					LIST	SCORE		
1	NT5E	NA			102	4.743	0.0176	Yes
2	POLR2B	NA			460	4.581	0.0193	Yes
3	POLR3F	NA			509	4.564	0.0392	Yes
4	POLR3B	NA			632	4.524	0.0545	Yes
5	UPRT	NA			834	4.455	0.0648	Yes
6	DCK	NA			1668	4.155	0.0364	Yes
7	POLE	NA			1822	4.098	0.0478	Yes
8	RRM2B	NA			1835	4.095	0.0674	Yes
9	PRIM1	NA			2044	4.011	0.0751	Yes
10	NT5C2	NA			2073	4.002	0.0934	Yes
11	POLR2A	NA			2614	3.784	0.0804	Yes
12	RRM2	NA			2630	3.771	0.0982	Yes
13	POLA1	NA			2688	3.746	0.1135	Yes
14	POLR3G	NA			2916	3.632	0.1182	Yes
15	POLR1A	NA			3179	3.504	0.1202	Yes
16	NME7	NA			3353	3.402	0.1269	Yes
17	RRM1	NA			3417	3.371	0.1400	Yes
18	TXNRD1	NA			3442	3.359	0.1553	Yes
19	POLR3A	NA			3635	3.246	0.1601	Yes
20	CMPK1	NA			3739	3.188	0.1699	Yes
21	POLR1E	NA			3754	3.175	0.1849	Yes
22	POLE2	NA			3886	3.092	0.1925	Yes
23	AK3	NA			3930	3.064	0.2052	Yes
24	PNPT1	NA			3967	3.044	0.2182	Yes
25	POLR3K	NA			4339	2.815	0.2104	Yes
26	ENTPD5	NA			4404	2.773	0.2204	Yes
27	POLD3	NA			4409	2.770	0.2339	Yes
28	PRIM2	NA			4539	2.676	0.2397	Yes
29	DCTD	NA			4843	2.465	0.2341	Yes
30	CAD	NA			4857	2.453	0.2455	Yes
31	CTPS2	NA			4908	2.427	0.2546	Yes
32	ZNRD1	NA			5665	1.948	0.2197	No
33	POLE3	NA			5705	1.922	0.2270	No
34	DPYS	NA			5769	1.889	0.2327	No
35	ENTPD4	NA			5809	1.865	0.2397	No
36	NUDT2	NA			5853	1.835	0.2463	No
37	POLR1B	NA			6663	1.359	0.2053	No
38	UCK2	NA			7150	1.100	0.1822	No
39	POLR3C	NA			7249	1.047	0.1816	No
40	UPB1	NA			7621	0.875	0.1641	No
41	POLE4	NA			7718	0.827	0.1625	No

42	TYMP	NA			8381	0.536	0.1262	No
43	UMPS	NA			9187	0.301	0.0802	No
44	POLA2	NA			9856	0.164	0.0416	No
45	CANT1	NA			9934	0.148	0.0378	No
46	POLR1D	NA			10022	0.132	0.0334	No
47	ENTPD1	NA			10538	0.063	0.0033	No
48	POLR2K	NA			11186	-0.007	-0.0348	No
49	NME6	NA			11187	-0.007	-0.0348	No
50	NME5	NA			11250	-0.023	-0.0383	No
51	POLR2L	NA			12030	-0.175	-0.0833	No
52	TK1	NA			12760	-0.456	-0.1241	No
53	POLR3D	NA			12885	-0.515	-0.1288	No
54	POLD1	NA			12952	-0.540	-0.1300	No
55	POLR2H	NA			13109	-0.618	-0.1361	No
56	POLR2D	NA			13331	-0.752	-0.1454	No
57	ITPA	NA			13674	-0.956	-0.1608	No
58	DTYMK	NA			13715	-0.975	-0.1583	No
59	ENTPD6	NA			13904	-1.086	-0.1640	No
60	TYMS	NA			13936	-1.107	-0.1603	No
61	NME1	NA			14110	-1.215	-0.1645	No
62	POLR2I	NA			14150	-1.239	-0.1606	No
63	POLR2J	NA			14196	-1.273	-0.1569	No
64	DUT	NA			14237	-1.303	-0.1528	No
65	POLR3H	NA			14319	-1.353	-0.1509	No
66	UPP2	NA			14687	-1.621	-0.1644	No
67	POLR3GL	NA			14779	-1.696	-0.1614	No
68	POLR2G	NA			14788	-1.703	-0.1534	No
69	NME3	NA			14985	-1.843	-0.1557	No
70	NT5C	NA			15010	-1.861	-0.1479	No
71	TXNRD2	NA			15053	-1.905	-0.1409	No
72	ENTPD3	NA			15081	-1.926	-0.1329	No
73	DHODH	NA			15086	-1.928	-0.1235	No
74	TK2	NA			15132	-1.962	-0.1164	No
75	NME4	NA			15280	-2.081	-0.1147	No
76	POLR1C	NA			15335	-2.130	-0.1073	No
77	POLR2F	NA			15338	-2.131	-0.0969	No
78	POLD2	NA			15733	-2.443	-0.1079	No
79	NME2	NA			15743	-2.448	-0.0963	No
80	UCK1	NA			16218	-2.909	-0.1097	No
81	NT5M	NA			16226	-2.920	-0.0956	No
82	UCKL1	NA			16328	-3.046	-0.0864	No
83	POLR2C	NA			16386	-3.117	-0.0743	No

84	POLR2E	NA			16457	-3.205	-0.0625	No
85	CDA	NA			16564	-3.353	-0.0520	No
86	PNP	NA			16768	-3.804	-0.0451	No
87	UPP1	NA			16775	-3.818	-0.0264	No
88	POLD4	NA			16780	-3.833	-0.0076	No
89	CMPK2	NA			16996	-4.728	0.0032	No

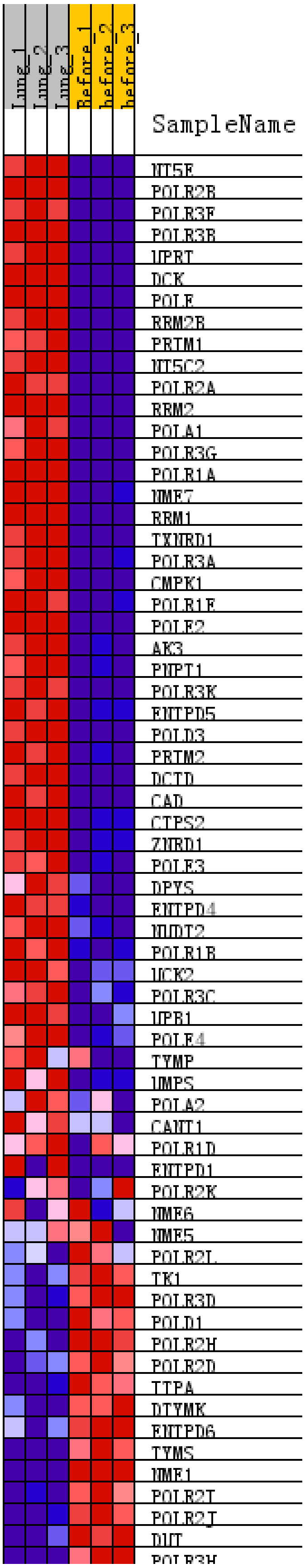

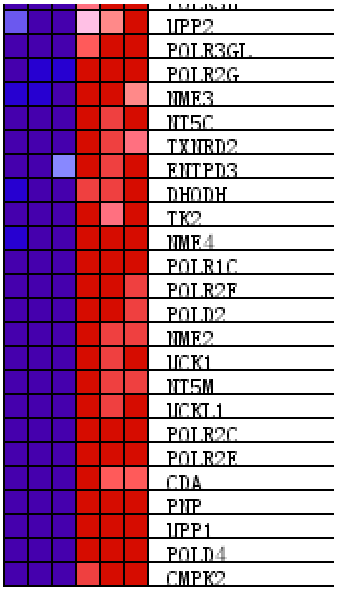

Fig 2: KEGG_PYRIMIDINE_METABOLISM
Blue-Pink O' Gram in the Space of the Analyzed GeneSet

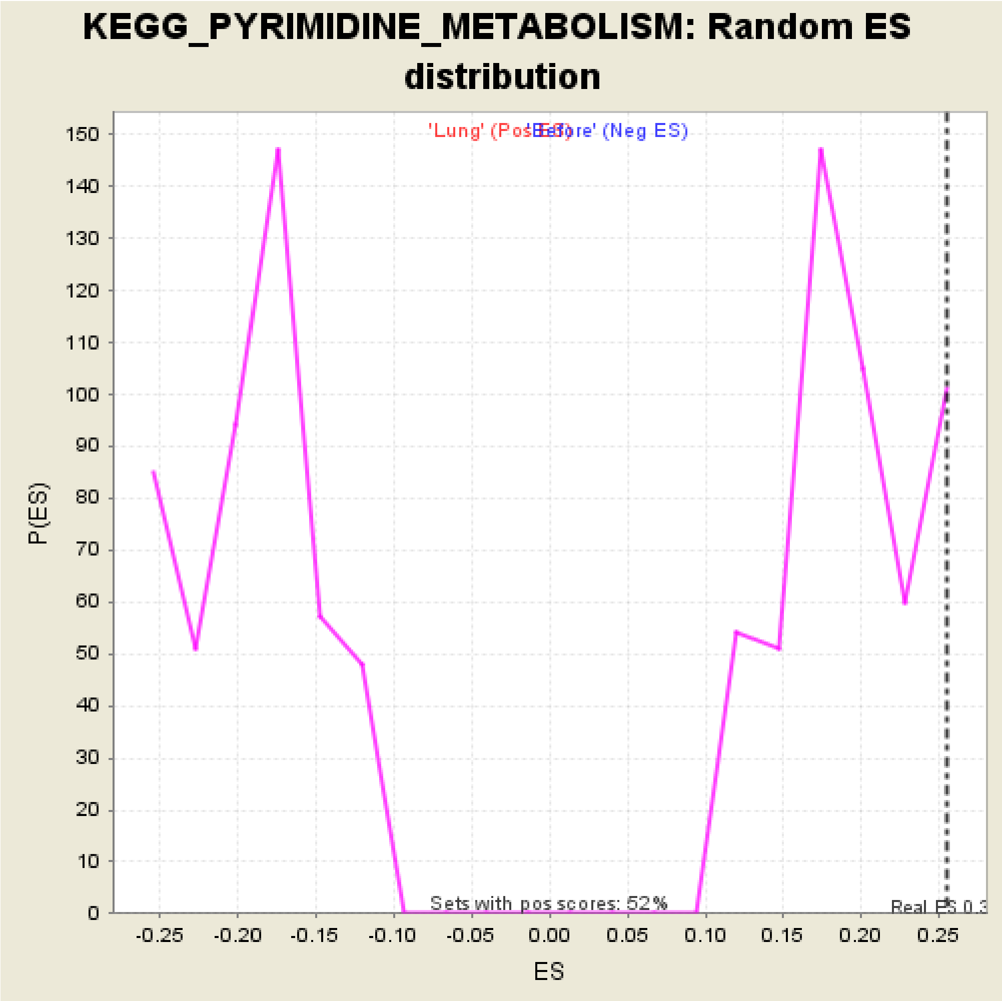

Fig 3: KEGG_PYRIMIDINE_METABOLISM: Random ES distribution
Gene set null distribution of ES for KEGG_PYRIMIDINE_METABOLISM
