# Supplementary material for: Nucleotide de novo synthesis increases breast cancer stemness and metastasis via cGMP-PKG-MAPK signaling pathway
Source: PLoS Biol. 2020 Nov 13;18(11):e3000872. doi: 10.1371/journal.pbio.3000872 (PMC7688141; doi:10.1371/journal.pbio.3000872)

F4 G, H. 4TO7^{Ori} / 4TO7 ^{Lung} side population

4TO7^{Ori}
side population

1

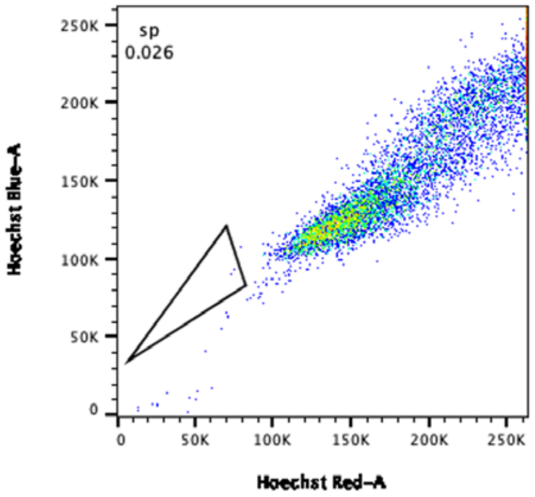

Specimen_001_Tube_005.fcs
main
7584

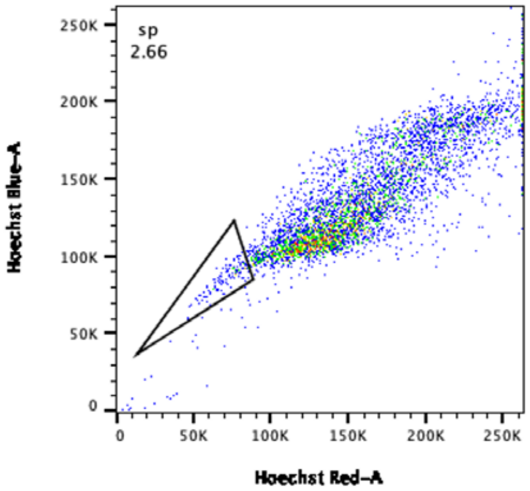

Specimen_001_Tube_006.fcs
main
4817

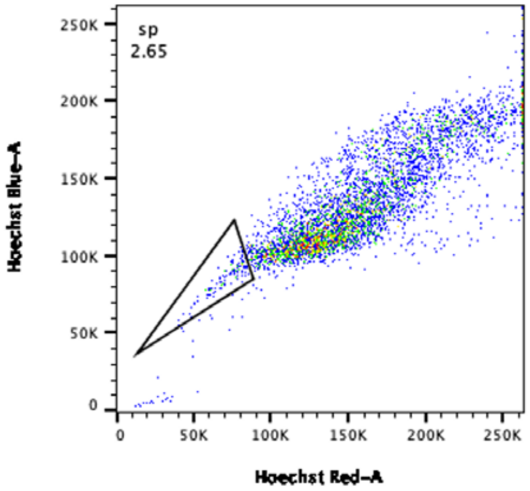

Specimen_001_Tube_008.fcs
main
4785

2

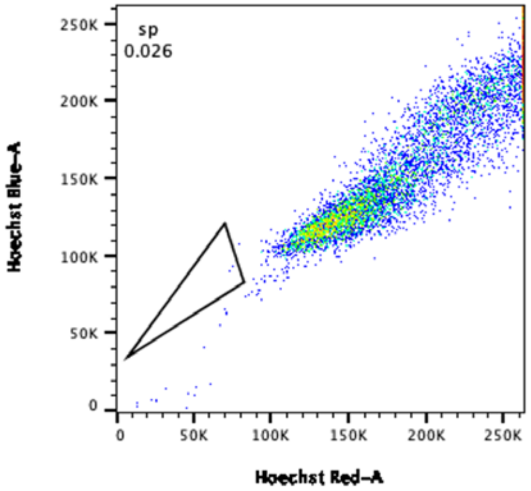

Specimen_001_Tube_005.fcs
main
7584

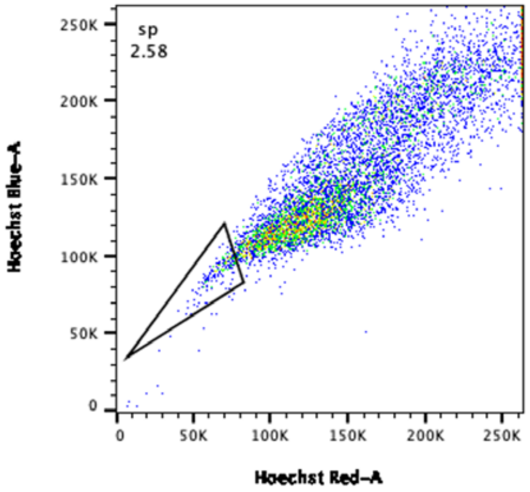

Specimen_001_Tube_007.fcs
main
7401

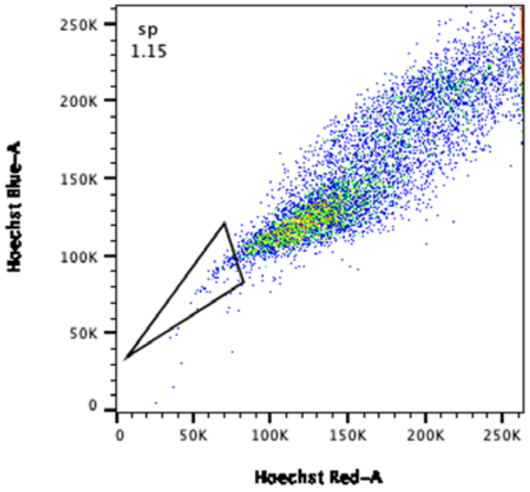

Specimen_001_Tube_008.fcs
main
7309

4TO7^{Ori}
side population

3

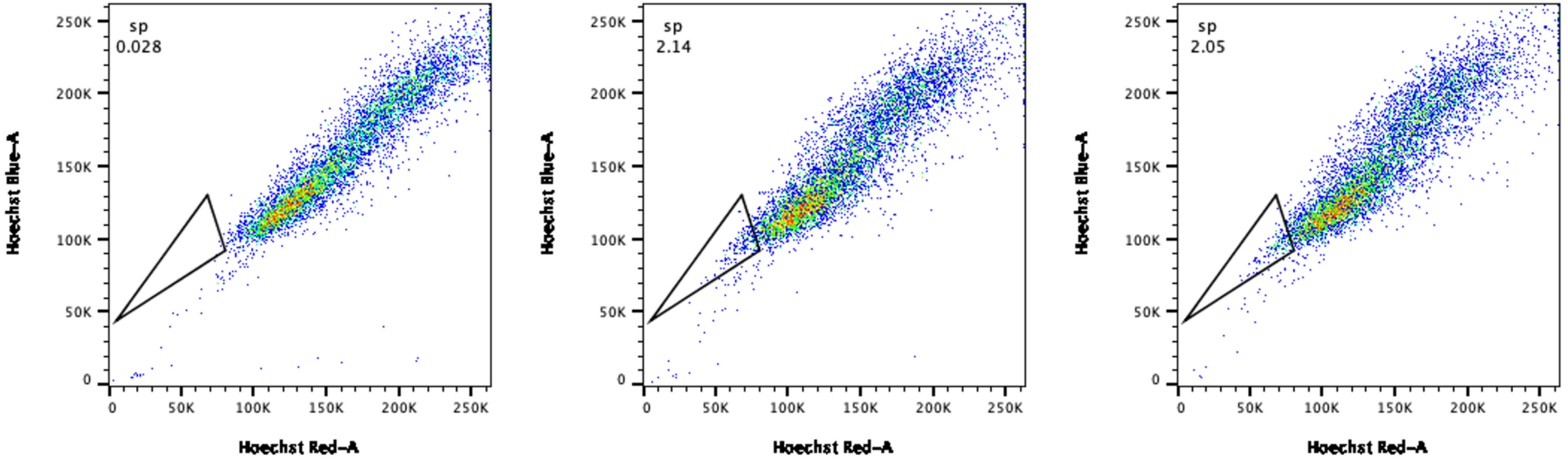

Specimen_001_Tube_001.fcs
main
7206

Specimen_001_Tube_002.fcs
main
7394

Specimen_001_Tube_003.fcs
main
7223

4

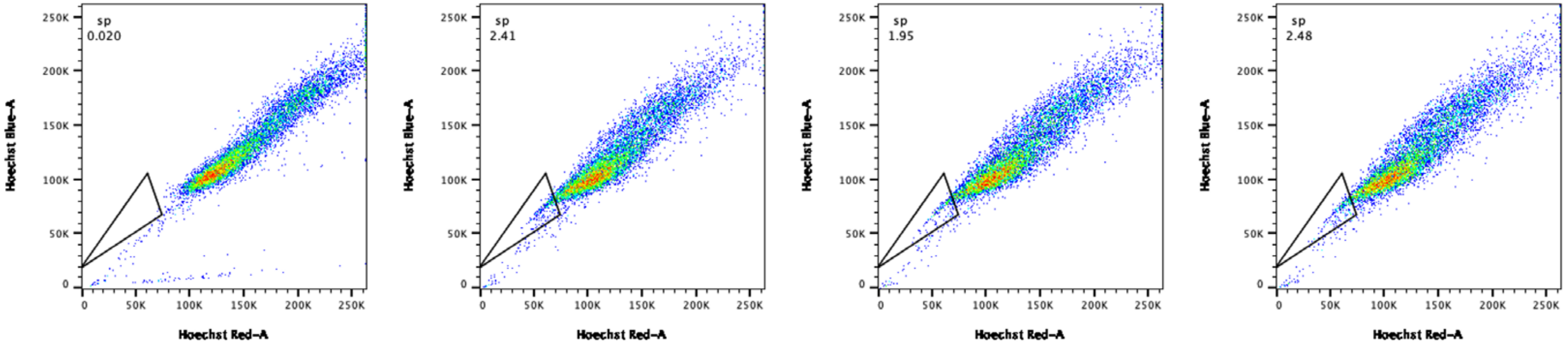

Specimen_001_Tube_001.fcs
main
10058

Specimen_001_Tube_002.fcs
main
10061

Specimen_001_Tube_003.fcs
main
9996

Specimen_001_Tube_004.fcs
main
10039

4TO7^{Lung}
side population

1

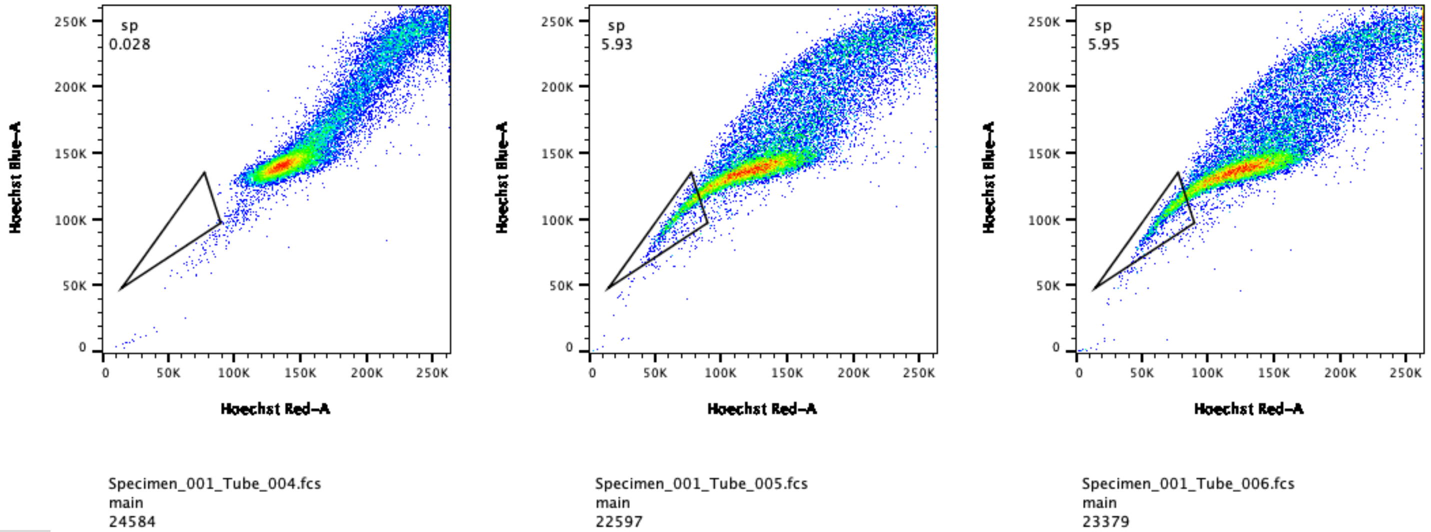

2

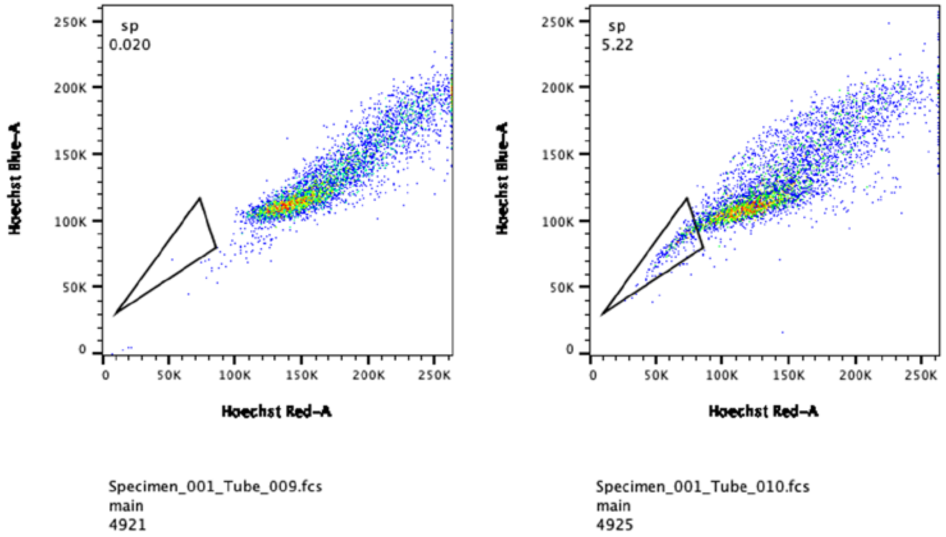

3

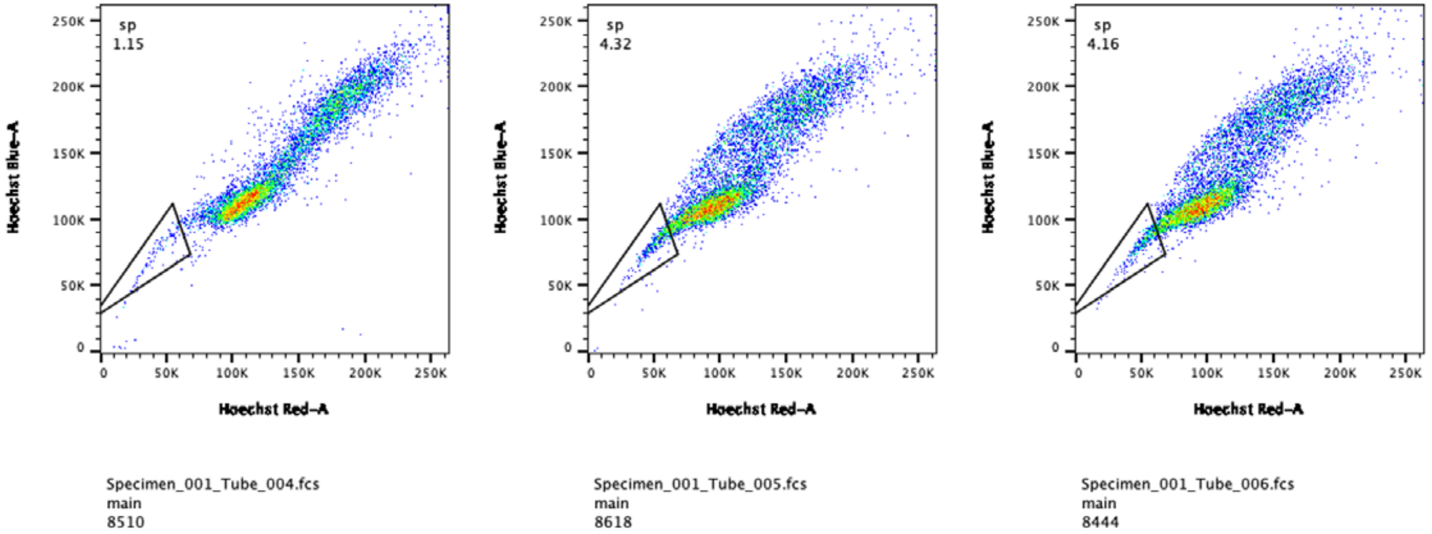

4TO7^{Lung} side population

4

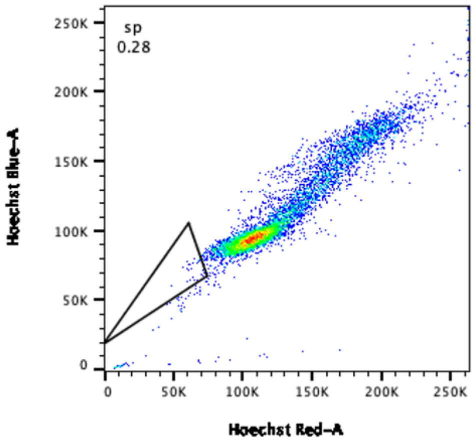

Specimen_001_Tube_005.fcs
main
10179

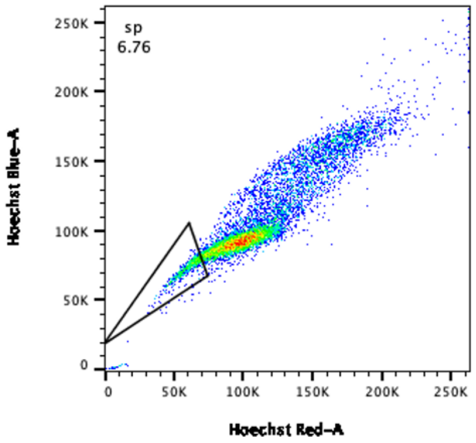

Specimen_001_Tube_006.fcs
main
10147

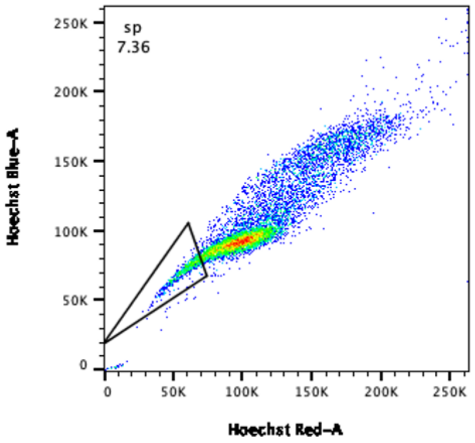

Specimen_001_Tube_007.fcs
main
10048

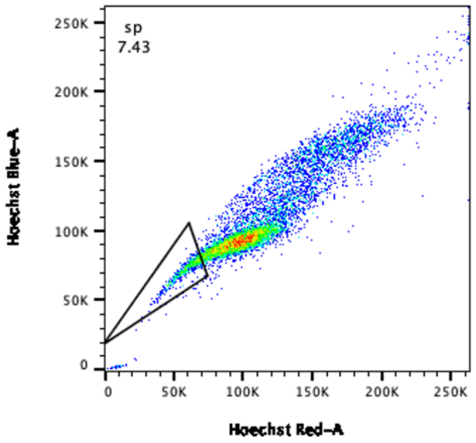

Specimen_001_Tube_008.fcs
main
10040

5

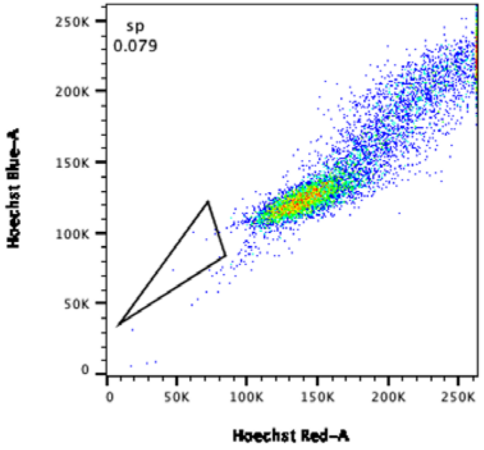

Specimen_001_Tube_009.fcs
main
7639

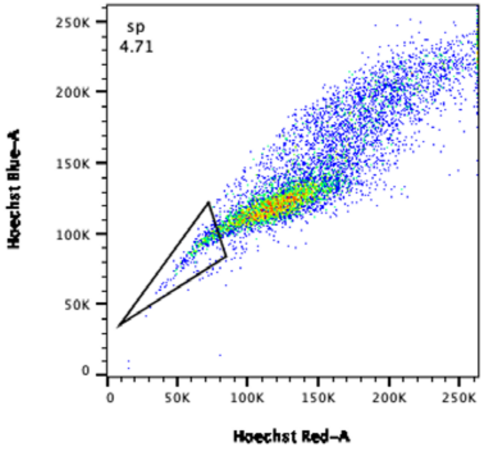

Specimen_001_Tube_010.fcs
main
7811

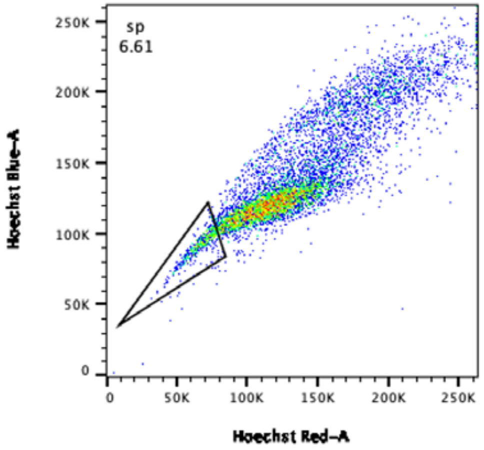

Specimen_001_Tube_011.fcs
main
8003

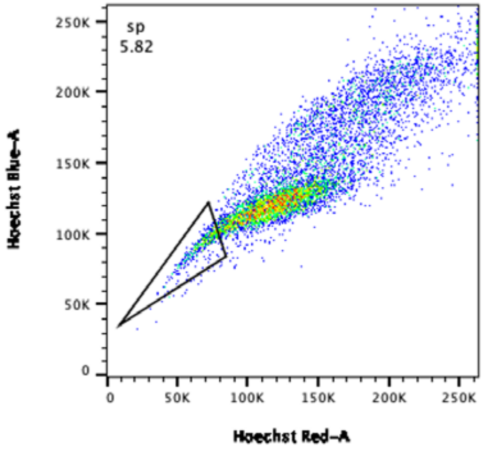

Specimen_001_Tube_012.fcs
main
7975

F5 J, K. 4TO7^{Lung}-shCtrl / shPKG side population

4TO7^{Lung}-shCtrl side population

1

2

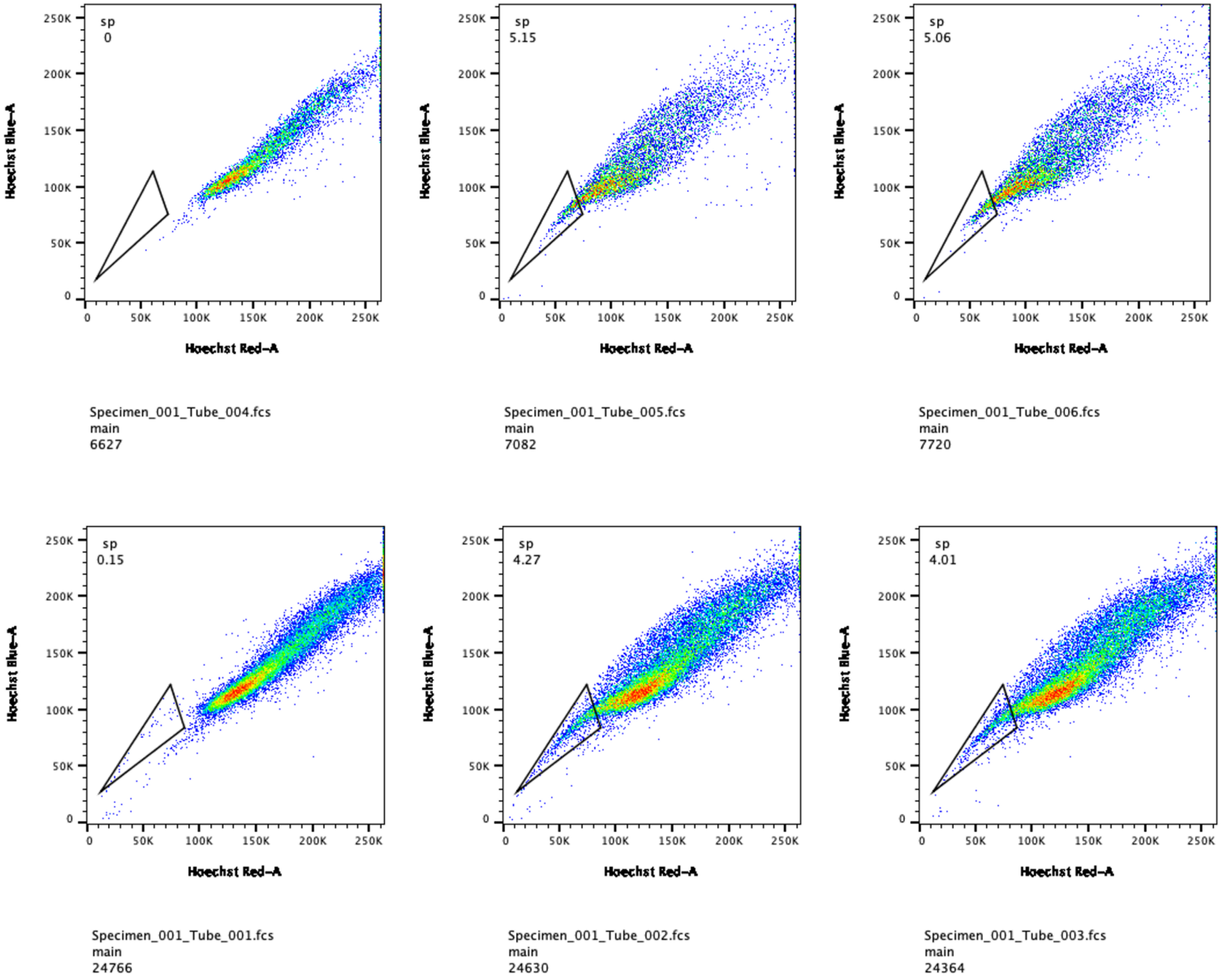

4TO7^{Lung}-shPKG side population

1

2

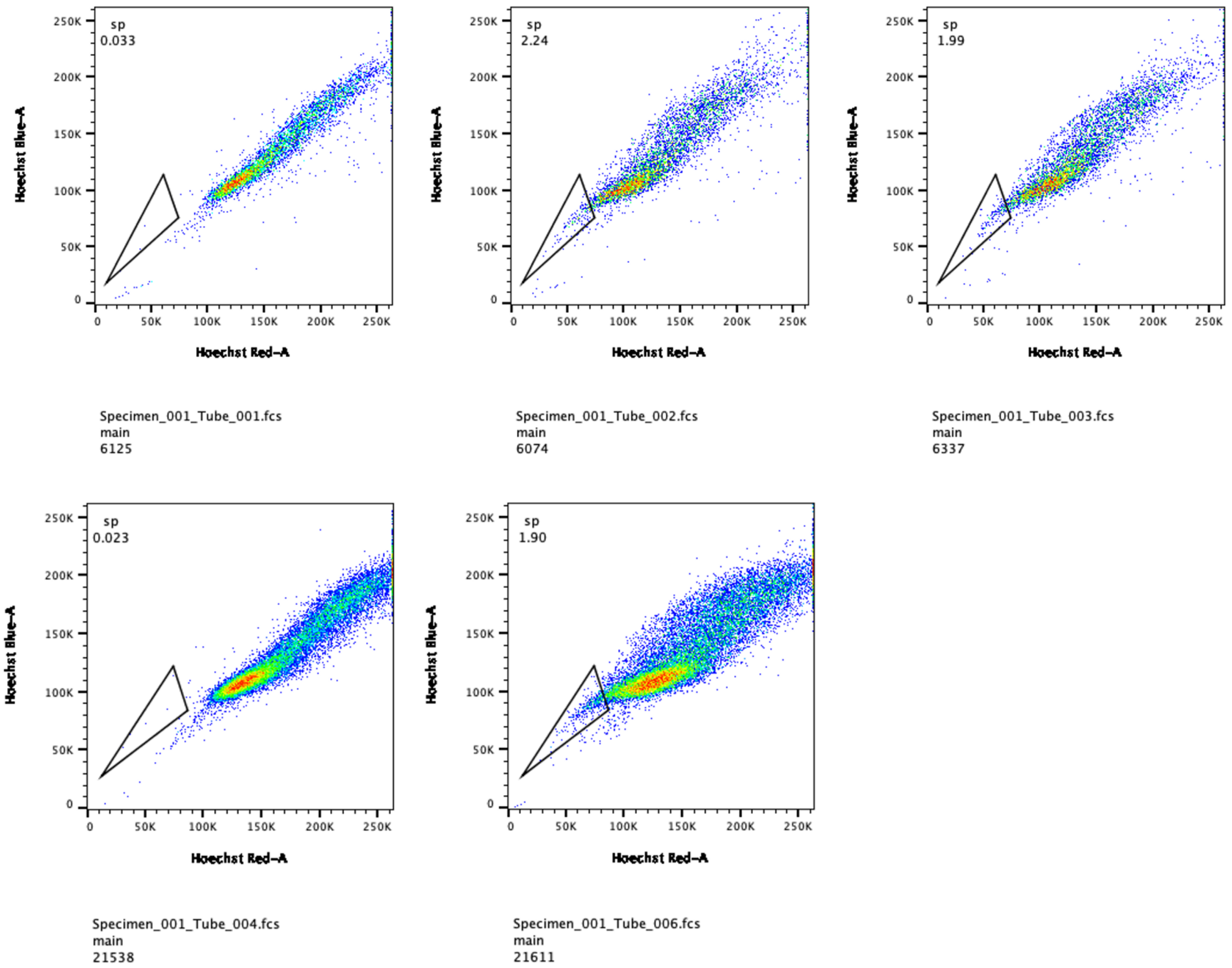

F6 G, H. 4TO7^{Lung} Ctrl / MEKi 0.01 µM / 1.0 µM side population

4TO7^{Lung} Ctrl
side population

1

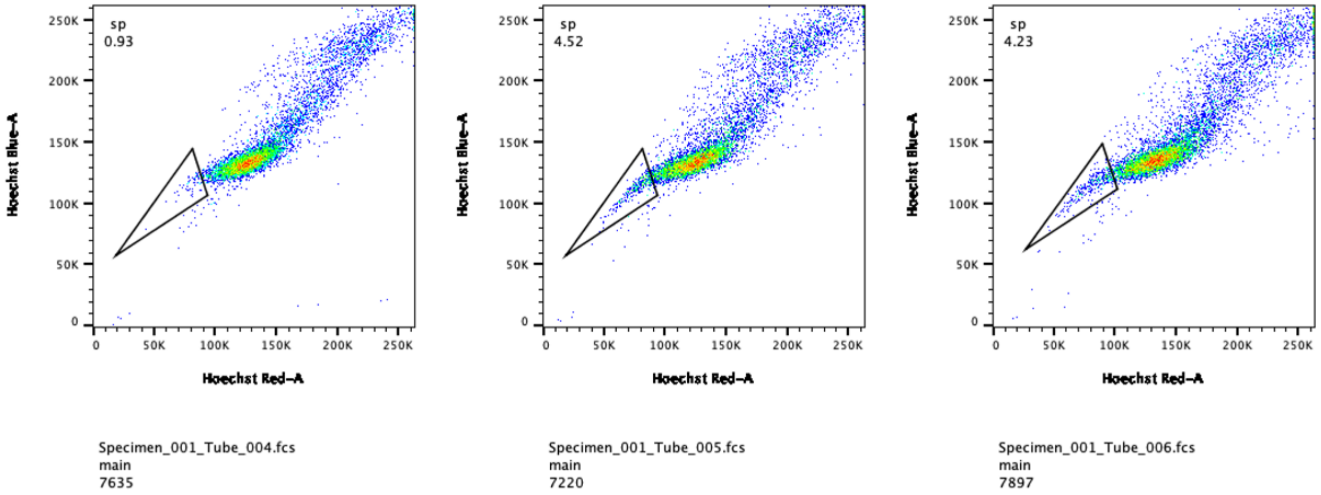

2

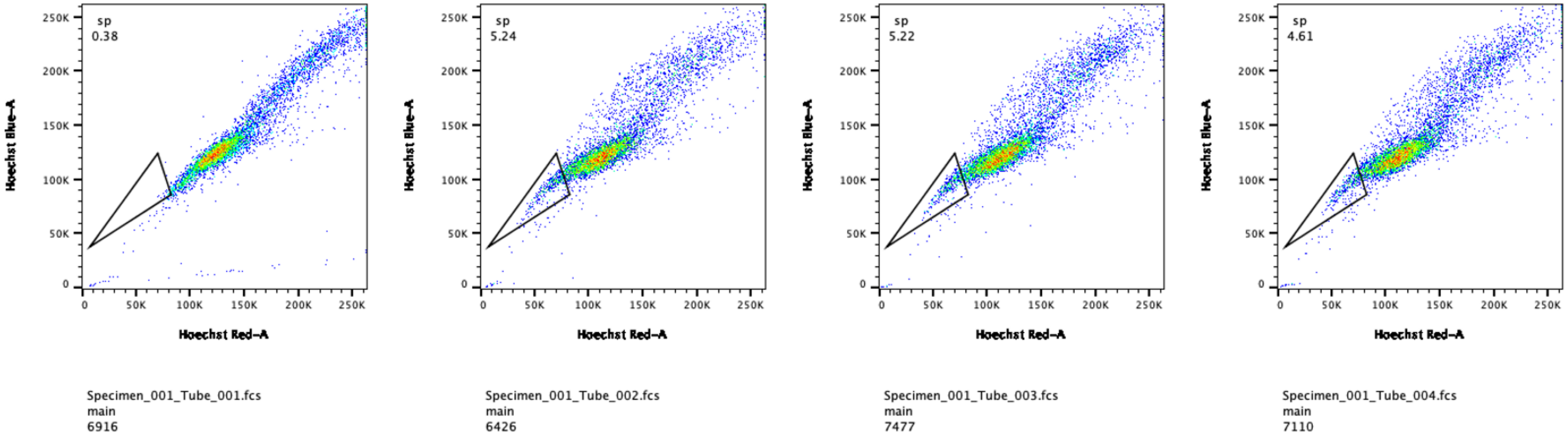

4TO7[Lung] MEKi
0.01 µM
side population

1

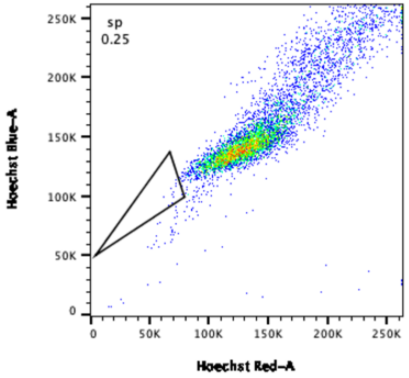

Specimen_001_Tube_001.fcs
main
6511

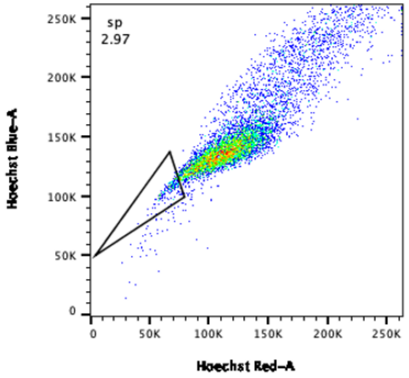

Specimen_001_Tube_002.fcs
main
6997

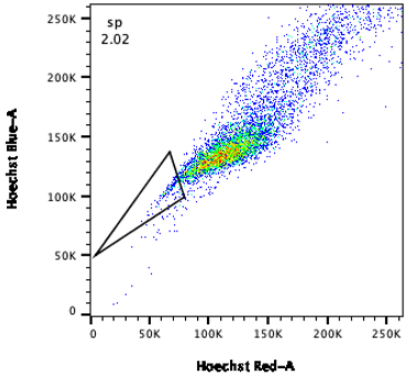

Specimen_001_Tube_003.fcs
main
6588

2

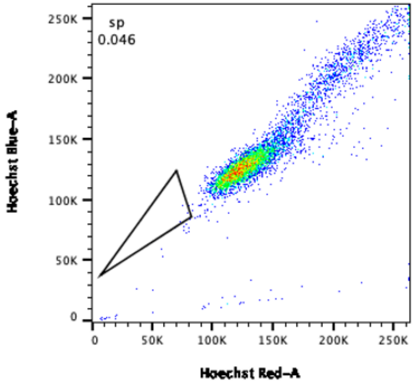

Specimen_001_Tube_005.fcs
main
6514

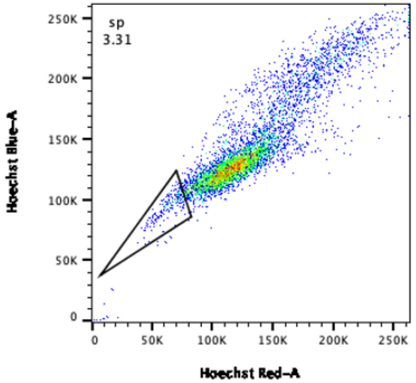

Specimen_001_Tube_006.fcs
main
6336

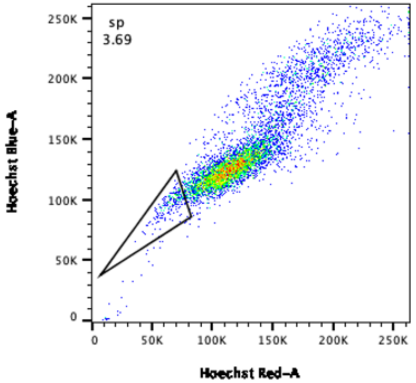

Specimen_001_Tube_007.fcs
main
6431

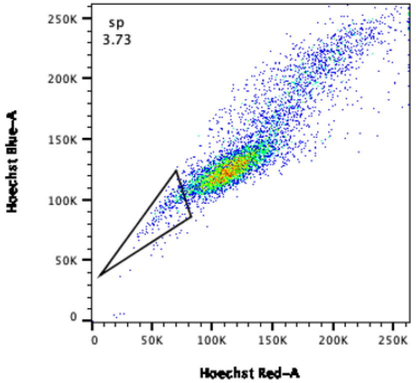

Specimen_001_Tube_008.fcs
main
6353

4TO7^{Lung} MEKi
1 µM
side population

1

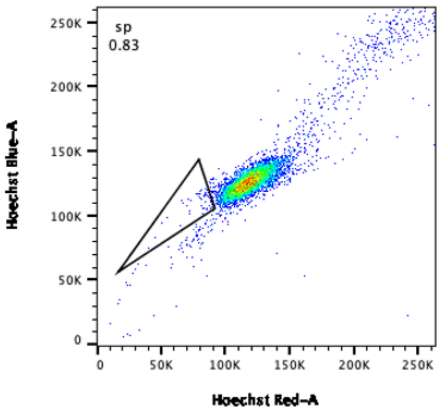

Specimen_001_Tube_007.fcs
main
6476

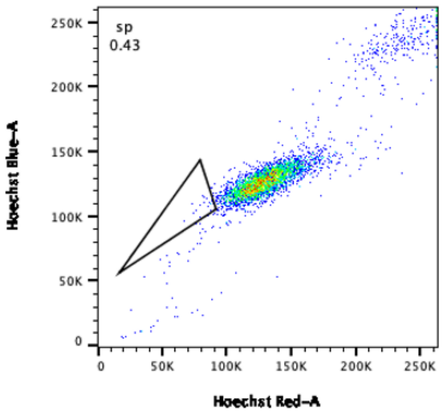

Specimen_001_Tube_012.fcs
main
4440

2

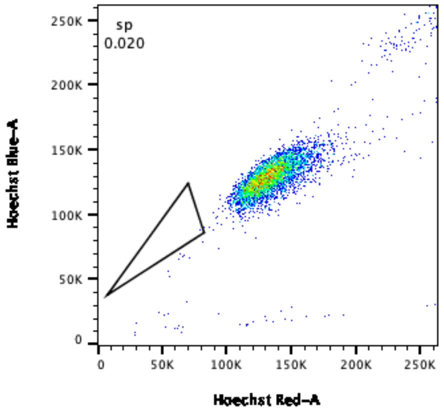

Specimen_001_Tube_013.fcs
main
5061

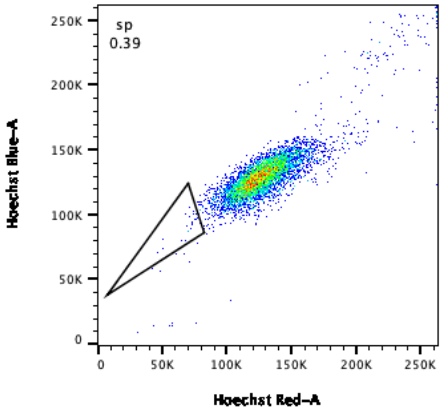

Specimen_001_Tube_014.fcs
main
5974

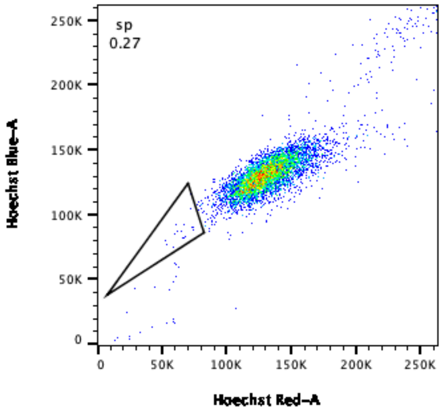

Specimen_001_Tube_015.fcs
main
5960

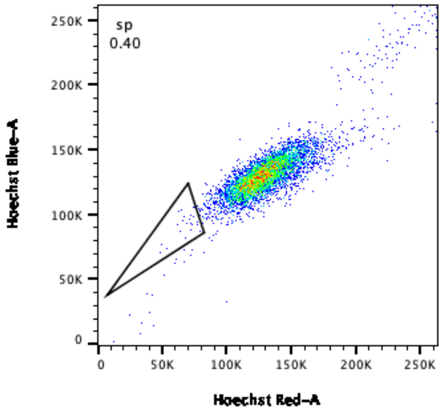

Specimen_001_Tube_016.fcs
main
5928

F6 I, J. 4TO7[Ori] Ctrl / cGMP analog 50 µM ± MEKi 0.01 µM side population

4TO7^{Ori} Ctrl
side population

1

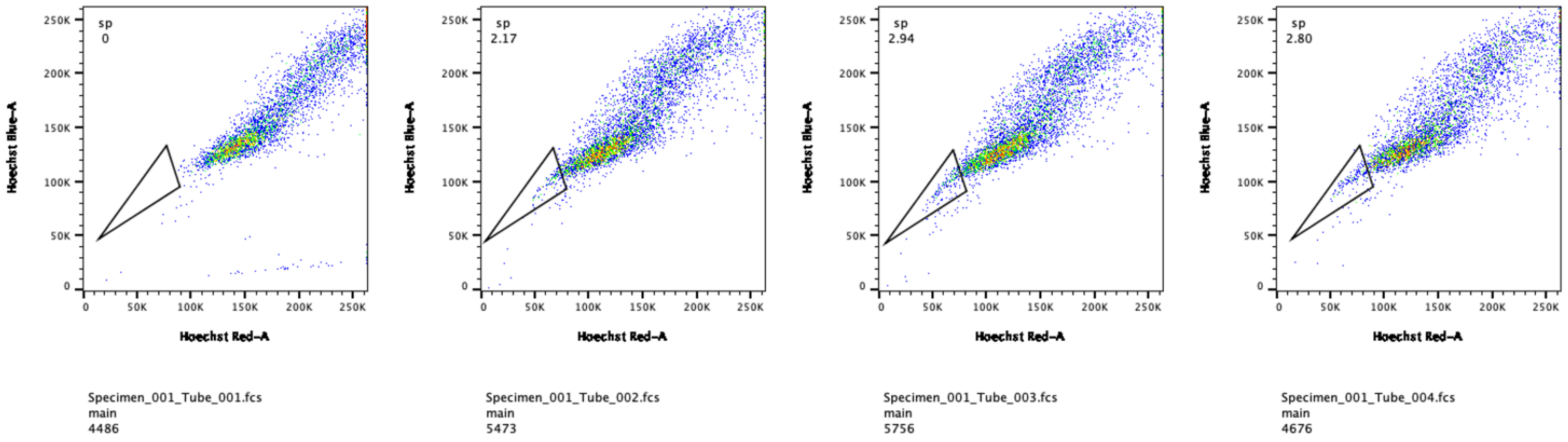

2

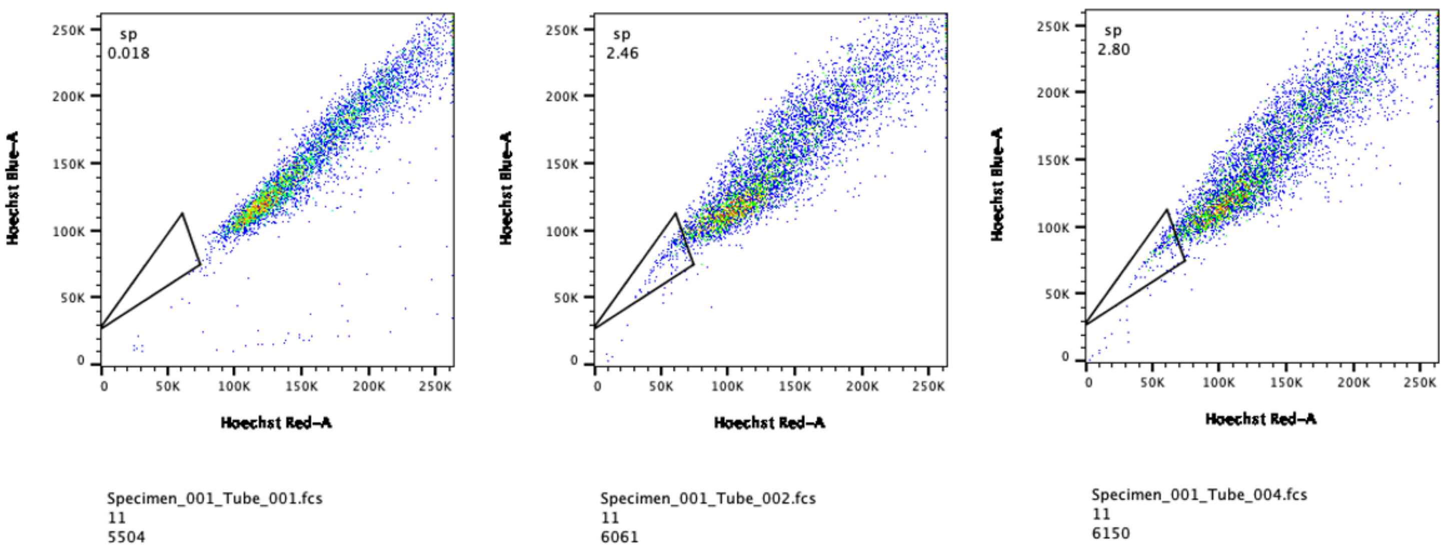

1

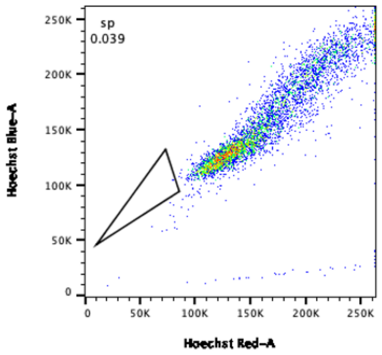

Specimen_001_Tube_006.fcs
main
5149

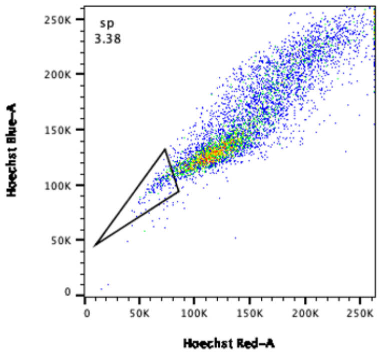

Specimen_001_Tube_007.fcs
main
5894

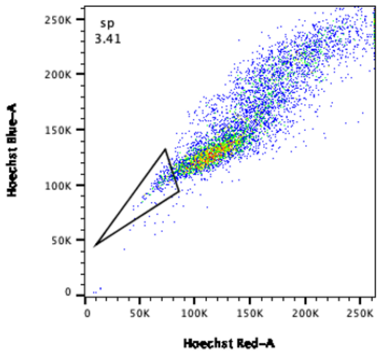

Specimen_001_Tube_008.fcs
main
5864

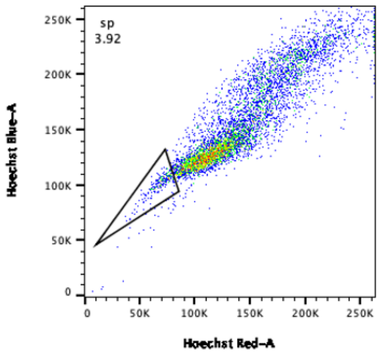

Specimen_001_Tube_009.fcs
main
5899

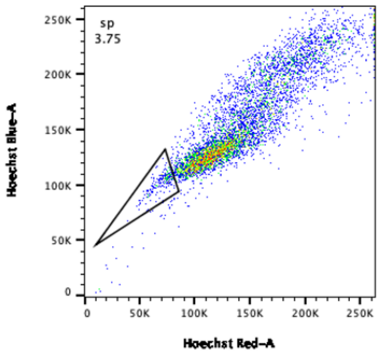

Specimen_001_Tube_010.fcs
main
5791

4TO7^{Ori} cGMF
analog 50 µM
side population

2

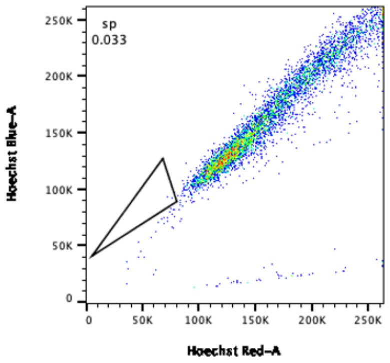

Specimen_001_Tube_006.fcs
11
6077

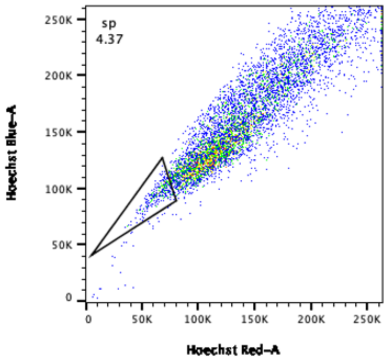

Specimen_001_Tube_008.fcs
11
6309

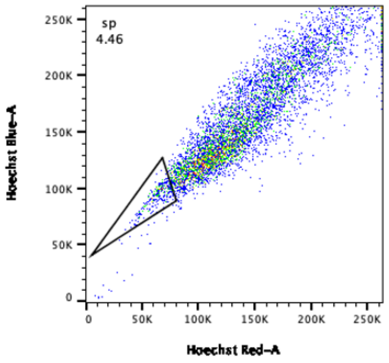

Specimen_001_Tube_009.fcs
11
6363

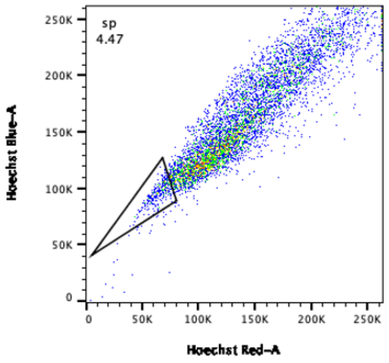

Specimen_001_Tube_010.fcs
11
6548

4TO7^{Ori} cGMP analog 50 µM + MEKi 0.01 µM side population

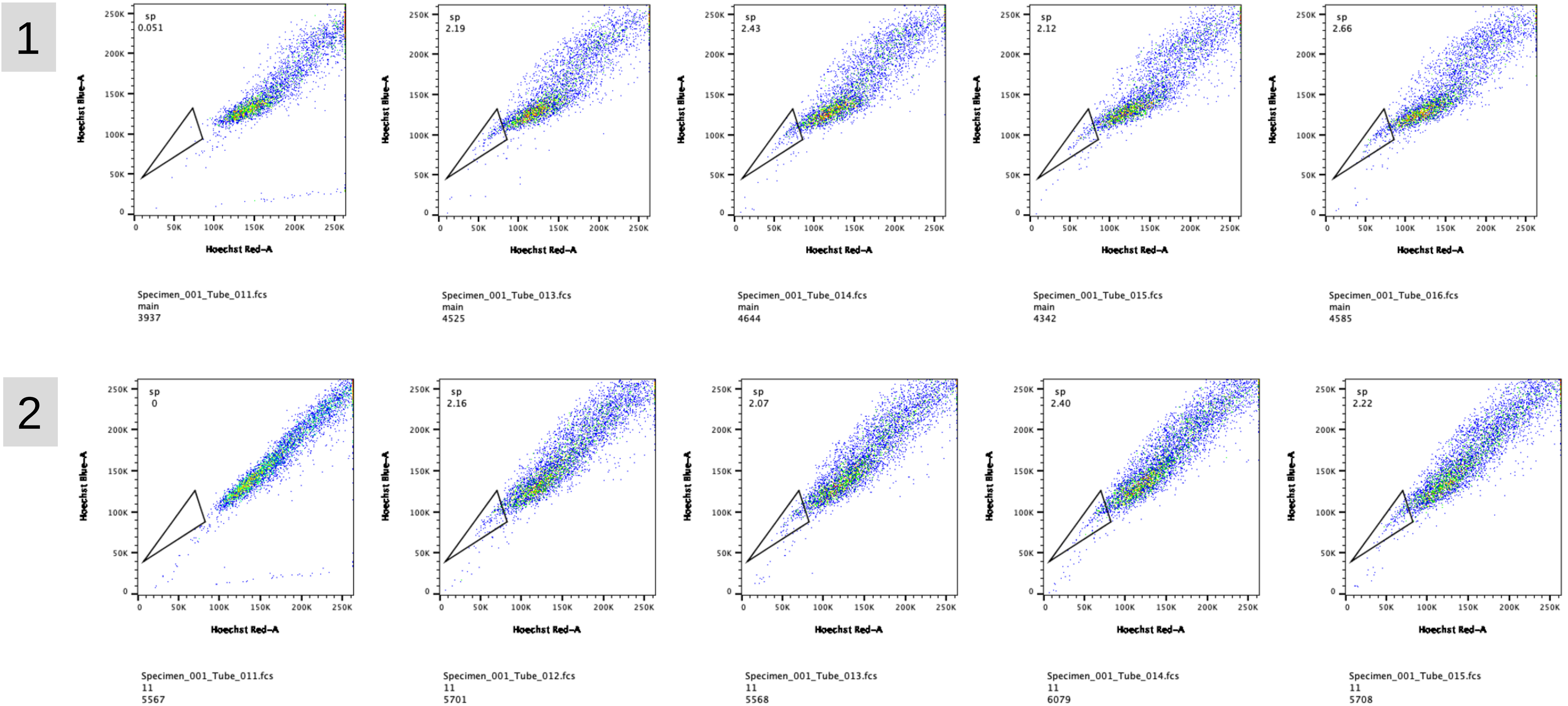

Supplement: S2 raw images — (PDF) [file pbio.3000872.s013.pdf]
