# Supplementary material for: Nucleotide de novo synthesis increases breast cancer stemness and metastasis via cGMP-PKG-MAPK signaling pathway
Source: PLoS Biol. 2020 Nov 13;18(11):e3000872. doi: 10.1371/journal.pbio.3000872 (PMC7688141; doi:10.1371/journal.pbio.3000872)

Full unedited gel for Figure 2C

CAD

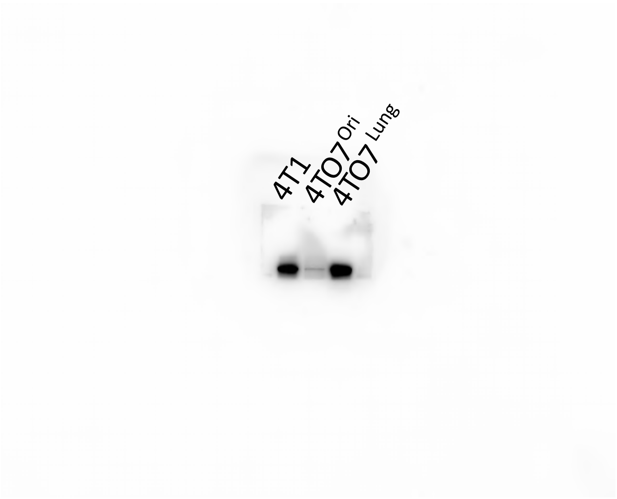

GMPS

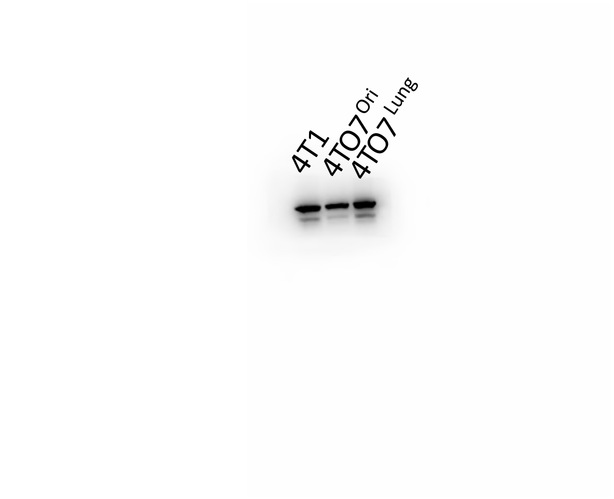

PFAS

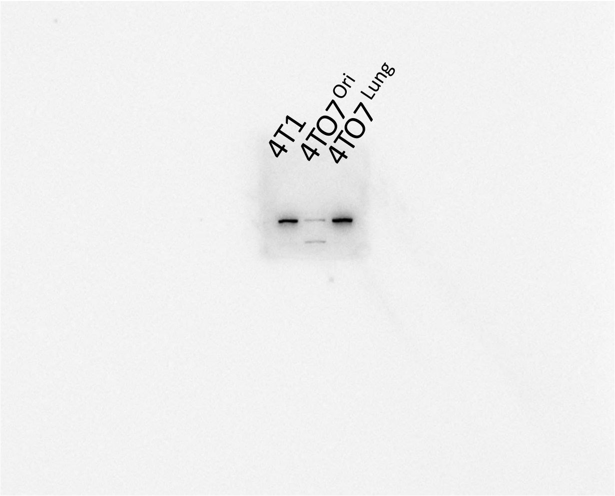

ADSS

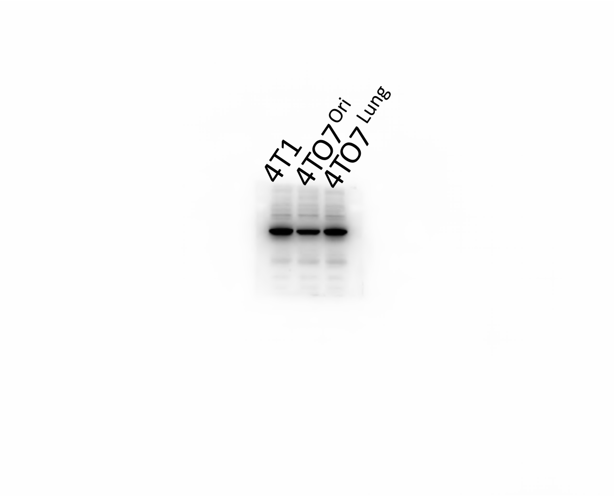

GART

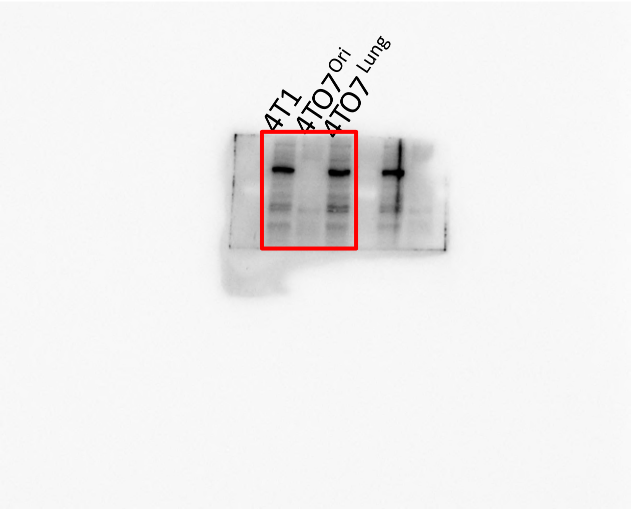

PRPS2

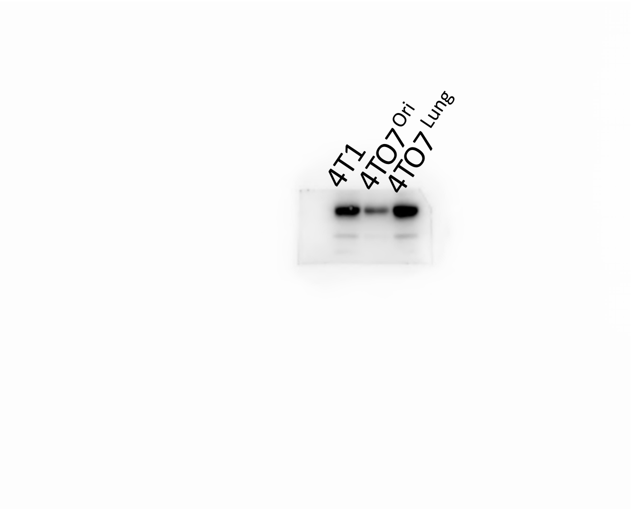

Full unedited gel for Figure 2C

ACTB

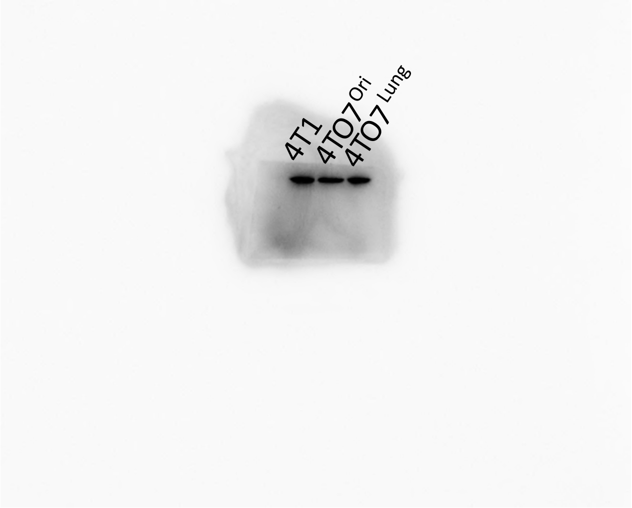

Full unedited gel for Figure 2F

PRPS2

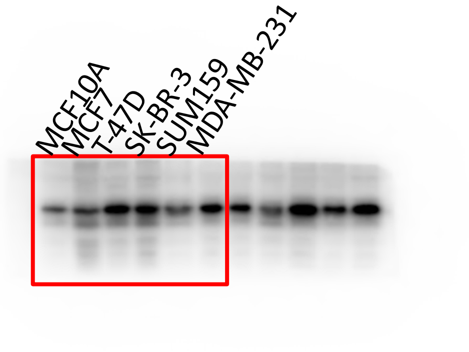

ACTB

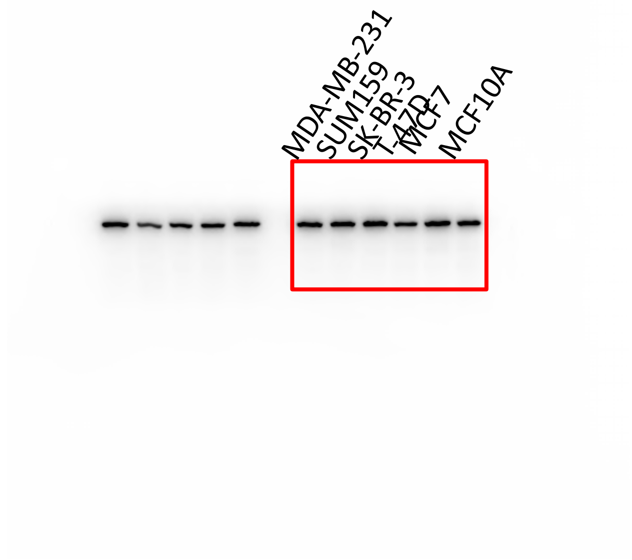

Full unedited gel for Figure 2H

PRPS2

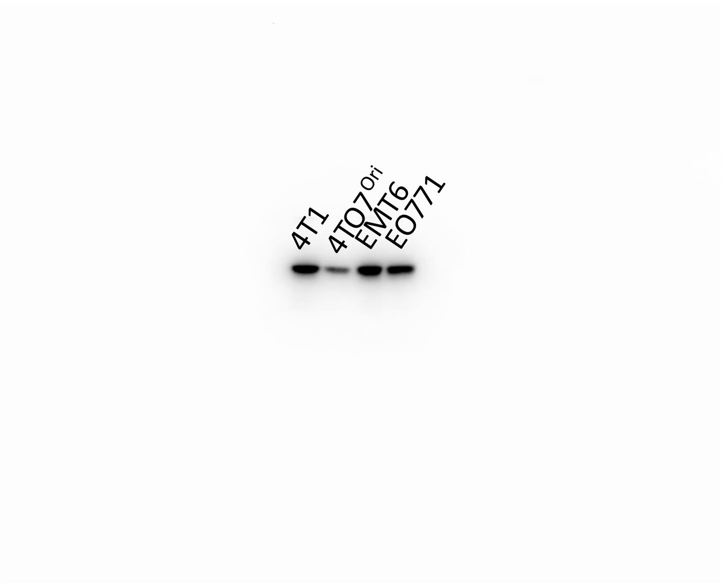

ACTB

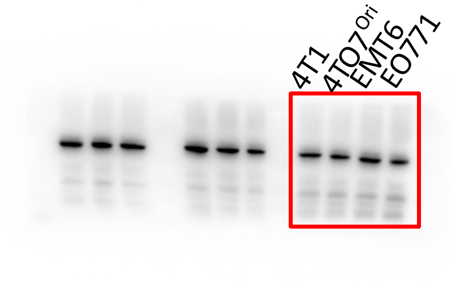

Full unedited gel for Figure 3B

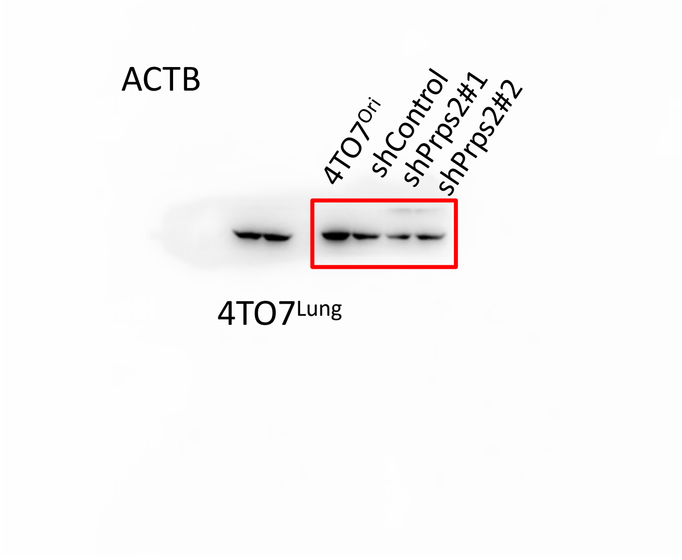

PRPS2

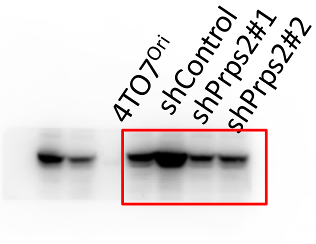

Full unedited gel for Figure 4F

KLF4

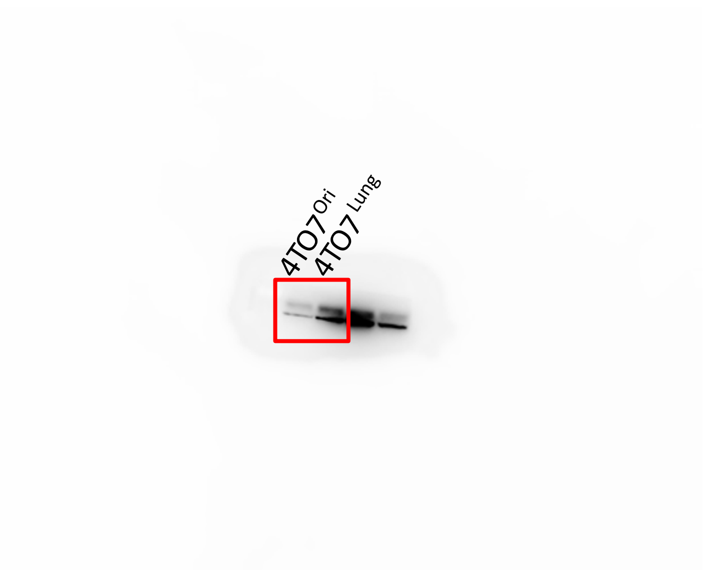

OCT4

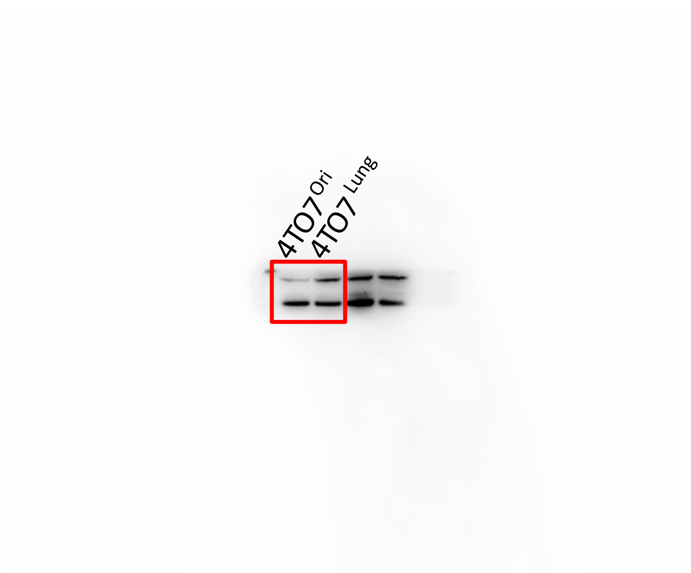

NANOG

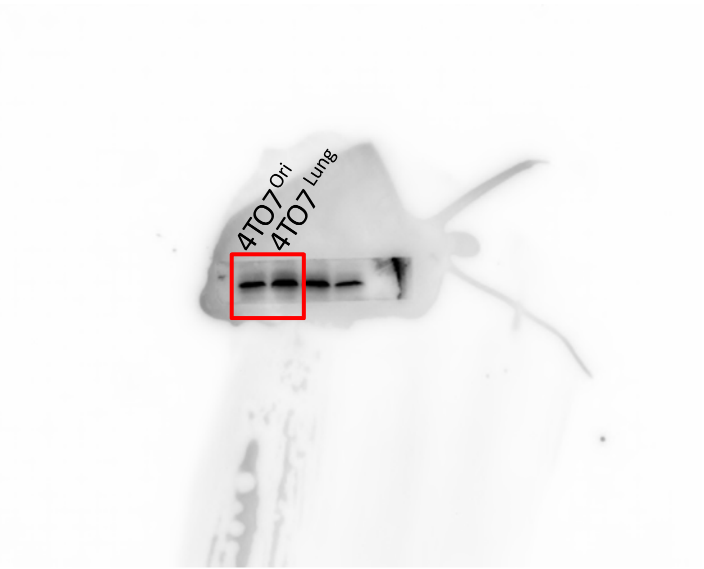

SOX2

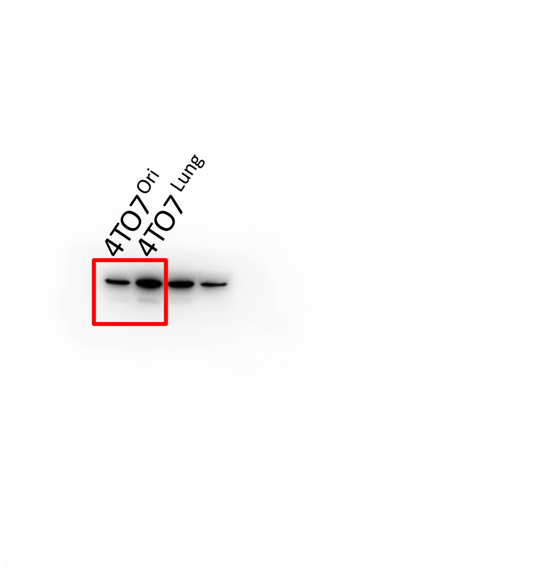

ACTB

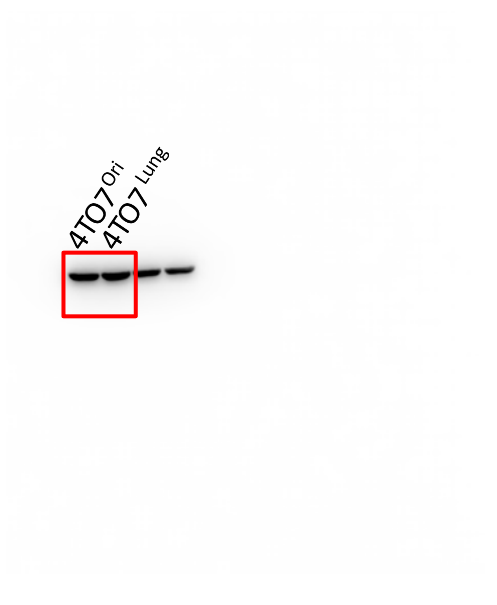

Full unedited gel for Figure 4P

KLF4

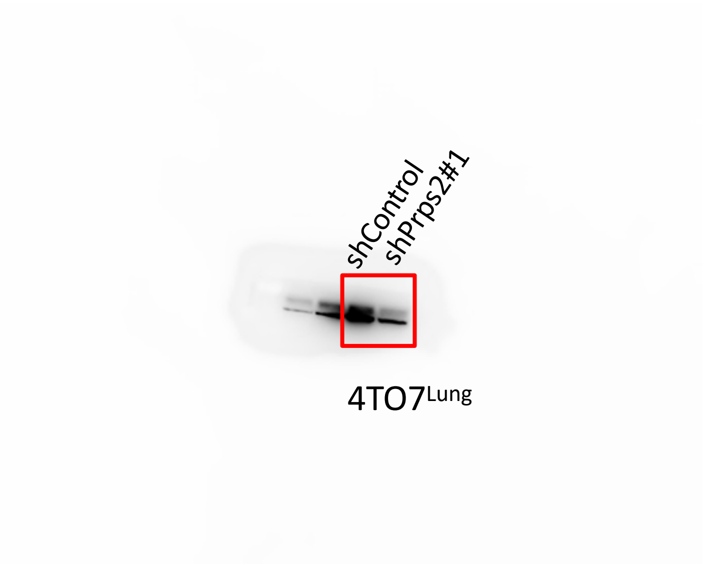

OCT4

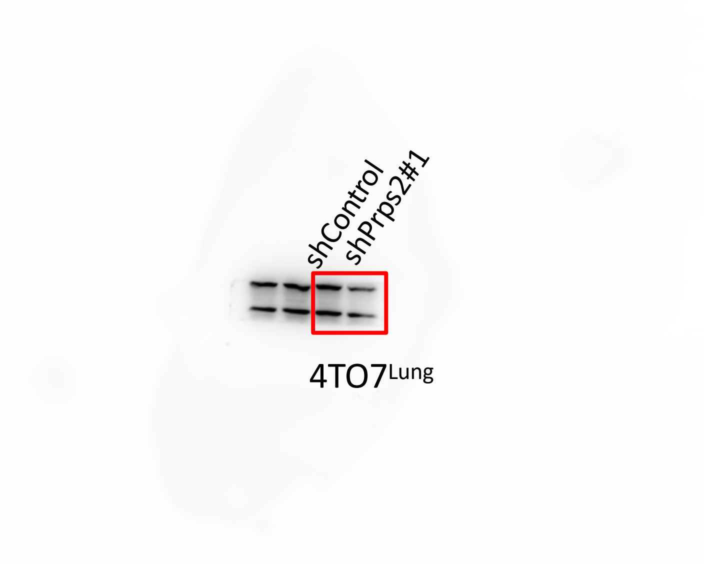

NANOG

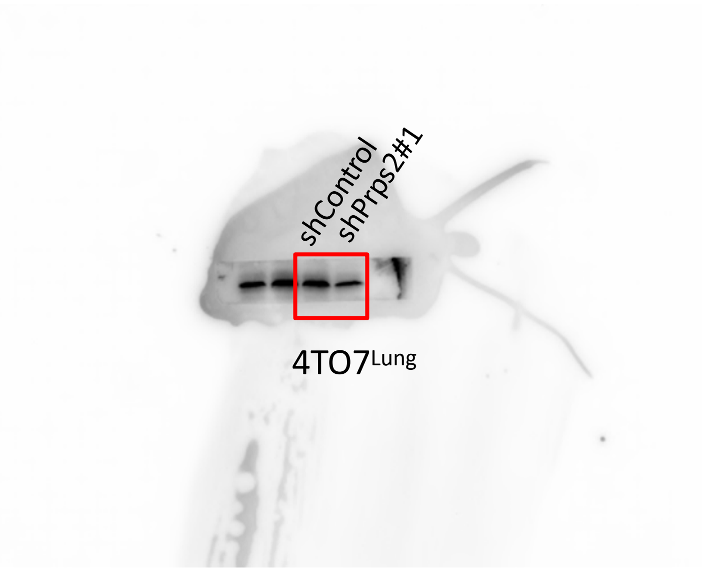

SOX2

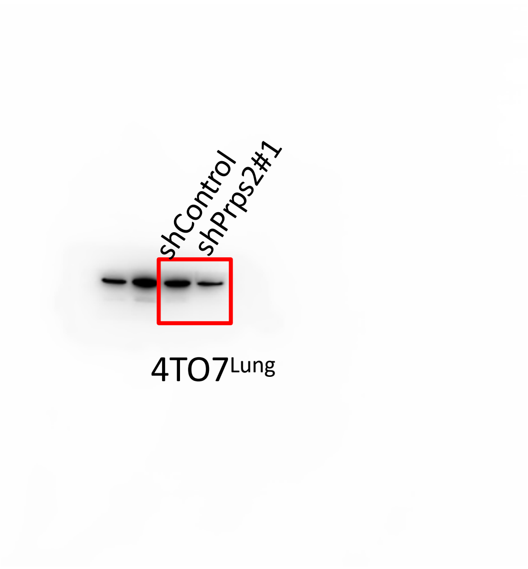

ACTB

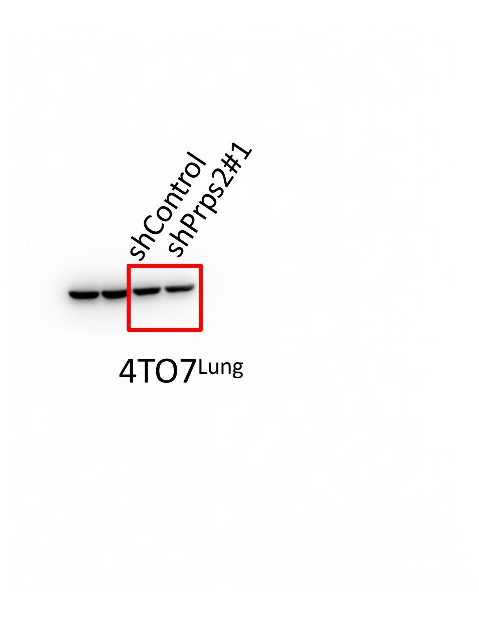

Full unedited gel for Figure 5E

P-VASP

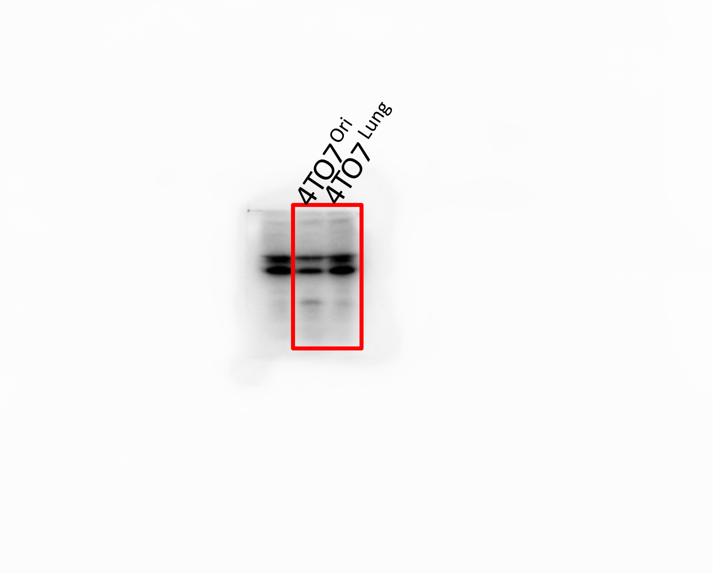

VASP

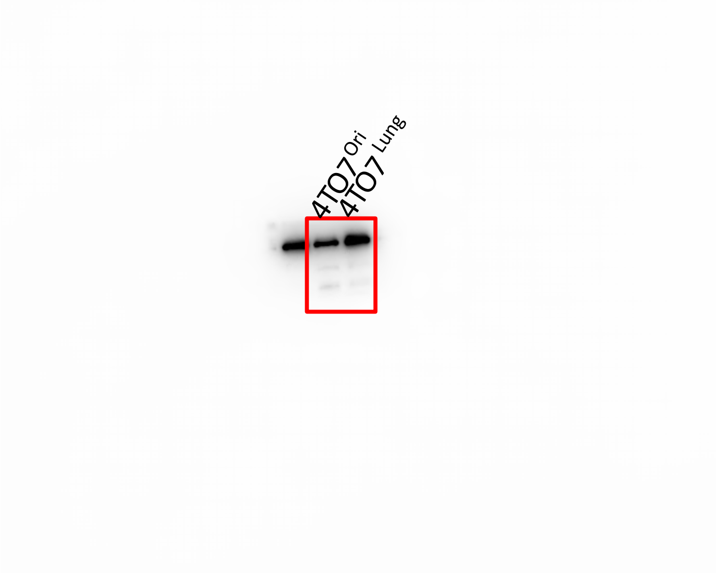

P-ERK

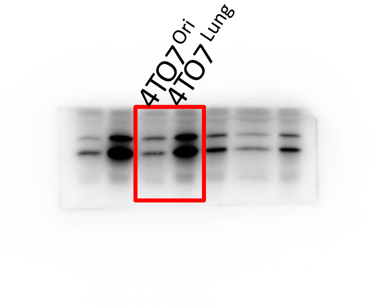

ERK1/2

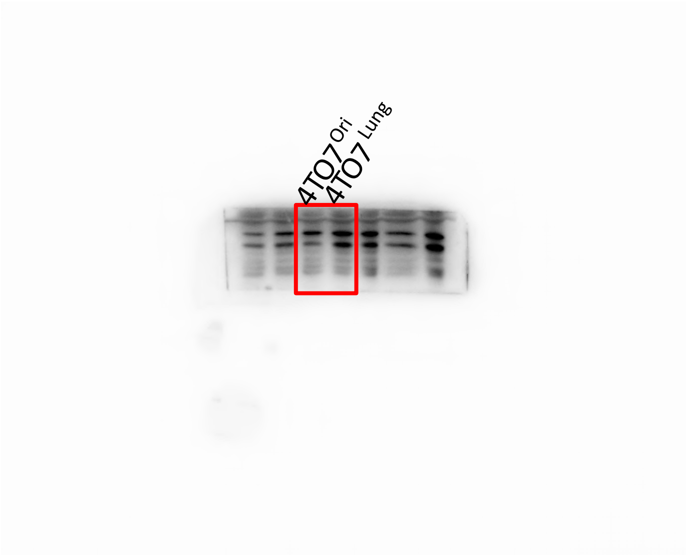

PRPS2

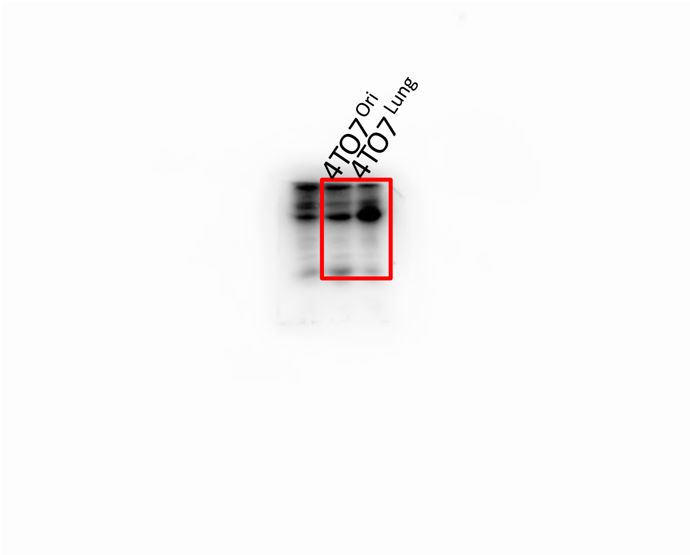

ACTB

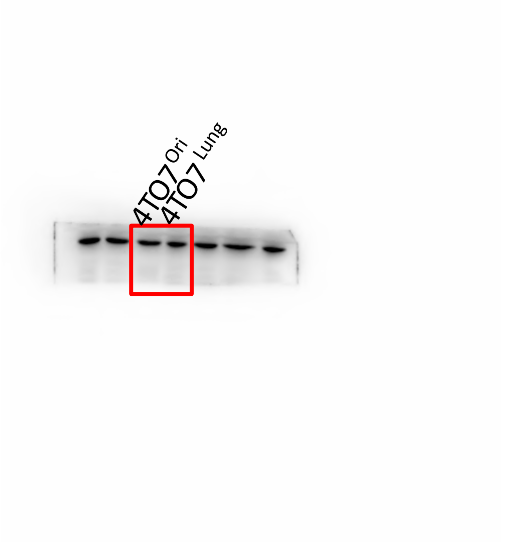

Full unedited gel for Figure 5F

P-VASP

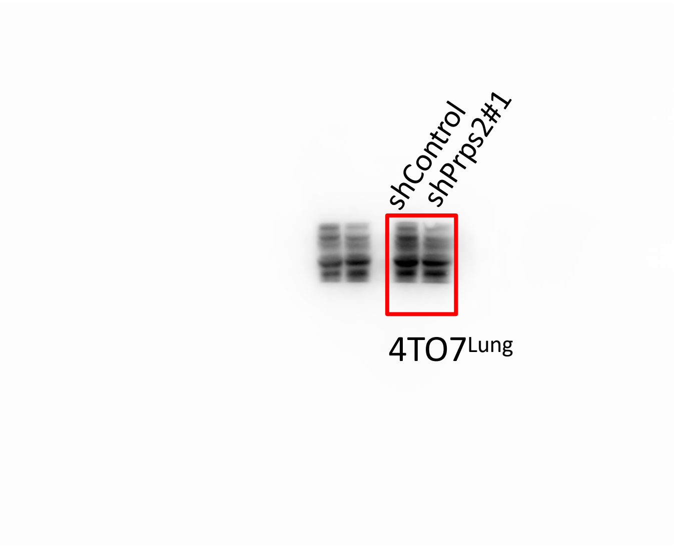

VASP

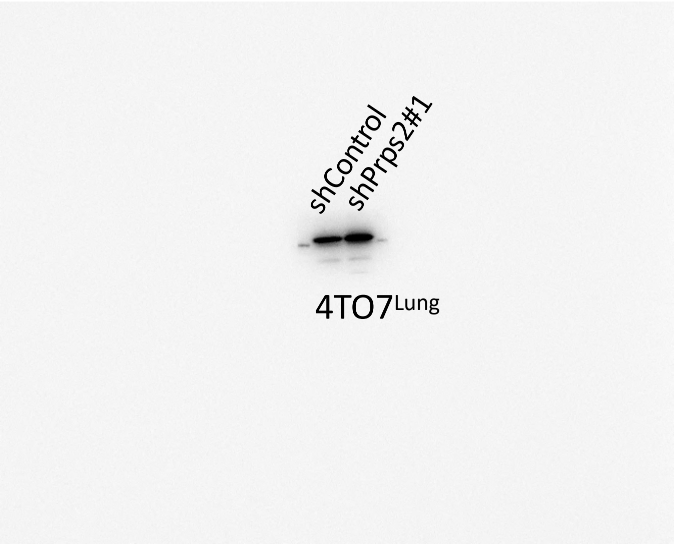

P-ERK

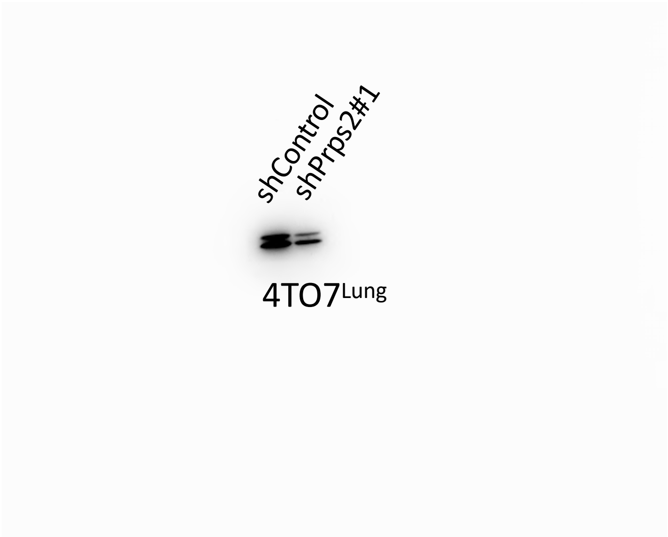

ERK1/2

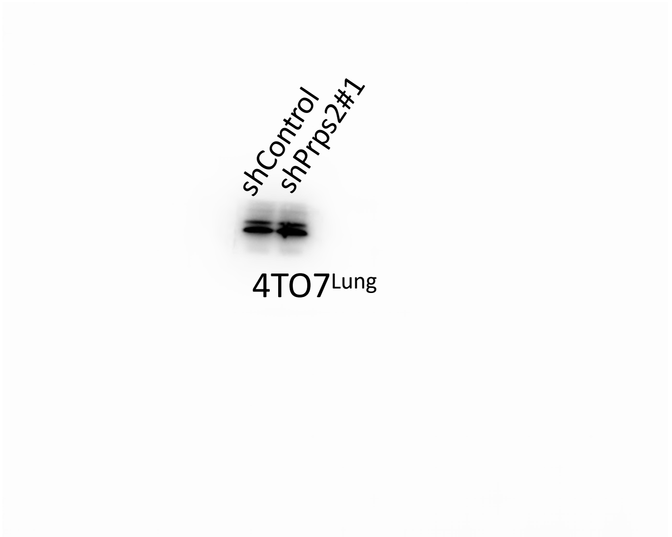

PRPS2

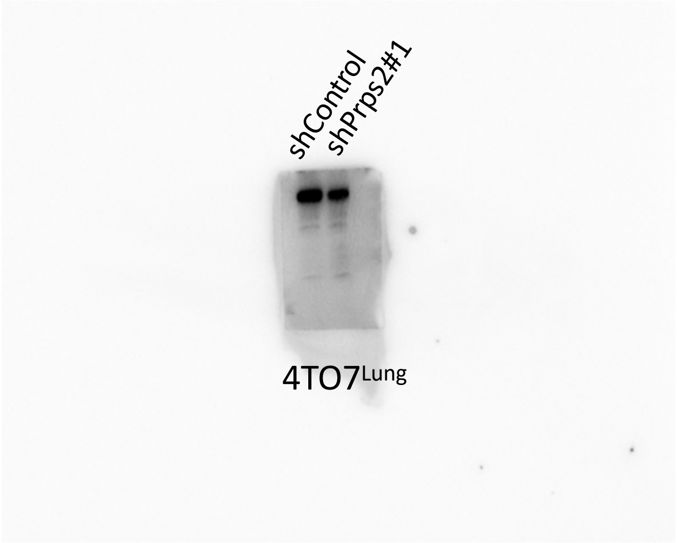

ACTB

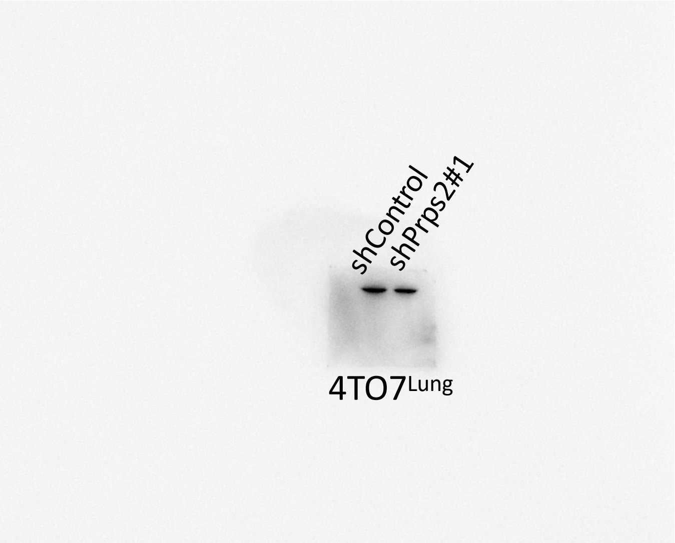

Full unedited gel for Figure 5H

KLF4

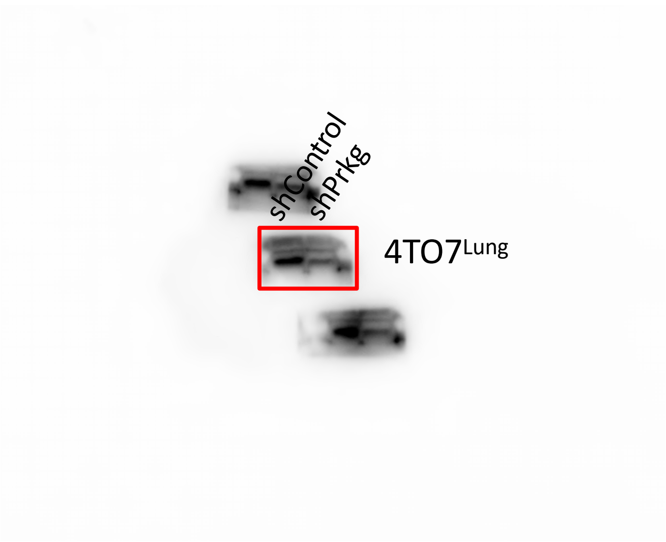

OCT4

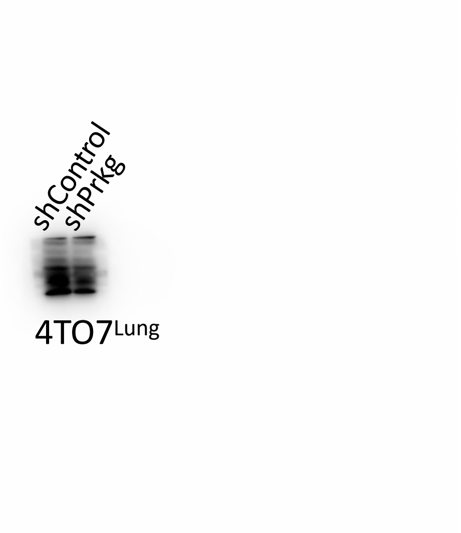

NANOG

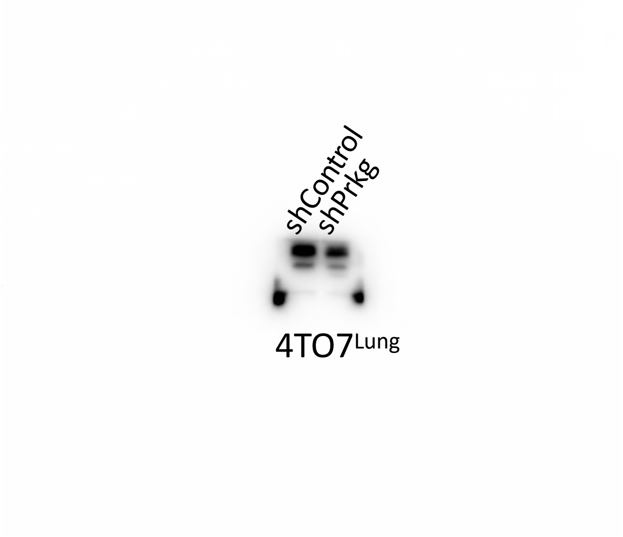

SOX2

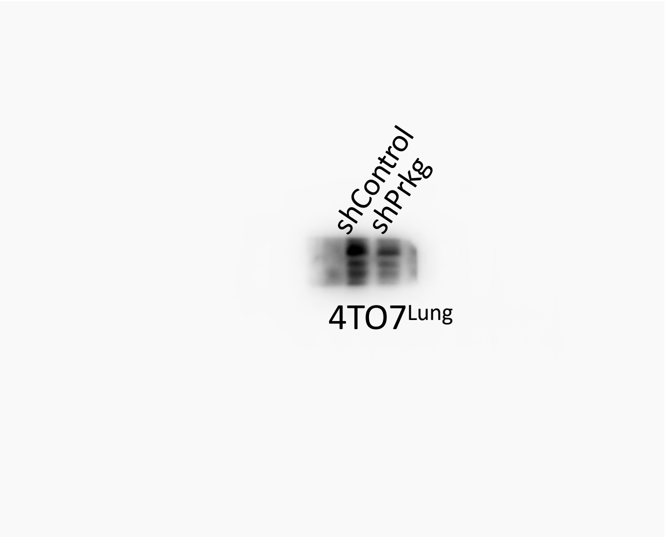

ACTB

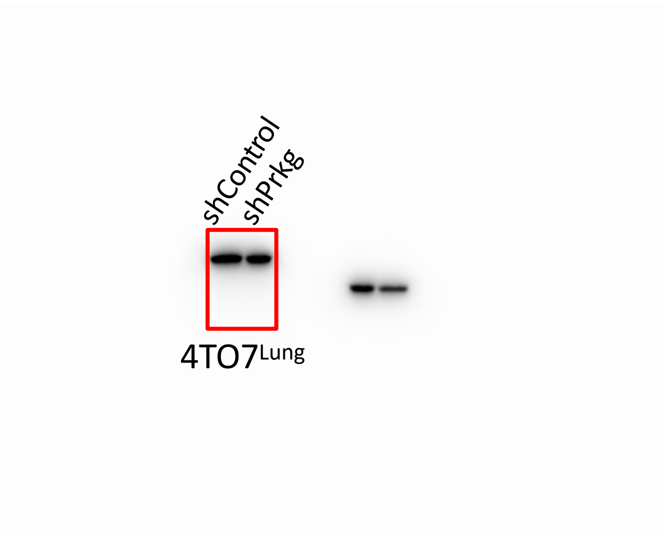

Full unedited gel for Figure 6D

KLF4

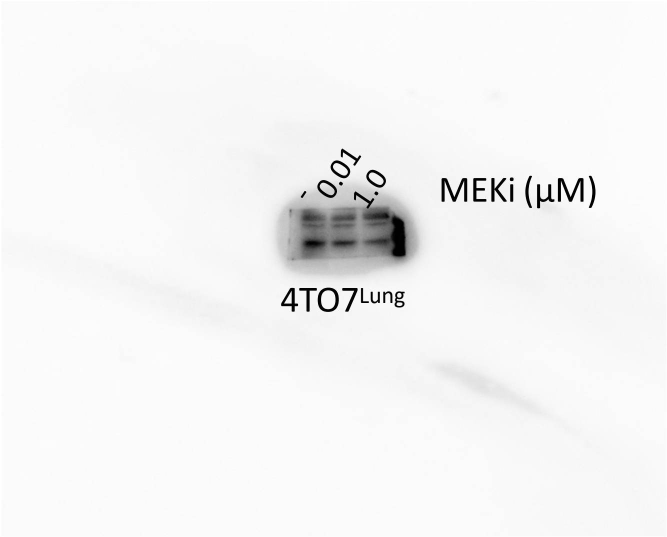

NANOG

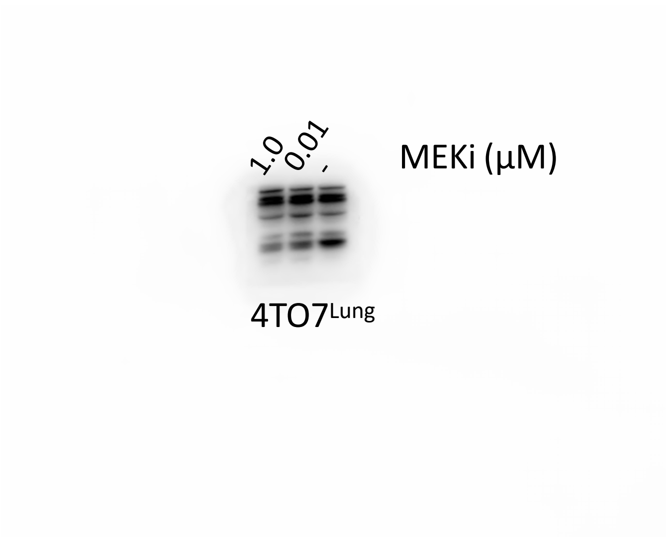

SOX2

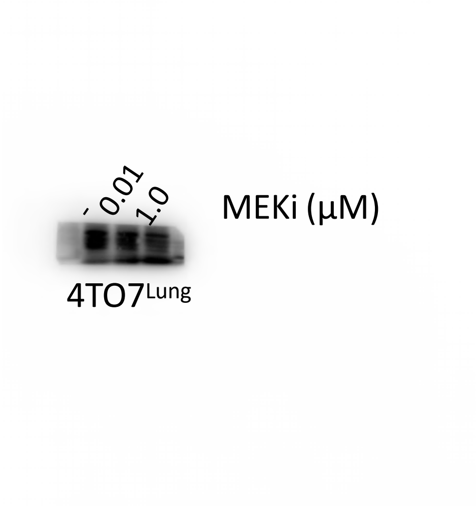

p-ERK

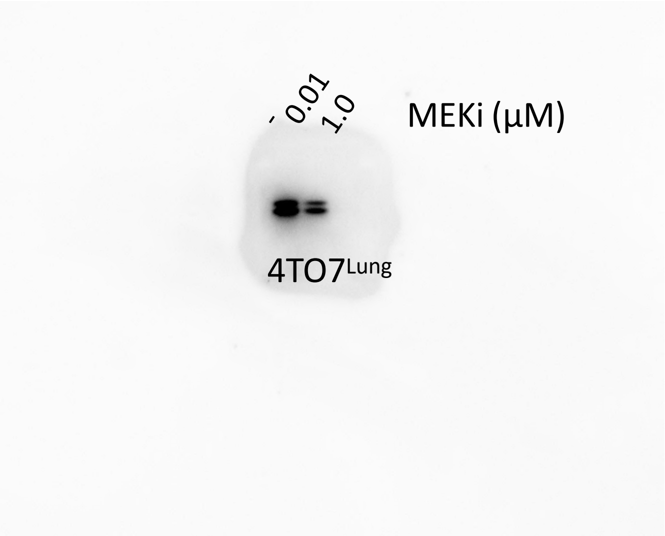

ERK1/2

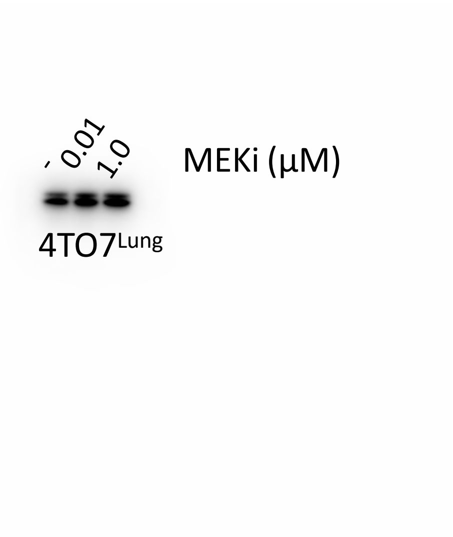

ACTB

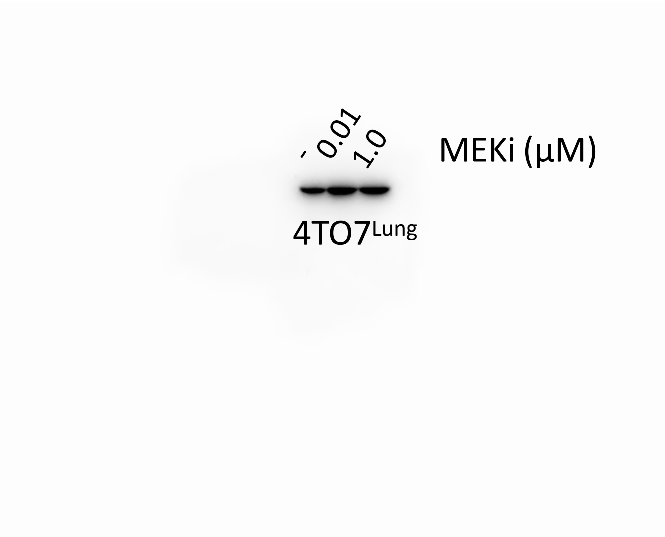

Full unedited gel for Figure 6E

KLF4

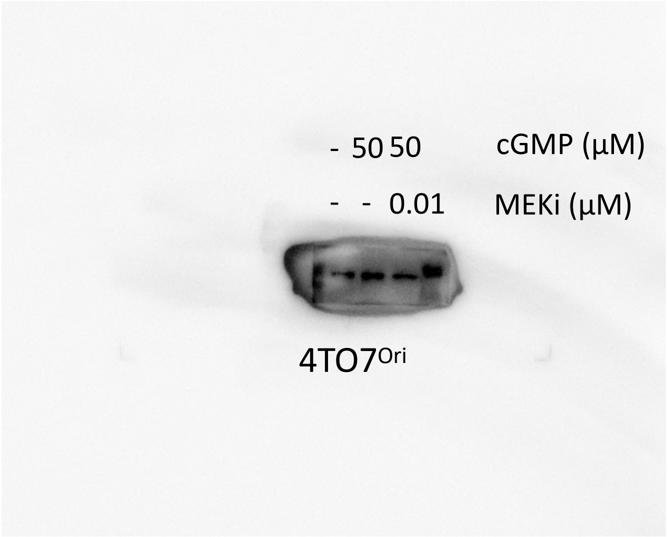

NANOG

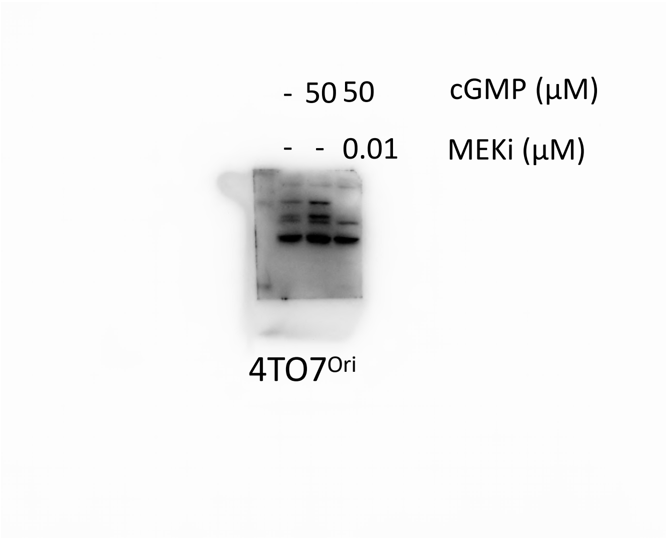

SOX2

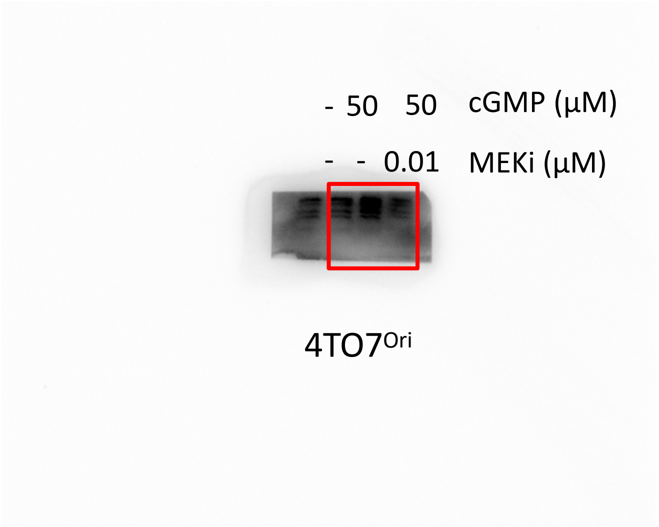

p-ERK

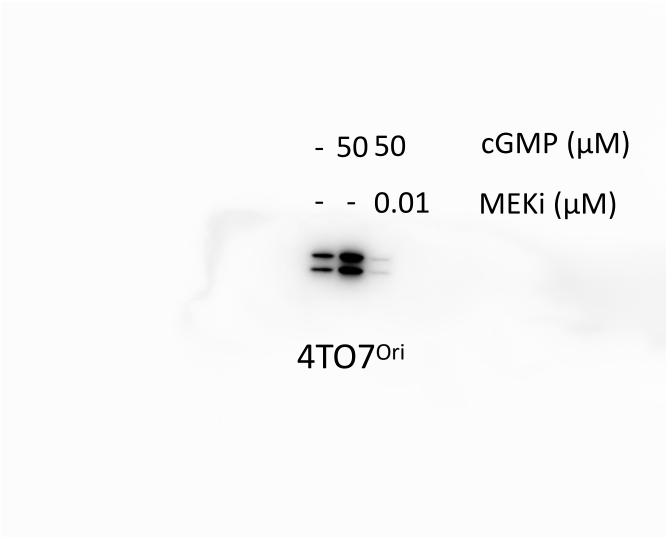

ERK1/2

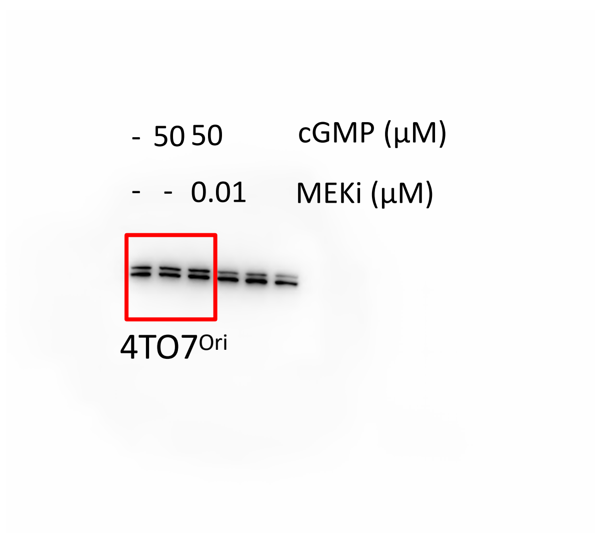

ACTB

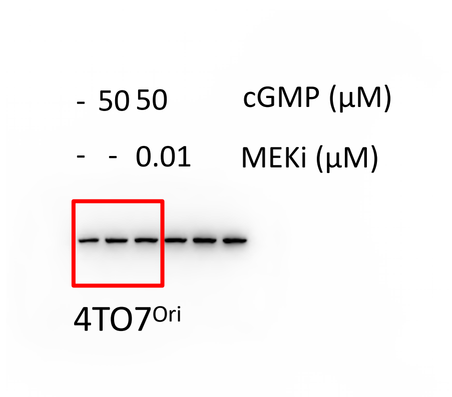

Full unedited gel for Figure 6F

KLF4

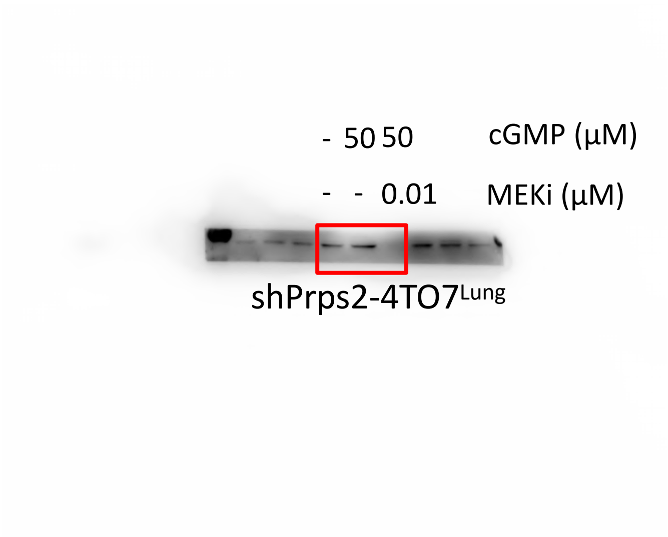

NANOG

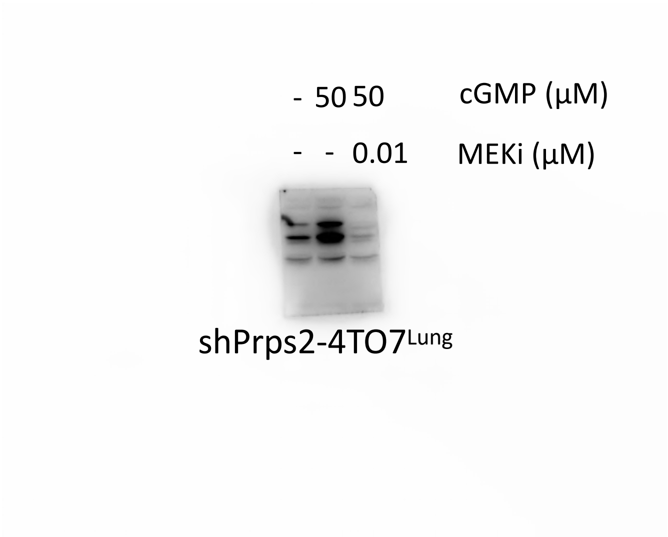

SOX2

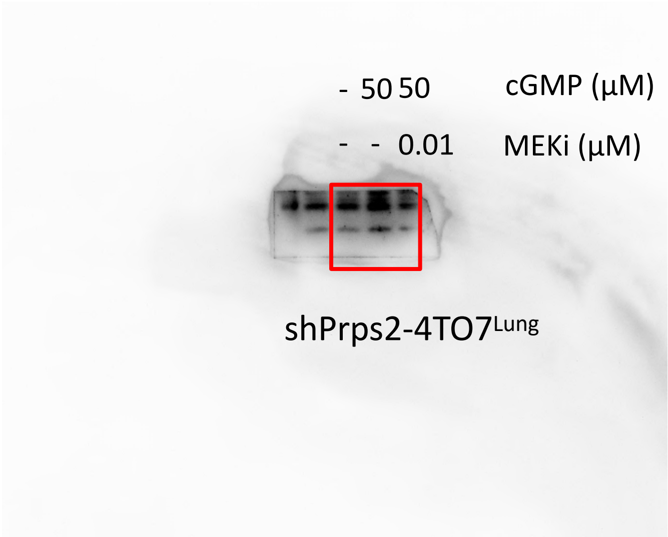

p-ERK

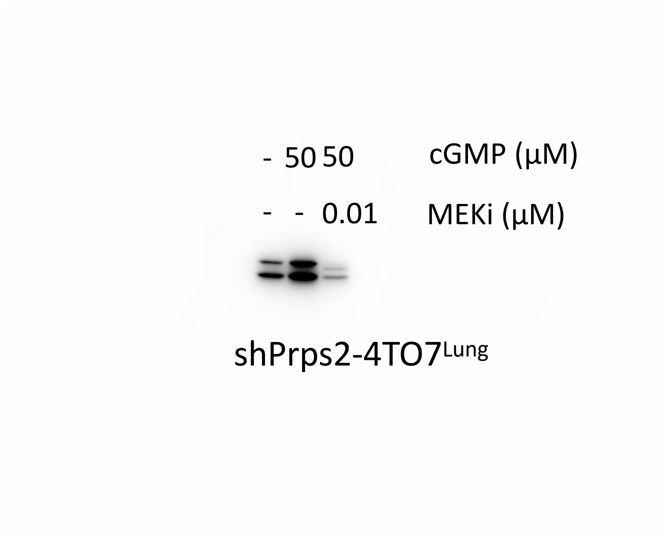

ERK1/2

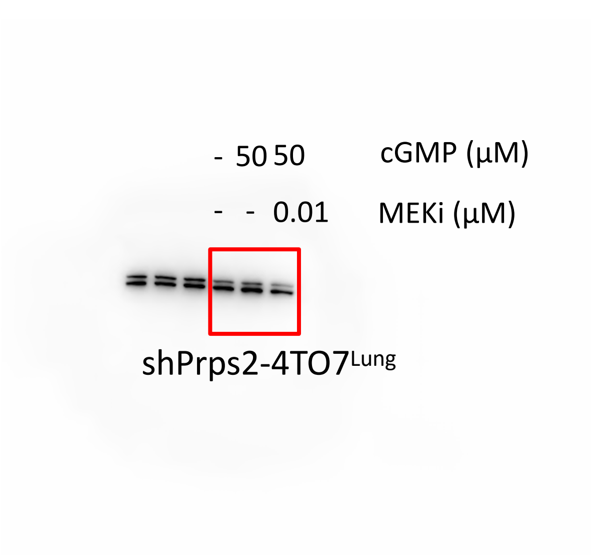

ACTB

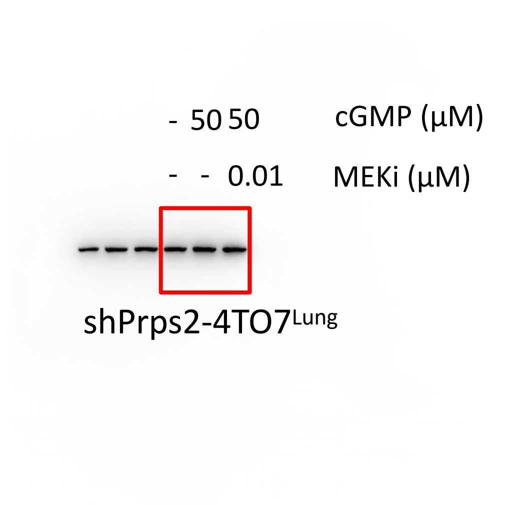

Full unedited gel for Figure S2B

CAD

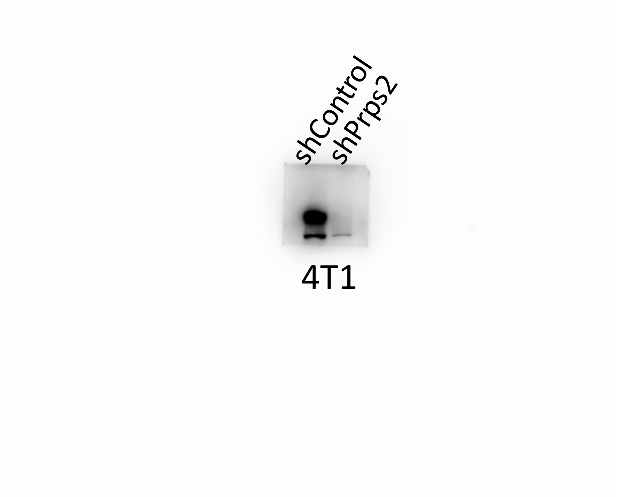

GMPS

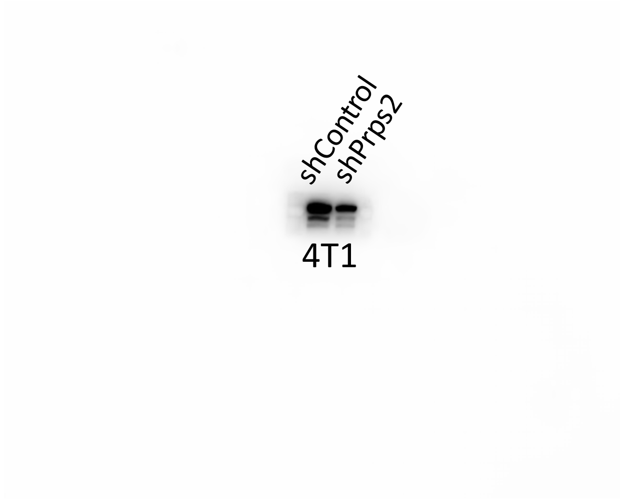

PFAS

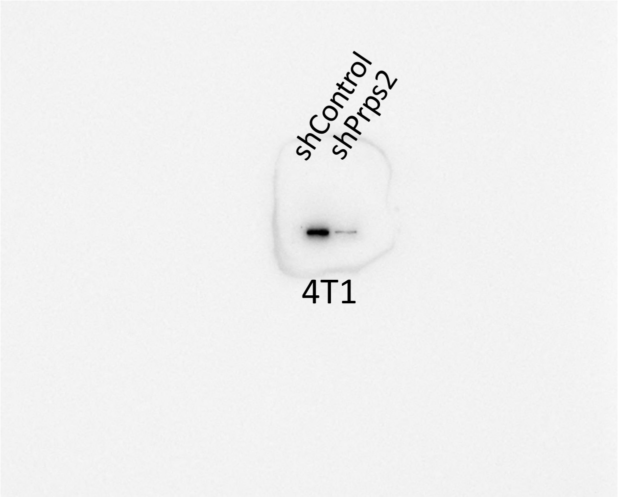

ADSS

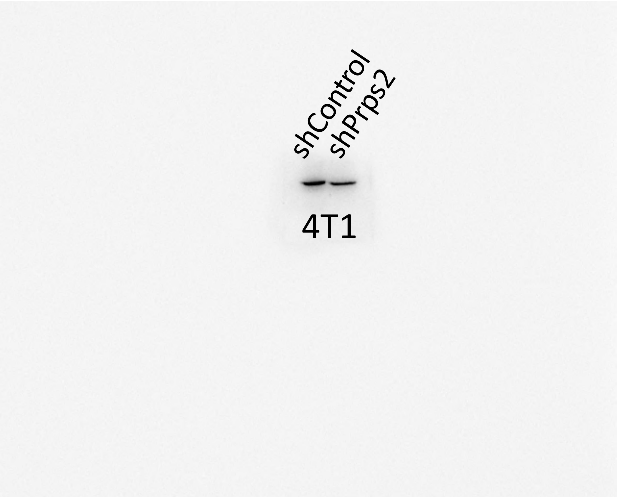

GART

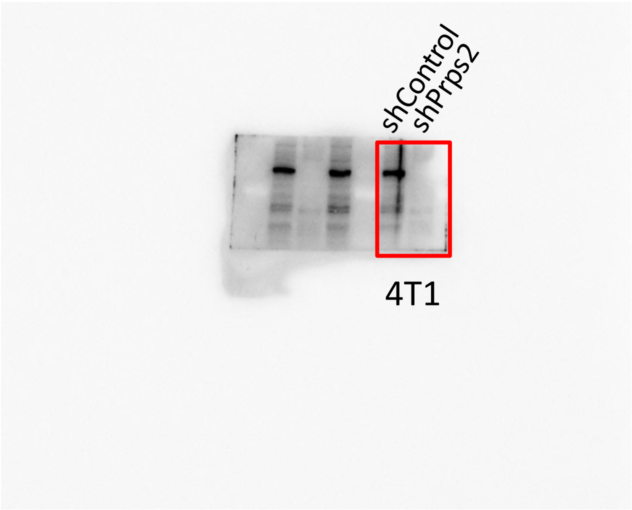

PRPS2

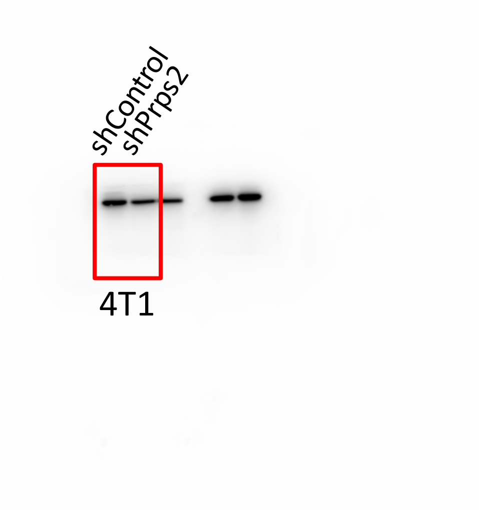

Full unedited gel for Figure S2B

ACTB

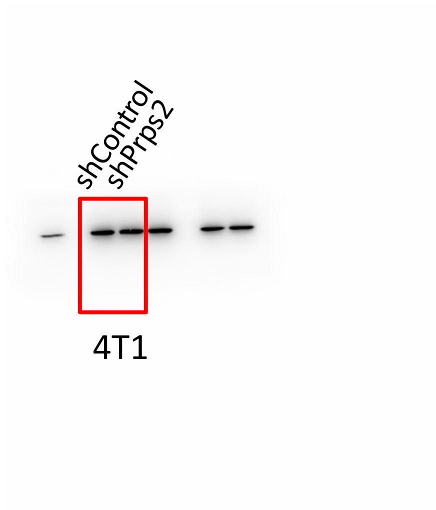

Full unedited gel for Figure S3C

ECAD

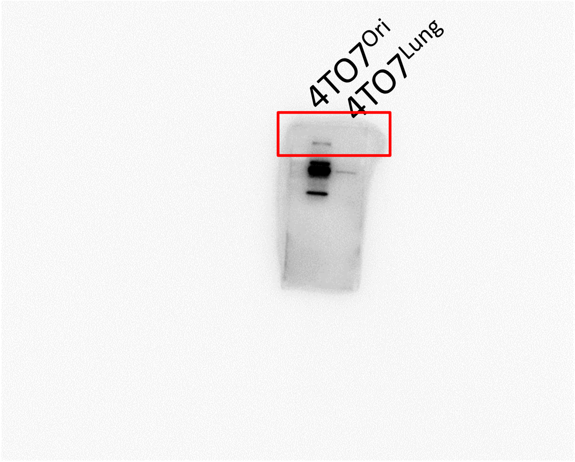

VIM

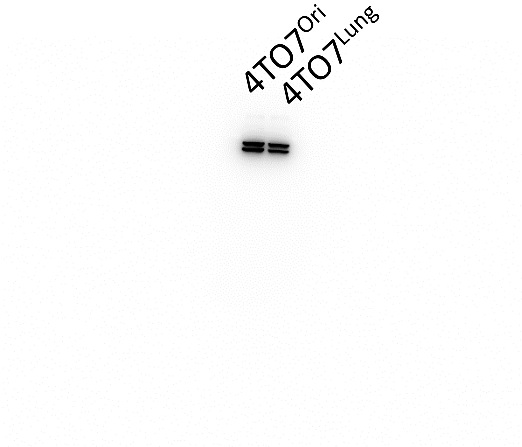

ZEB1

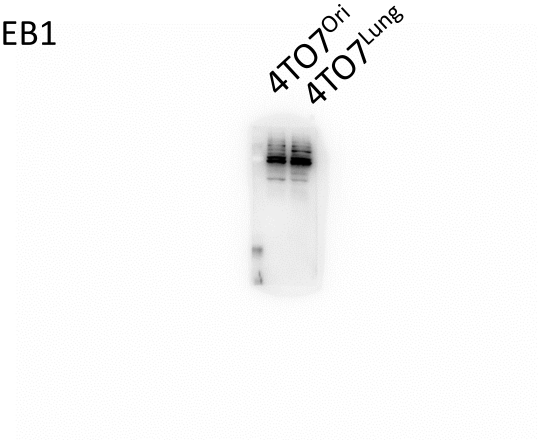

ACTB

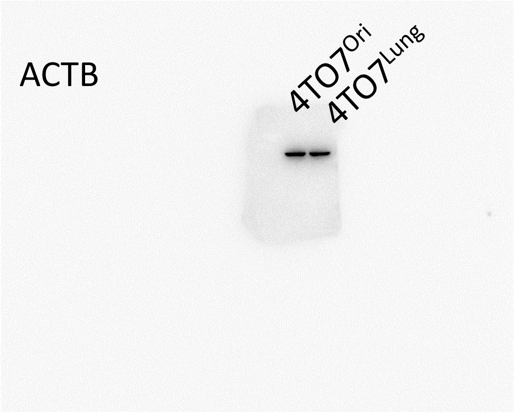

Full unedited gel for Figure S4F

OCT4

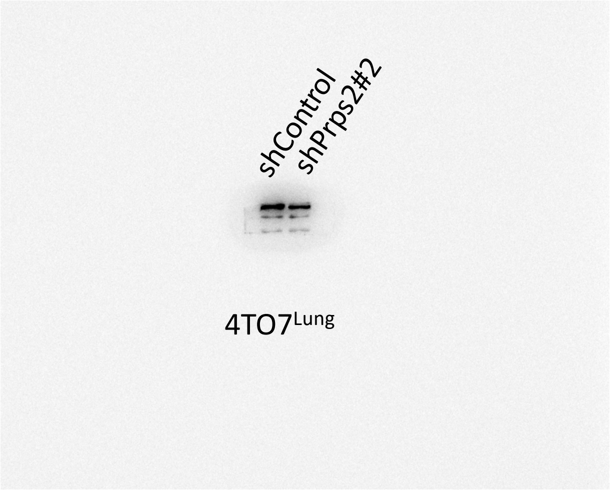

KLF4

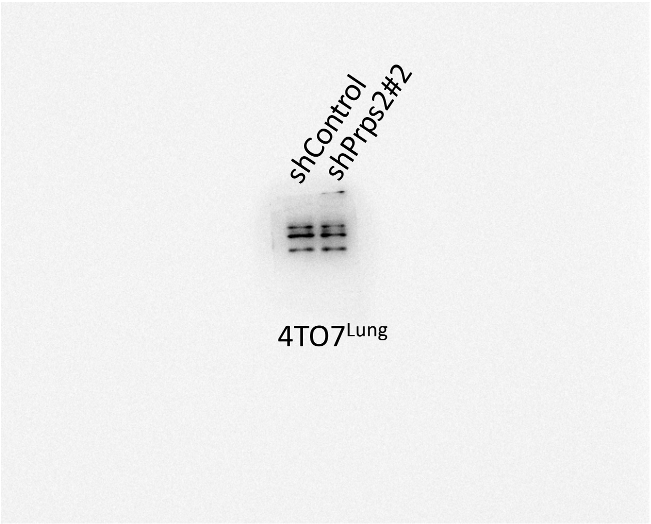

SOX2

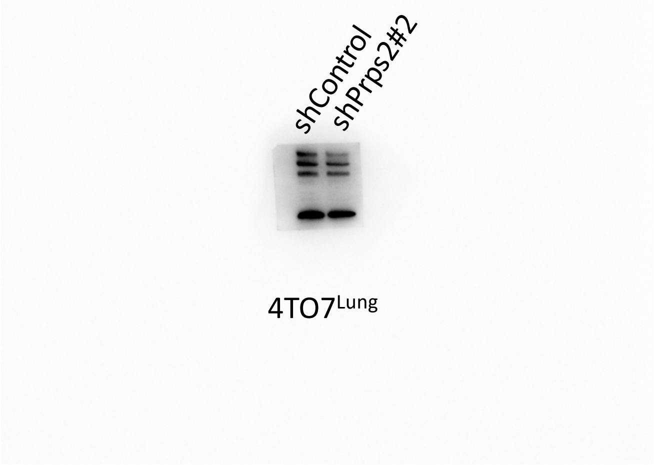

ACTB

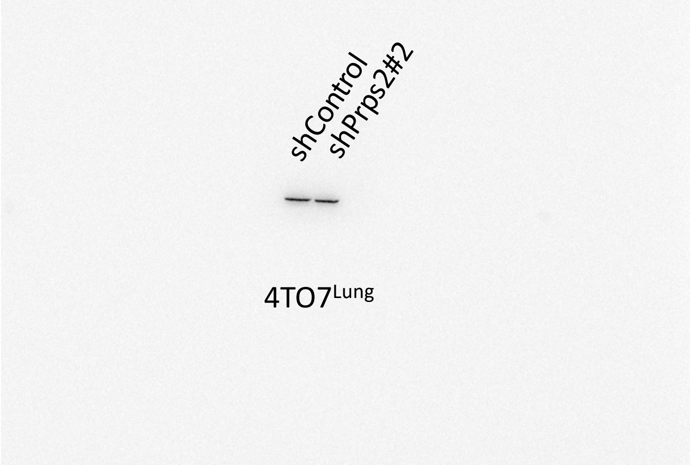

Full unedited gel for Figure S4G

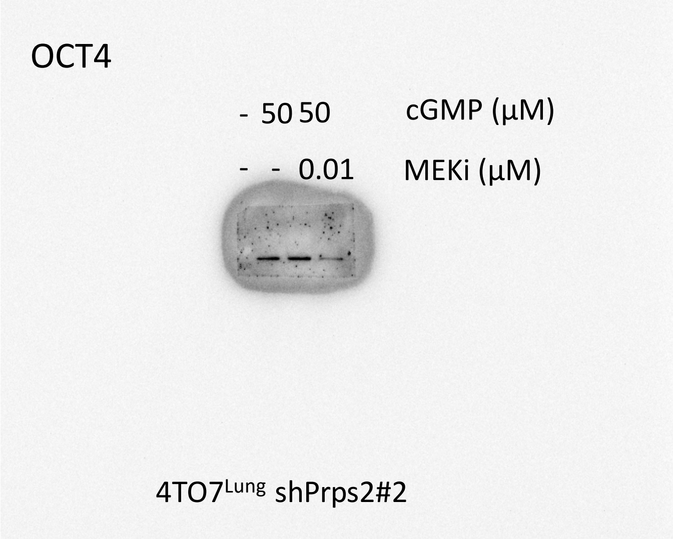

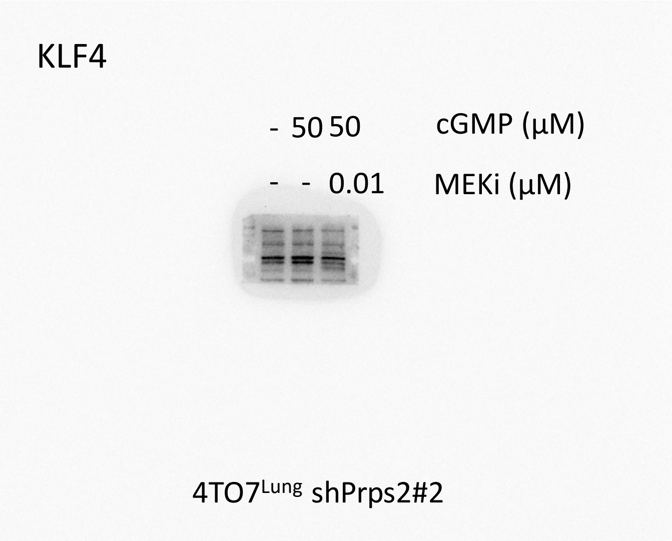

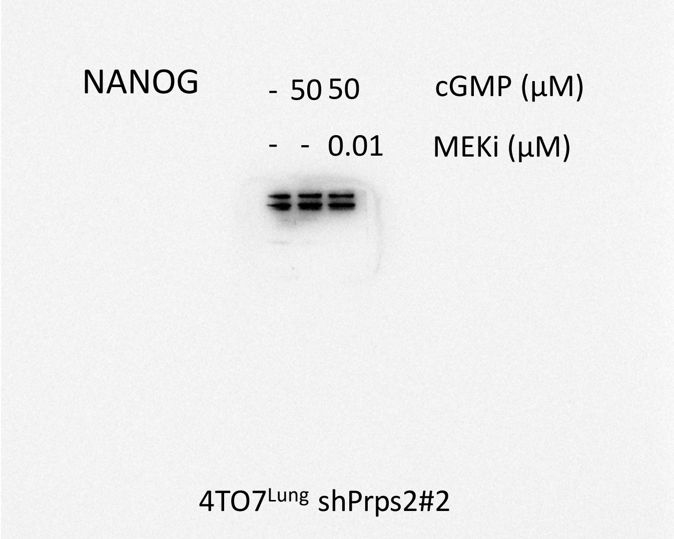

Full unedited gel for Figure S4G

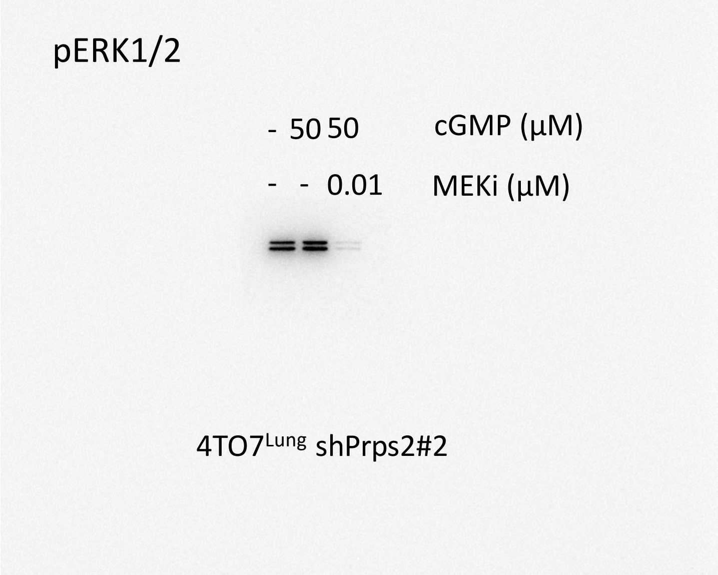

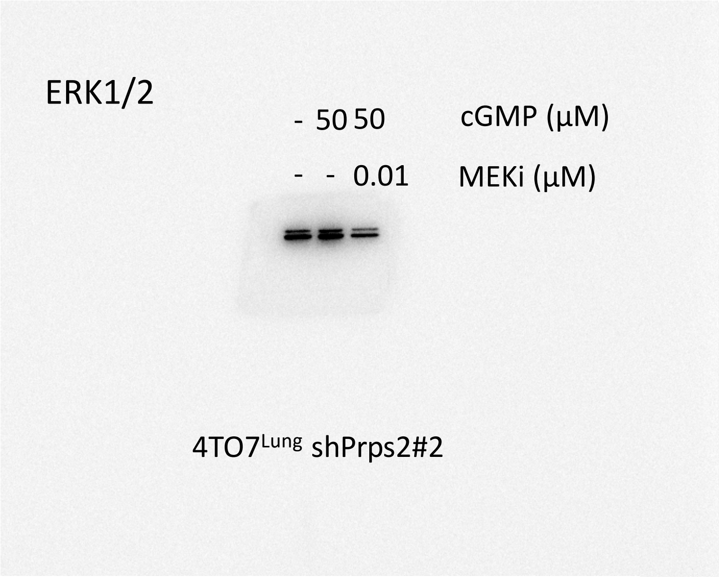

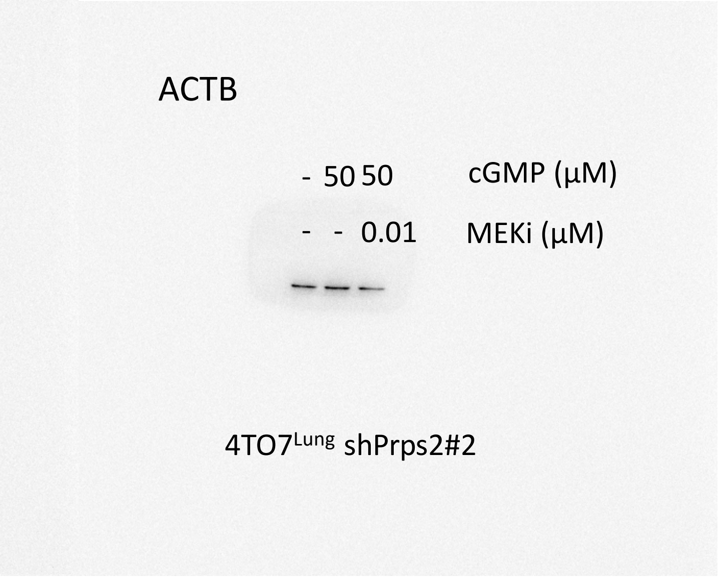

Supplement: S3 raw images — (PDF) [file pbio.3000872.s014.pdf]
