# Supplementary material for: Nucleotide de novo synthesis increases breast cancer stemness and metastasis via cGMP-PKG-MAPK signaling pathway
Source: PLoS Biol. 2020 Nov 13;18(11):e3000872. doi: 10.1371/journal.pbio.3000872 (PMC7688141; doi:10.1371/journal.pbio.3000872)
Supplement: S1 Fig Data — (PDF) [file pbio.3000872.s015.pdf]

The desired Affy ID is valid: 202715_at (CAD),

**Affy ID:**	202715_at	CAD
**Survival:**	RFS	
**Auto select best cutoff:**	checked	
**Follow up threshold:**	all	
**Censore at threshold:**	checked	
**Compute median over entire database:**	false	
**Cutoff value used in analysis:**	781	
**Expression range of the probe:**	11 - 5537	
**Probe set option:**	user selected probe set	
**Invert HR values below 1:**	not checked	

Restrictions

ER status:	all
derive ER status from gene expression data:	not checked
PR status:	all
HER2 status:	all
Intrinsic subtype:	all
Lymph node status:	all
Grade:	all
TP53 status:	all
Pietenpol subtype:	all
Use earlier release of the database:	all
Use following dataset for the analysis:	all

Quality control

Remove redundant samples:	checked
Array quality control:	exclude biased arrays
Proportional hazards assumption:	0

Cohort

Cohorts: not selected

Results

P value: 0.0003
FDR: 20%

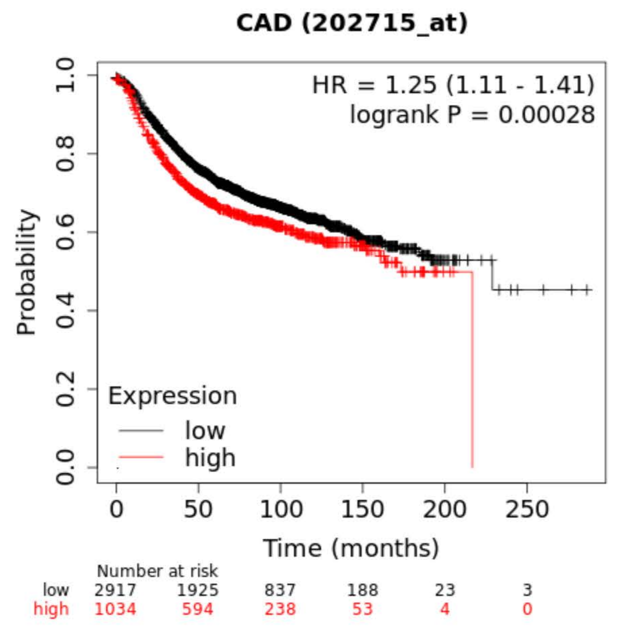

Download plot as a PDF

Download p values vs. cutoff table

Median survival

Low expression cohort (months)	High expression cohort (months)
228.85	173.2

Restart

You can save the plots by right-clicking the image and then selecting "Save image as...". To generate a high resolution TIFF image, please adjust the "Settings" in the analysis page.

KMplot.com Copyright © 2009-2020 | Contact | Sitemap

The desired Affy ID is valid: 214431_at (GMPS),

**Affy ID:**	214431_at	GMPS
**Survival:**	RFS	
**Split patients by:**	median	
**Follow up threshold:**	all	
**Censore at threshold:**	checked	
**Compute median over entire database:**	false	
**Cutoff value used in analysis:**	937	
**Expression range of the probe:**	186 - 8103	
**Probe set option:**	user selected probe set	
**Invert HR values below 1:**	not checked	

Restrictions

ER status:	all
derive ER status from gene expression data:	not checked
PR status:	all
HER2 status:	all
Intrinsic subtype:	all
Lymph node status:	all
Grade:	all
TP53 status:	all
Pietenpol subtype:	all
Use earlier release of the database:	all
Use following dataset for the analysis:	all

Quality control

Remove redundant samples:	checked
Array quality control:	exclude biased arrays
Proportional hazards assumption:	0

Cohort

Cohorts: not selected

Results

P value: <1e-16

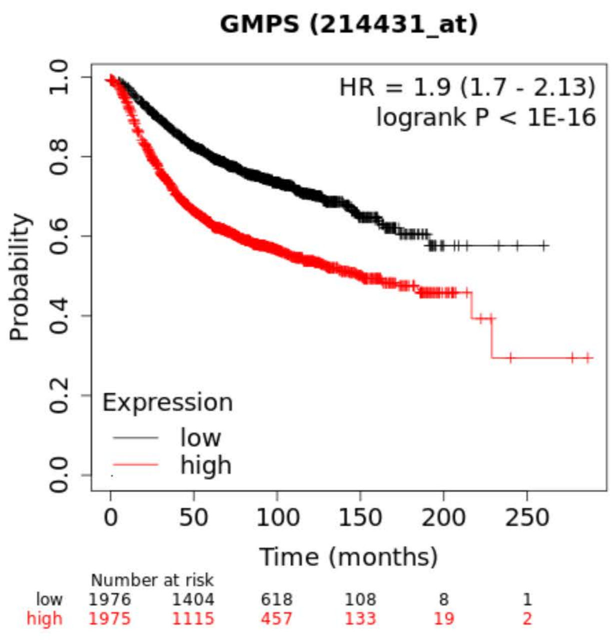

Download plot as a PDF

Upper quartile survival

Low expression cohort (months)	High expression cohort (months)
89.52	32

Restart

You can save the plots by right-clicking the image and then selecting "Save image as...". To generate a high resolution TIFF image, please adjust the "Settings" in the analysis page.

KMplot.com Copyright © 2009-2020 | Contact | Sitemap

The desired Affy ID is valid: 201013_s_at (PAICS),

**Affy ID:**	201013_s_at	PAICS, AIRC, ADE2H1, ...
**Survival:**	RFS	
**Split patients by:**	median	
**Follow up threshold:**	all	
**Censore at threshold:**	checked	
**Compute median over entire database:**	false	
**Cutoff value used in analysis:**	2333	
**Expression range of the probe:**	502 - 11326	
**Probe set option:**	user selected probe set	
**Invert HR values below 1:**	not checked	

Restrictions

ER status:	all
derive ER status from gene expression data:	not checked
PR status:	all
HER2 status:	all
Intrinsic subtype:	all
Lymph node status:	all
Grade:	all
TP53 status:	all
Pietenpol subtype:	all
Use earlier release of the database:	all
Use following dataset for the analysis:	all

Quality control

Remove redundant samples:	checked
Array quality control:	exclude biased arrays
Proportional hazards assumption:	0

Cohort

Cohorts: not selected

Results

P value: 1.7e-7

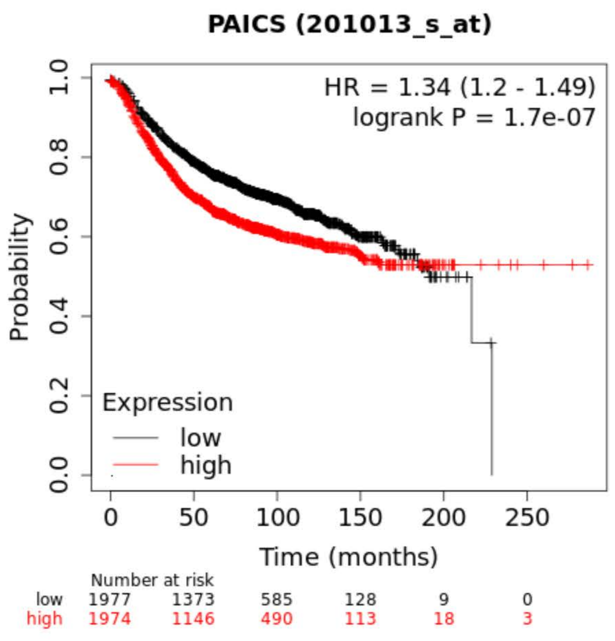

Download plot as a PDF

Upper quartile survival

Low expression cohort (months)	High expression cohort (months)
66	38.2

Restart

You can save the plots by right-clicking the image and then selecting "Save image as...". To generate a high resolution TIFF image, please adjust the "Settings" in the analysis page.

KMplot.com Copyright © 2009-2020 | Contact | Sitemap

The desired Affy ID is valid: 203401_at (PRPS2),

**Affy ID:**	203401_at	PRPS2
**Survival:**	RFS	
**Split patients by:**	median	
**Follow up threshold:**	all	
**Censore at threshold:**	checked	
**Compute median over entire database:**	false	
**Cutoff value used in analysis:**	825	
**Expression range of the probe:**	37 - 6240	
**Probe set option:**	user selected probe set	
**Invert HR values below 1:**	not checked	

Restrictions

ER status:	all
derive ER status from gene expression data:	not checked
PR status:	all
HER2 status:	all
Intrinsic subtype:	all
Lymph node status:	all
Grade:	all
TP53 status:	all
Pietenpol subtype:	all
Use earlier release of the database:	all
Use following dataset for the analysis:	all

Quality control

Remove redundant samples:	checked
Array quality control:	exclude biased arrays
Proportional hazards assumption:	0

Cohort

Cohorts: not selected

Results

P value: 1.1e-5

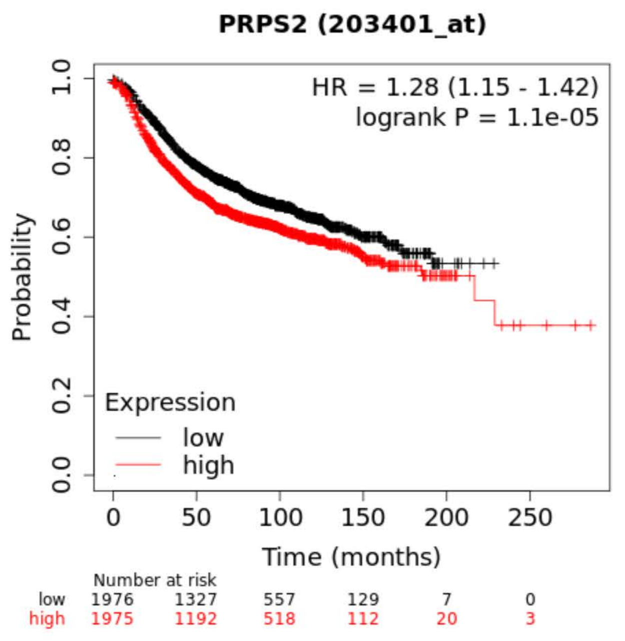

Download plot as a PDF

Upper quartile survival

Low expression cohort (months)	High expression cohort (months)
60.6	39.95

Restart

You can save the plots by right-clicking the image and then selecting "Save image as...". To generate a high resolution TIFF image, please adjust the "Settings" in the analysis page.

KMplot.com Copyright © 2009-2020 | Contact | Sitemap
